# Heterobimetallic Coinage Metal‐Ruthenium Complexes Supported by Anionic N‐Heterocyclic Carbenes

**DOI:** 10.1002/chem.202102553

**Published:** 2021-09-03

**Authors:** Sebastian Planer, Jenni Frosch, Marvin Koneczny, Damian Trzybiński, Krzysztof Woźniak, Karol Grela, Matthias Tamm

**Affiliations:** ^1^ Institut für Anorganische und Analytische Chemie Technische Universität Braunschweig Hagenring 30 38106 Braunschweig Germany; ^2^ Biological and Chemical Research Centre, Faculty of Chemistry University of Warsaw Żwirki i Wigury 101 02-089 Warsaw Poland

**Keywords:** Copper, Heterobimetallic Complexes, N-Heterocyclic Carbenes, Ruthenium, Silver

## Abstract

The lithium complexes [(WCA‐NHC)Li(toluene)] of anionic N‐heterocyclic carbenes with a weakly coordinating borate moiety (WCA‐NHC, WCA=B(C_6_F_5_)_3_, NHC=IDipp=1,3‐bis(2,6‐diisopropylphenyl)imidazolin‐2‐ylidene) were used for the preparation of silver(I) or copper(I) WCA‐NHC complexes. While the reactions in THF with AgCl or CuCl afforded anionic mono‐ and dicarbene complexes with solvated lithium counterions [Li(THF)_n_]^+^ (n=3, 4), the reactions in toluene proceeded with elimination of LiCl and formation of the neutral phosphine and arene complexes [(WCA‐NHC)M(PPh_3_)] and [(WCA‐NHC)M(*η* ^2^‐toluene)] (M=Ag, Cu). The latter were used for the preparation of chlorido‐ and iodido‐bridged heterobimetallic Ag/Ru and Cu/Ru complexes [(WCA‐NHC)M(*μ*‐X)_2_Ru(PPh_3_)(*η* ^6^‐*p*‐cymene)] (M=Ag, Cu, X=Cl; M=Ag, X=I). Surprisingly, these complexes resisted the elimination of CuCl, AgCl, or AgI, precluding WCA‐NHC transmetalation.

## Graphical abstract

**Silver(I) and copper(I) arene complexes** [(WCA‐NHC)M(*η* ^2^‐toluene)] (M=Ag, Cu) with a weakly coordinating anionic (WCA) borate moiety were prepared and their ability to serve as transmetalation reagents was investigated. However, the high stability of WCA‐NHC‐metal bonds provided access to heterobimetallic complexes such as [(WCA‐NHC)M(*μ*‐Cl)_2_Ru(PPh_3_)(*η* ^6^‐*p*‐cymene)], which resisted the elimination of AgCl or CuCl, respectively.

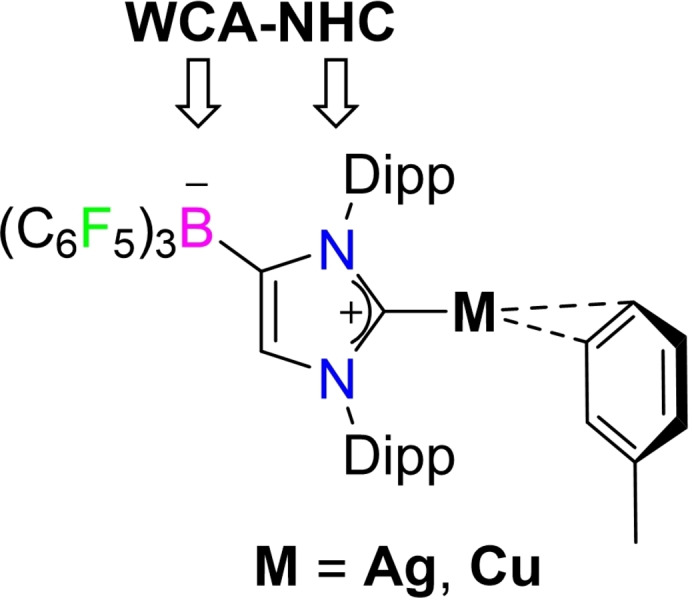

## Introduction

**Figure 1:**
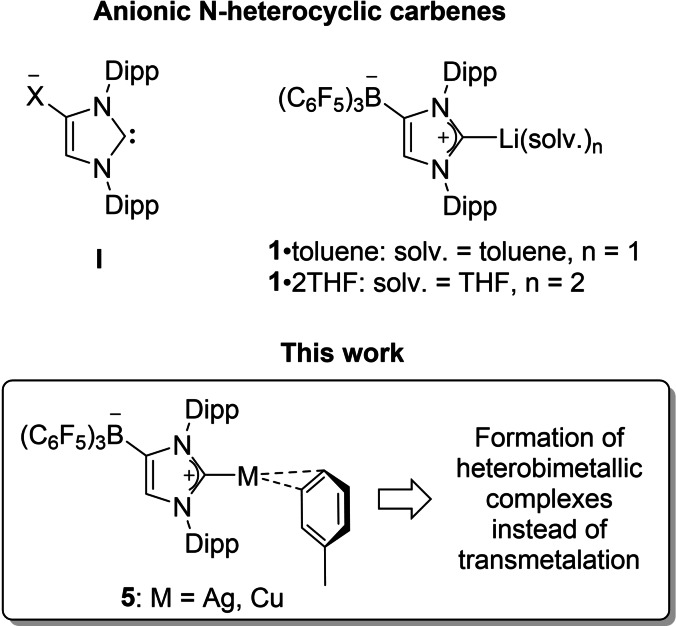
Anionic N‐heterocyclic carbenes and potential transmetalation reagents. It should be noted that formal charges are shown here to illustrate the anionic nature of the N‐heterocyclic carbene ligands; in the following, however, formal charges will be omitted to avoid the assignment of awkward charges in transition metal complexes.

Anionic derivatives of N‐heterocyclic carbenes (NHCs) have become an important subclass of these ubiquitous and indispensable carbon‐donor ligands,^[1]^ with C−H metalation and backbone functionalization of imidazolin‐2‐ylidenes being arguably the most important strategy for the preparation of ditopic carbenes of type **I** (Figure 1),^[2]^ for example, with X=BEt_3_, AlMe_3_,^[3]^ CO_2_,^[4]^ ZnR_2_ (R=Et, *t*Bu),^[5]^ M{N(SiMe_3_)}_2_ (M=Zn, Ge, Sn, Pb).^[6]^ Accordingly, most of these species were isolated as lithium salts by deprotonation of 1,3‐bis(2,6‐diisopropylphenyl)‐imidazolin‐2‐ylidene (IDipp) with *n*‐BuLi, followed by treatment of the intermediate “viable anionic N‐heterocyclic dicarbene”^[3]^ with the respective electrophile. Thus, addition of fluoroboranes afforded so‐called WCA‐NHC systems with a weakly coordinating anionic (WCA) borate moiety such as X=B(C_6_F_5_)_3_, and the resulting solvated lithium salts [(WCA‐NHC)Li(solv.)_n_] (**1**) were used as transmetalation reagents for the preparation of late transition metal complexes, for example, Au(I),^[7]^ Rh(I),^[8]^ Ir(I),[^8^, ^9^] Ni(II),^[10]^ Pd(II),^[11]^ which were preferably used as homogeneous catalysts for applications in nonpolar solvents. Early transition metal cyclopentadienyl‐titanium(IV) and imido‐vanadium(V) complexes were also prepared and used as pre‐catalysts for ethylene copolymerization.^[12]^ Related anionic analogues of NHCs in which the borate unit is attached to the heterocycle via a methylene (CH_2_) spacer were also used in transition metal chemistry.^[13]^ Lately, the lithium salts **1** were employed for the preparation of WCA‐NHC complexes of the heavier p‐block elements, covering groups 13,^[14]^ 15,^[15]^ 16,^[16]^ and 17.^[17]^

In search for alternative transmetalation reagents, sodium and potassium salts of WCA‐NHCs were recently introduced, however, their synthesis through deprotonation of IDipp with Schlosser base combinations of sodium or potassium bis‐(trimethylsilyl)amides and *n*‐BuLi leaves room for further optimisation.^[18]^ Therefore, we turned our attention to WCA‐NHC complexes of the lighter coinage metals, since silver(I) and as well as copper(I) NHC complexes have become well‐established and widely used carbene transfer reagents.^[19]^ As a result, we present, among other things, the synthesis and characterization of Ag(I) and Cu(I) complexes such as [(WCA‐NHC)M(*η* ^2^‐toluene)] (**5**, M=Ag, Cu) and their attempted use for the preparation of ruthenium(II) WCA‐NHC complexes (Figure 1). To our surprise, however, it was found that the anticipated transmetalation reactions do not proceed with the elimination and precipitation of silver(I) or copper(I) halides but can be used for the controlled assembly of heterobimetallic Ag/Ru and Cu/Ru complexes.

## Results and Discussion

**Scheme 1:**
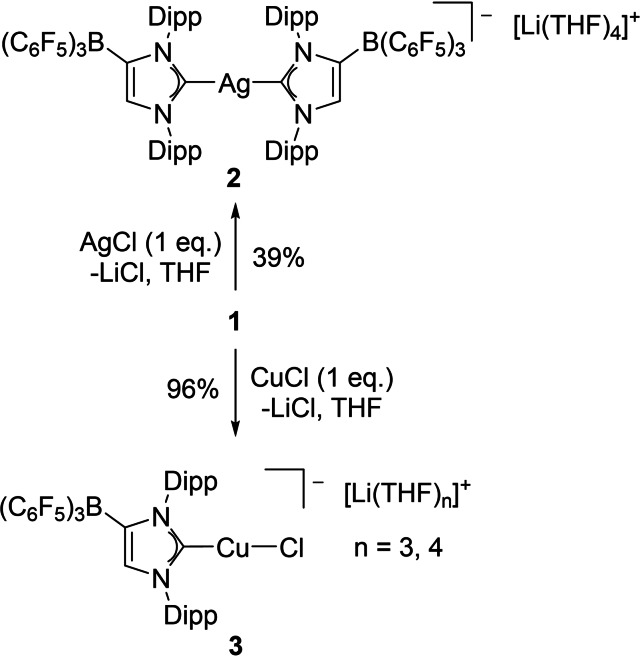
Synthesis of silver(I) and copper(I) WCA‐NHC complexes in THF solution.

**Figure 2:**
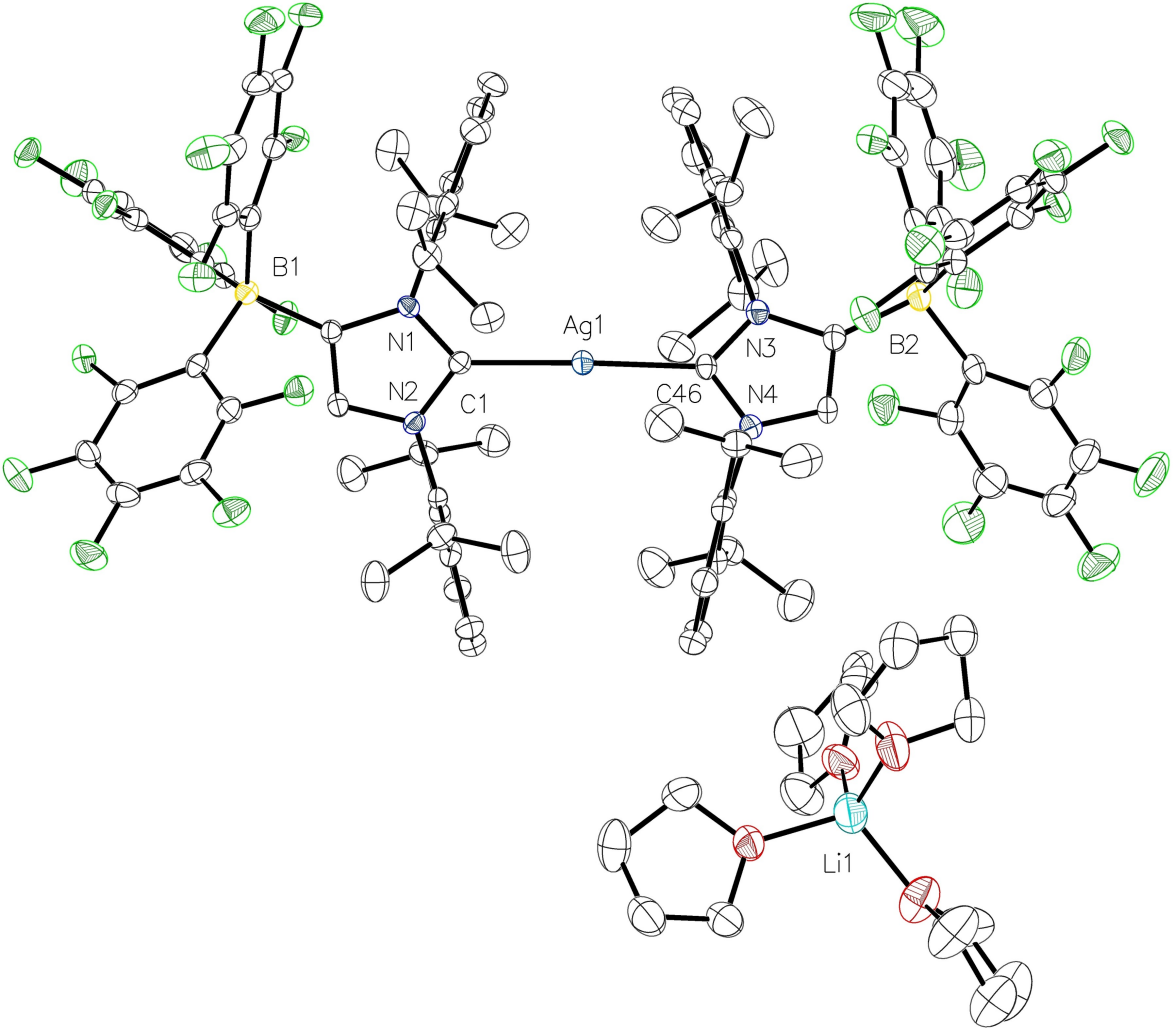
Molecular structure of **2⋅**THF with thermal displacement parameters drawn at 50 % probability. All hydrogen atoms and one molecule THF are omitted for clarity. Pertinent structural data are assembled in Table 1.

We first studied the reaction of the lithium carbene complex **1⋅**toluene with one equivalent of silver(I) and copper(I) chloride in THF solution, which afforded the ionic complexes **2⋅**THF and **3** after filtration through Celite® and recrystallization from THF/*n*‐hexane solution (Scheme 1). Regardless of the 1 : 1 stoichiometry, the reaction with AgCl proceeded with formation of the anionic dicarbene silver complex with a linear C−Ag−C angle of 178.08(10)° and Ag−C bond lengths of 2.141(3) and 2.151(3) Å, which is reminiscent of the structural parameters repeatedly established for the cationic analogues [(IDipp)_2_Ag]^+[20]^ and [(IMes)_2_Ag]^+[21]^ (IMes=1,3‐bis(2,4,6‐trimethylphenyl)imidazolin‐2‐ylidene). The lithium counterion is solvated by four THF molecules and resides in a slightly distorted tetrahedral environment (Figure 2). It is interesting to note that the ^13^C NMR spectrum exhibits a nicely resolved doublet of doublets at 183.9 ppm, with coupling constants ^1^ *J*_C,Ag_ of 195 and 224 Hz for C_carbene_ bonding to the ^107^Ag and ^109^Ag nuclei. These values fall in the range observed for other homoleptic silver(I)‐dicarbene complexes,[^19a^, ^22^] for example, *δ*=183.6 ppm, ^1^ *J*_C,Ag_=188/209 Hz for [(IMes)_2_Ag][CF_3_SO_3_].^[21a]^

**Figure 3:**
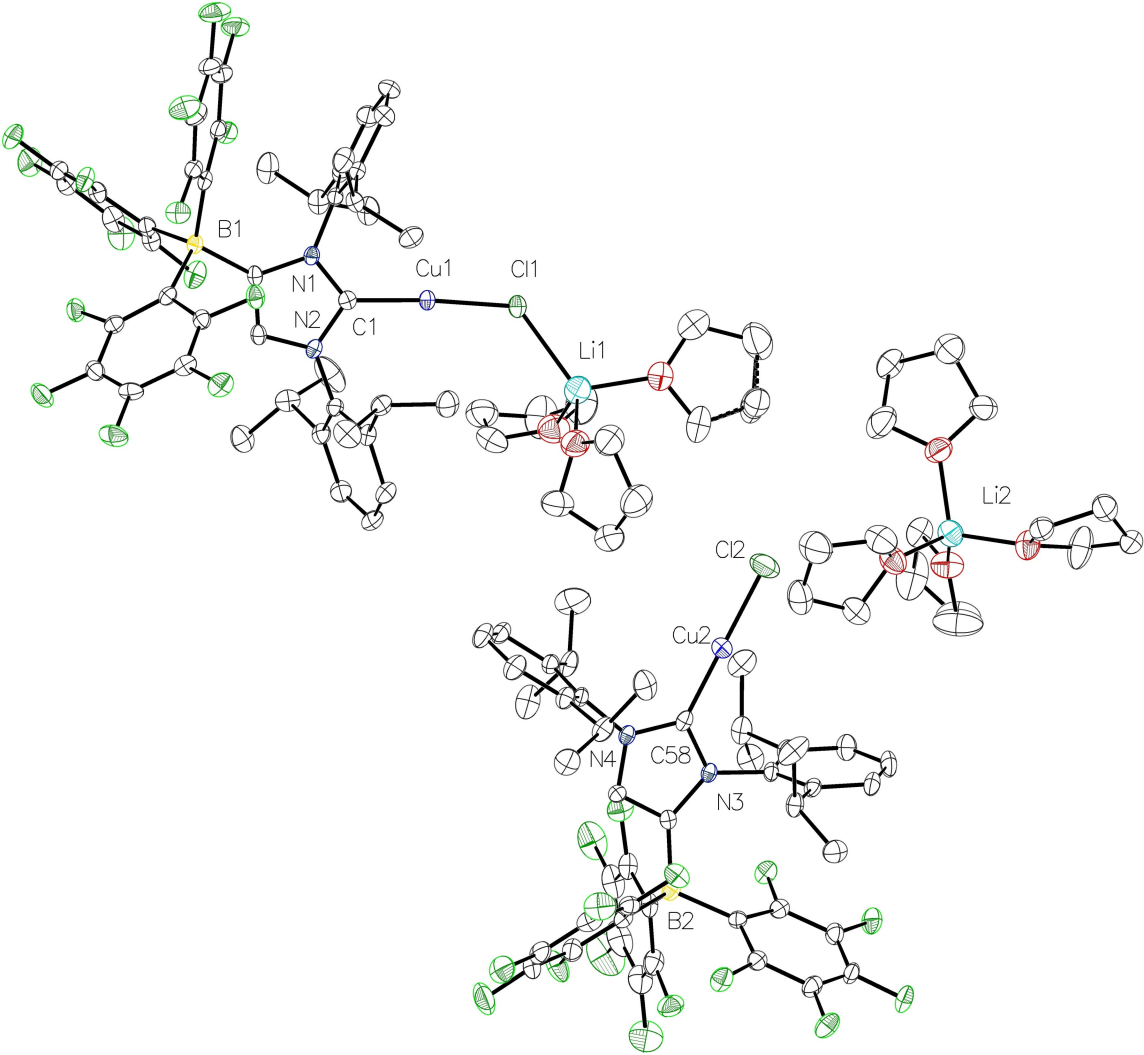
Molecular structure of **3** with thermal displacement parameters drawn at 50 % probability level. The two independent molecules in the asymmetric unit are shown. All hydrogen atoms are omitted for clarity. Pertinent structural data are assembled in Table 1.

The copper complex **3** crystallized with two independent molecular anions [(WCA‐NHC)CuCl]^−^ in the asymmetric unit, together with one [Li(THF)_4_]^+^ and another [Li(THF)_3_]^+^ unit that interacts with one of the chlorido ligands (Figure 3). The copper atoms have linear coordination spheres with C1−Cu1−Cl1=175.59(9)° and C58−Cu–Cl2=179.18(10)°, and the copper‐carbon bond lengths of 1.877(3) Å (Cu1−C1) and 1.879(3) Å (Cu2−C58) are in the same but slightly shorter range compared to the neutral analogues [(IDipp)CuCl]^[23]^ and [(IMes)CuCl].^[24]^ Related copper(I) chloride complexes bearing anionic malonate or enolate functionalized anionic N‐heterocyclic carbenes have also been reported.^[25]^ It should be noted that we also obtained crystals of the complex [Li(THF)_4_][{(WCA‐NHC)Cu}_2_(μ‐Cl)], in which the chlorido ligand is bridging two (WCA‐NHC)Cu moieties; see Figure S11 in the Supporting Information for a presentation of the crystal structure. These findings prompted us to question our synthetic strategy, since carrying out the reactions in THF solution prevented the formation and precipitation of lithium chloride by solvation and resulted in ionic complexes of various, somewhat unreliable compositions. Moreover, transmetalation reactions consistently failed with **2** and **3**, and for instance, the reaction of **3** with [(*η* ^6^‐ *p*‐cymene)RuCl_2_]_2_ afforded a bimetallic salt consisting of the non‐interacting complex ions [{(*η* ^6^‐*p*‐cymene)Ru}_2_(μ‐Cl)_3_]^+^ and [(WCA‐NHC)CuCl]^–^ (see the Supporting Information, Figure S10).

**Scheme 2:**
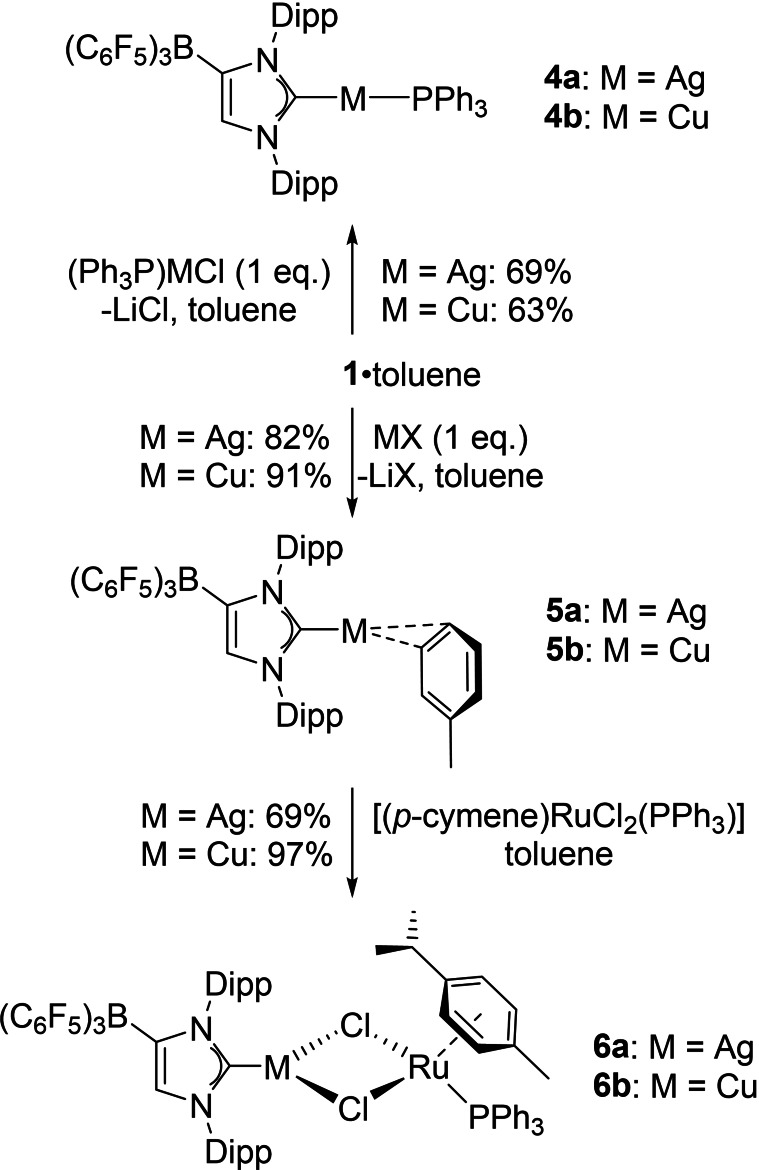
Synthesis of silver(I) and copper(I) WCA‐NHC complexes in toluene solution; synthesis of heterobimetallic Ag/Ru and Cu/Ru complexes; MX=AgOTf or CuCl.

Therefore, we turned our attention to WCA‐NHC transfer reactions in toluene solution, and initial experiments were carried out with chloro(triphenylphosphine)silver(I) and ‐copper(I). Thus, suspending **1** in toluene and addition of [(Ph_3_P)MCl] afforded the complexes [(WCA‐NHC)M(PPh_3_)] (**4**) as colorless (**4** **a**: M=Ag) and yellow (**4** **b**: M=Cu) crystalline solids in 69 % and 63 % yield, respectively, after stirring for ca. 2 h, filtration through Celite® and recrystallization from dichloromethane/*n*‐hexane or THF/*n*‐hexane solutions (Scheme 2). The NMR spectroscopic characteristics are similar to those previously established for the corresponding gold(I) complex [(WCA‐NHC)Au(PPh_3_)], however, the ^31^P NMR resonances are found at significantly higher field, i.e., at 18.3 ppm (**4** **a**) and 8.6 ppm (**4** **b**) in comparison with 40.5 ppm reported for the gold congener.^[7]^ For the silver complex **4** **a**, this signal is observed as a doublet of doublets with ^1^ *J*_P,Ag_=462/533 Hz for phosphorus coupling with the ^107^Ag/^109^Ag nuclei. In contrast, the ^13^C NMR signal for the carbene carbon atom in **4** **a** could not be resolved, whereas **4** **b** gave rise to doublet at 177.2 ppm with ^2^ *J*_C,P_=69 Hz.

**Figure 4:**
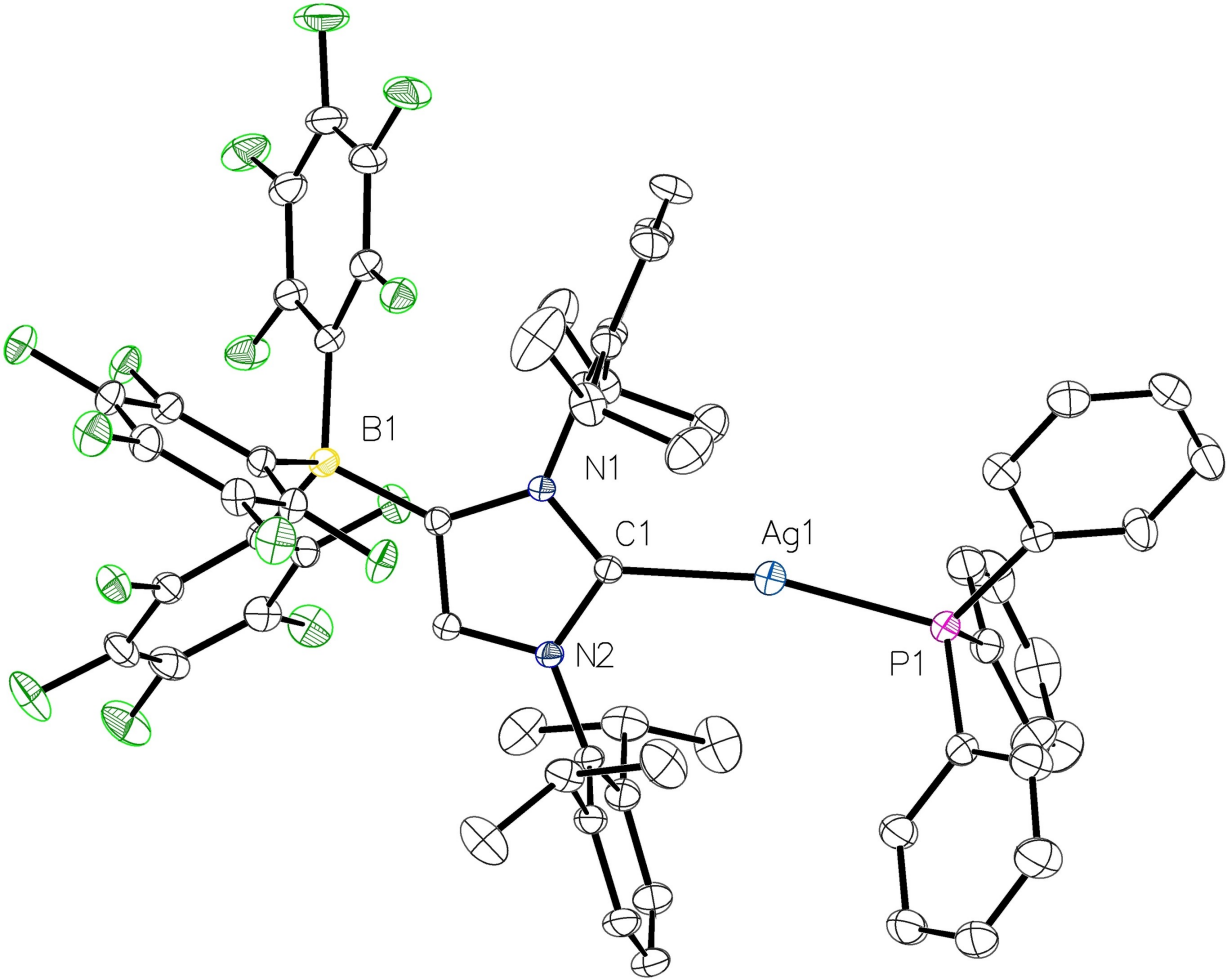
Molecular structure of **4** **a** with thermal displacement parameters drawn at 50 % probability level. All hydrogen atoms are omitted for clarity. Pertinent structural data are assembled in Table 1.

The complexes **4** were further characterized by single‐crystal X‐ray diffraction analysis; they are isotypic and crystallize in the monoclinic space group *P*2_1_/*n*. The molecular structure of the silver complex **4** **a** is shown in Figure 4, whereas the molecular structure of the copper complex **4** **b** is presented in the Supporting Information (Figure S4). Selected bond lengths and angles are summarized in Table 1. The two‐coordinate Ag and Cu atoms display distorted linear environments with C1−M−P1 angles of 166.81(6)° (**4** **a**) and 166.23(4)° (**4** **b**). The metal‐carbon and metal‐phosphorus bond lengths of 2.085(2)/2.3434(6) Å (Ag1−C1/Ag1−P1) and 1.9063(12)/2.1926(4) Å (Cu1−C1/Cu1−P1) fall in the expected ranges and are similar to those established for cationic analogues such as [(IDipp)Ag(PCy_3_)][PF_6_]^[20c]^ and [(IDipp)Cu(PPh_3_)][PF_6_].^[26]^

**Table 1:** Selected bond lengths [Å] and angles [°] of compounds **2**–**7**.

Complex	M−C1	M−X	C1−M−X
**2** (M=Ag, X=C)	2.141(3)	2.151(3)	178.08(10)
**3** (M=Cu, X=Cl)^[a]^	1.877(3), 1.879(3)	2.1353(6), 2.1282(6)	175.59(9), 179.18(10)
**4** **a** (M=Ag, X=P)	2.085(2)	2.3434(6)	166.81(6)
**4** **b** (M=Cu, X=P)	1.9063(12)	2.1926(4)	166.23(4)
**5** **a** (M=Ag, X=C)	2.0839(14)	2.3382(17), 2.4056(17)	164.94(7), 160.24(6)
**5** **b** (M=Cu, X=C)	1.9016(17)	2.2013(19), 2.1220(19)	163.53(8), 158.72(8)
**6** **a** (M=Ag, X=Cl)	2.0907(14)	2.5287(4), 2.6727(4)	147.85(4), 131.98(4)
**6** **b** (M=Cu, X=Cl)	1.898(3)	2.2606(9), 2.5025(9)	151.61(10), 123.34(10)
**7** (M=Ag, X=I)	2.139(5)	2.8619(5), 2.8310(5)	132.64(13), 144.92(13)

[a] Two independent molecules in the asymmetric unit.

Encouraged by the successful chloride substitution and carbene transfer from (WCA‐NHC)Li**⋅**toluene (**1⋅**toluene) onto [(Ph_3_P)MCl] (M=Ag, Cu), the preparation of phosphine‐free WCA‐NHC silver(I) and copper(I) complexes was attempted. Hence, the reaction of **1⋅**toluene with silver(I) trifluoromethanesulfonate (AgOTf) afforded the toluene solvate [(WCA‐NHC)Ag(toluene)] (**5** **a**) in 82 % yield as a white crystalline solid after stirring for 10 min, filtration through Celite® and recrystallization from toluene/dichloromethane solution (Scheme 2). Longer reaction times produced significantly lower yields, which could tentatively be ascribed to ligand exchange and formation of ionic dicarbene‐silver complexes as side products.^[27]^ The corresponding copper complex **5** **b** could be isolated from the reaction of **1⋅**toluene with CuCl in toluene solution and was isolated as a colorless crystalline solid in 91 % yield after stirring for 16 h, filtration through Celite® and recrystallization from toluene/diethyl ether solution. Gratifyingly, the ^13^C NMR spectra of both complexes displayed the expected low‐field signals for the carbene carbon atoms at 182.1 ppm (**5** **a**, THF‐*d*_8_) and 175.1 ppm (**5** **b**, CD_2_Cl_2_). For the silver complex **5** **a**, this signal could be resolved as a doublet of doublets with ^1^ *J*_C,Ag_=300/347 Hz for coupling with the ^107^Ag/^109^Ag nuclei. These coupling constants are significantly larger than usually found for silver(I) monocarbene complexes of the type ([(NHC)AgX], for example,[^19a^, ^22^] ^1^ *J*_C,Ag_=253/271 Hz for [(IDipp)AgCl]^[21b]^, revealing a strong carbon‐silver interaction in the solvated [(WCA‐NHC)Ag] complex fragment of **5** **a**.

**Figure 5:**
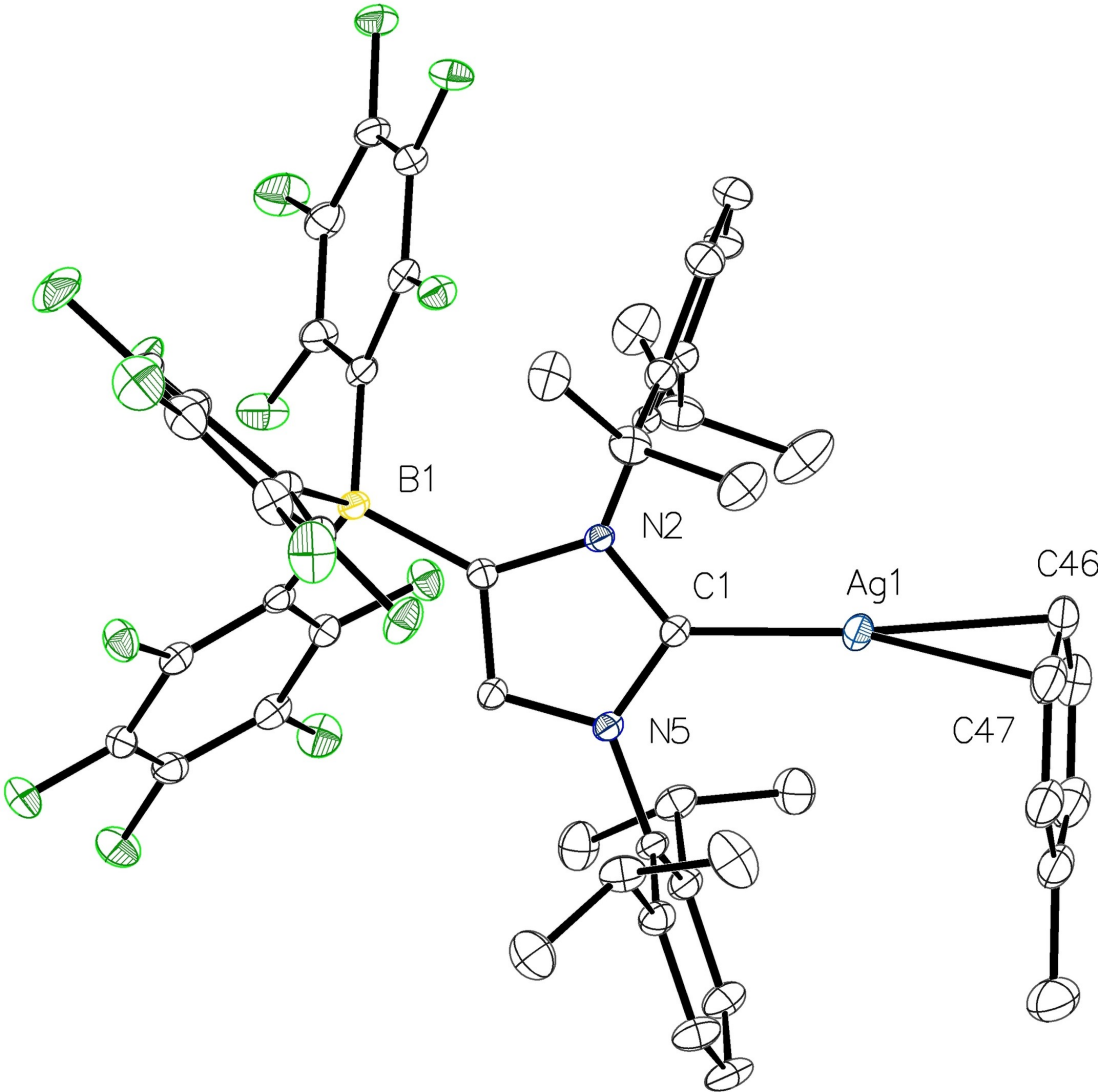
Molecular structure of **5** **a** with thermal displacement parameters drawn at 50 % probability level. All hydrogen atoms are omitted for clarity. Pertinent structural data are assembled in Table 1.

The molecular structures of both complexes **5** could be established by X‐ray diffraction analysis (see Figure 5 for **5** **a** and Figure S6 in the Supporting Information for **5** **b**); they are again isotypic and crystallize in the monoclinic space group *P*2_1_/*c*. The metal‐carbon bond lengths of 2.0839(14) Å (Ag1−C1 in **5** **a**) and 1.9016(17) Å (Cu1−C1 in **5** **b**) are virtually identical with those found in **4** **a** and **4** **b** (Table 1). In addition, both structures confirm the presence of metal‐arene interactions in the solid state, and a toluene ligand is additionally bound to the metal atoms in a *η* ^2^‐fashion with Ag1−C46/C47=2.3382(17)/2.4056(17) Å and Cu1−C46/C47=2.2013(19)/2.1220(19) Å. This interaction is best classified as charge‐transfer bonding, with *η* ^2^‐coordination typically observed for π‐complexes of the late transition metals.^[28]^ Such interactions have been observed only for a small number of cationic silver(I) and copper(I) NHC complexes, which requires the presence of weakly coordinating counterions. Accordingly, the complex [(ITr)Ag(*η* ^2^‐C_6_H_5_F)][BAr^F^_4_] was isolated from a fluorobenzene solution of [(ITr)Ag(OTf)] in the presence of Na[BAr^F^_4_] (ITr=1,3‐bis(triphenylmethyl)‐imidazolin‐2‐ylidene, Ar^F^=3,5‐bis(trifluoromethyl)phenyl). With silver‐carbon distances of 2.115(3), 2.381(4) and 2.435(4) Å, this complex exhibits similar, but slightly longer Ag−C bond lengths compared to **5** **a**.^[29]^ Likewise, the copper(I) complexes [(IDipp)Cu(arene)][SbF_6_] (arene=*η* ^2^‐benzene, *η* ^3^‐C_6_Me_5_, *η* ^3^‐toluene, *η* ^3^‐*m*‐xylene) were isolated by reaction of [(IDipp)CuBr] with AgSbF_6_ in CH_2_Cl_2_/arene solution and feature *η* ^2^‐ or *η* ^3^‐coordination modes in the solid state.^[30]^ Similar metal‐arene interactions were also observed for coinage metal complexes of N‐heterocyclic silylene ligands.^[31]^

Originally, we envisaged that the silver(I) and copper(I) toluene complexes **5** might be ideally suited for WCA‐NHC transfer to transition metals, with the ultimate goal to prepare ruthenium(II) WCA‐NHC complexes for application in olefin metathesis. Such systems could serve as anionic analogues of recently developed and commercialized cationic ruthenium olefin metathesis catalysts bearing ammonium tags.^[32]^ It was found, however, that these complexes resisted the elimination of silver(I) and copper(I) halides, presumably because of their exceptionally strong metal‐carbene bonds. Accordingly, the reactions **5** **a** and **5** **b** with [(*η* ^6^‐*p*‐cymene)RuCl_2_(PPh_3_)] in toluene solution afforded the heterobimetallic complexes **6** as orange‐red crystalline solids in 69 % (**6** **a**) and 97 % (**6** **b**) yield after filtration through Celite® and recrystallization from dichloromethane/*n*‐hexane solutions (Scheme 2). The NMR spectra show the presence of both the (WCA‐NHC)M (M=Ag, Cu) and the (*η* ^6^‐*p*‐cymene)Ru units, with the carbene carbon atoms giving rise to a doublet of doublets at 185.0 ppm with ^1^ *J*_C,Ag_=275/317 Hz and to a singlet at 180.9 ppm in the ^13^C NMR spectra of **6** **a** and **6** **b**, respectively.

**Figure 6:**
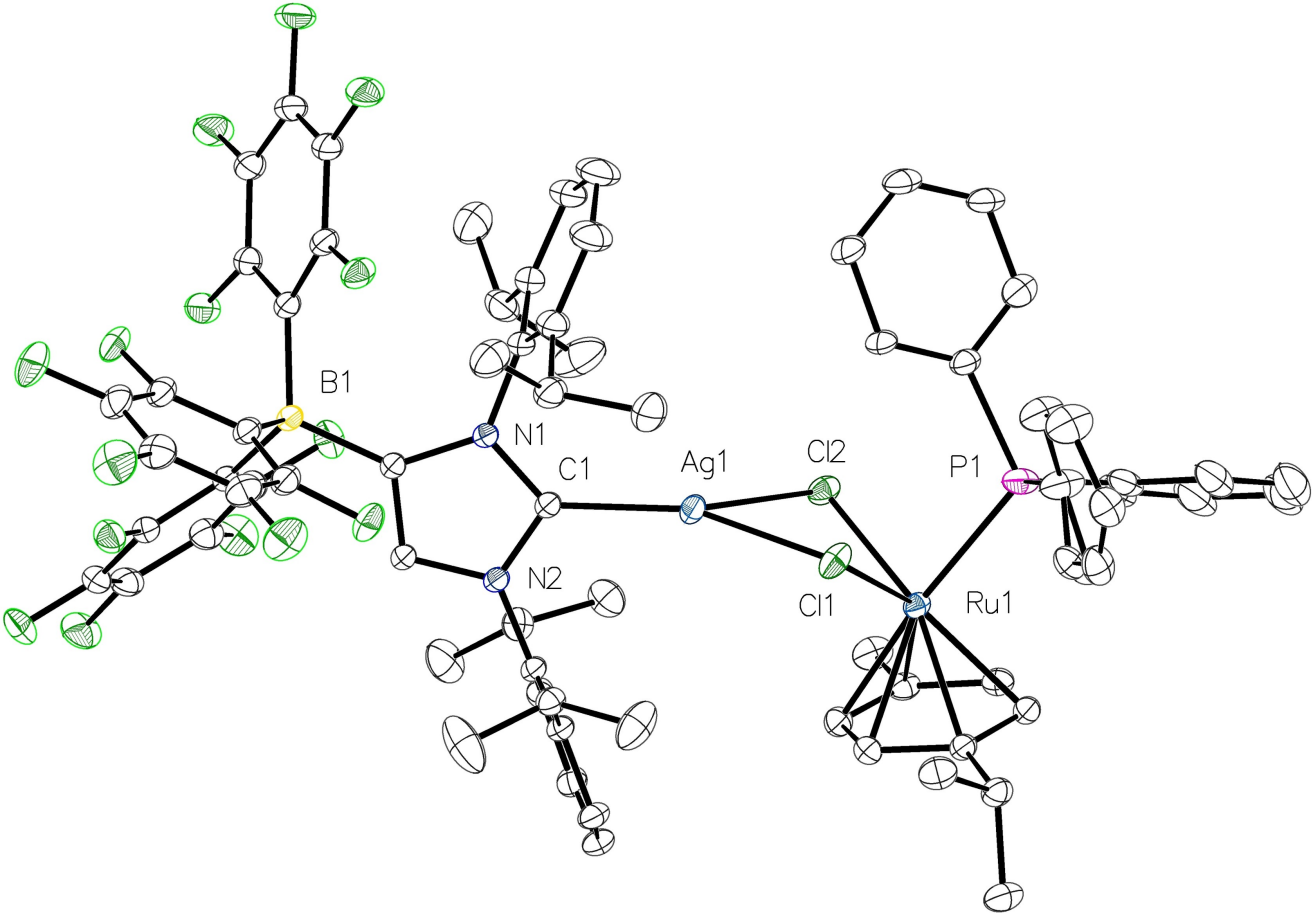
Molecular structure of **6** **a** with thermal displacement parameters drawn at 50 % probability level. All hydrogen atoms and a disordered CH_2_Cl_2_ molecule are omitted for clarity. Two PPh_3_ phenyl rings are disordered over two positions, and only one orientation is shown. Selected bond lengths [Å] and angles [°] in **6** **a**/**6** **b** (M1=Ag1/Cu1): Ru1−Cl1 2.4135(4)/2.4147(9), Ru1−Cl2 2.4156(3)/2.4088(9), Ru1−P1 2.3897(4)/2.3624(9); Cl1−M1−Cl2 78.629(12)/84.27(3), Cl1−Ru1−Cl2 86.129(13)/83.14(3), M1−Cl1−Ru1 93.461(13)/93.03(3), M1−Cl2−Ru1 89.901(12)/87.40(3). Other pertinent structural data are assembled in Table 1.

The heterobimetallic nature of the complexes **6** was confirmed by X‐ray diffraction analysis; **6** **a** crystallized as the solvate **6** **a** ⋅ CH_2_Cl_2_ in the monoclinic space group $P\bar{1}$ , whereas **6** **b** ⋅ CH_2_Cl_2_ crystallized in the orthorhombic space group *Pbca*. The molecular structures are presented in Figure 6 (**6** **a**) and Figure S8 (**6** **b**, see the Supporting Information). The silver and copper atoms have strongly distorted trigonal‐planar coordination spheres and are built into four‐membered M(μ‐Cl)_2_Ru rings (M=Ag, Cu), which are folded along the Cl−Cl diagonal with dihedral angles between the MCl_2_ and RuCl_2_ planes of 35.19(2)° (**6** **a**) and 35.99(5)° (**6** **b**). The WCA‐NHC ligands adopt vertical orientations with respect to the MCl_2_ plane with the B(C_6_F_5_)_3_ and *p*‐cymene units facing in opposite directions. The metal‐carbene bond lengths of 2.0907(14) Å (Ag1−C1) and 1.898(3) Å (Cu1−C1) are almost identical to those of the corresponding complexes **4** and **5** (Table 1). The Ag1−Ru1 and Cu1−Ru1 distances are 3.5995(2) Å (**6** **a**) and 3.3938(6) Å (**6** **b**), ruling out any significant metal‐metal bonding as for instance found in cyclopentadienyl (Cp) ruthenium complexes of the type [(IDipp)MRuCp(CO)_2_] (M=Ag, Ag−Ru=2.607 Å; M=Cu, Cu−Ru=2.439).^[33]^ It should be noted that examples of a controlled construction of halide‐bridged heterobimetallic ruthenium‐silver and ruthenium‐copper complexes are rare, and we are only aware of cyclopentadienone‐ruthenium dicarbonyl complexes containing bridging [(IDipp)MCl] (M=Ag, Cu) units as the only other examples of NHC‐supported heterobimetallic Ru/Ag and Ru/Cu systems.^[34]^

**Scheme 3:**
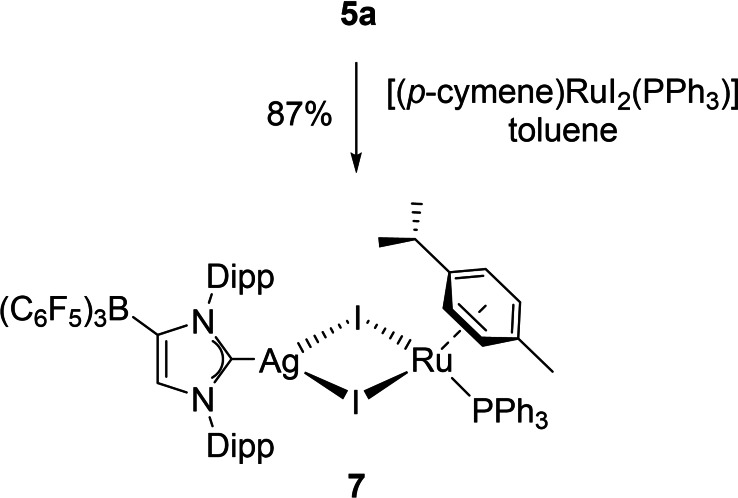
Synthesis of the heterobimetallic Ag/Ru complex **7**.

The resistance of complexes **6** to form a WCA‐NHC‐ruthenium complexes by elimination of AgCl or CuCl prompted us to study the reaction of the silver complex **5** **a** with the corresponding iodine complex [(*η* ^6^‐*p*‐cymene)RuI_2_(PPh_3_)] in dichloromethane, since the formation and precipitation of AgI might favour the formation of a metal‐carbene complex. Surprisingly, however, the heterobimetallic Ag/Ru complex **7** was isolated as a red crystalline solid in 87 % yield after filtration through Celite® and recrystallization from CH_2_Cl_2_/*n*‐hexane solution (Scheme 3). The presence of the (WCA‐NHC)Ag moiety is evident from the ^13^C NMR signal at 182.8 ppm, which appears as the characteristic doublet of doublets with slightly smaller coupling constants of ^1^ *J*_C,Ag_=254/290 Hz compared to the chlorido‐bridged analogue **6** **a**.

**Figure 7:**
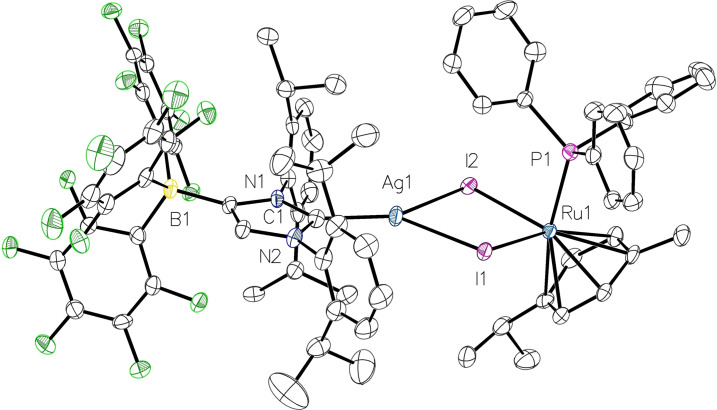
Molecular structure of **7** with thermal displacement parameters drawn at 50 % probability level. All hydrogen atoms are omitted for clarity. Selected bond lengths [Å] and angles [°]: Ru1−I1 2.7263(4), Ru1−I2 2.7236(4), Ru1−P1 2.3677(12); I1−Ag1−I2 82.429(12), I1−Ru1−I2 86.986(12), Ag1−I1−Ru1 94.532(13), Ag1−I2−Ru1 95.299(13). Other pertinent structural data are assembled in Table 1.

The molecular structure of **7** was determined by X‐ray diffraction analysis, confirming the formation of a heterobimetallic Ag/Ru complex with bridging iodido ligands (Figure 7). In contrast to **6** **a**, the four‐membered Ag(μ‐I)_2_Ru ring is close to planarity with a dihedral angle of 8.89(2)° between the AgI_2_ and RuI_2_ planes, which results in a significantly longer Ag1−Ru1 distance of 4.1057(5) Å. In contrast to **6** **a** and **6** **b**, the WCA‐NHC ligand adopts a horizontal conformation and is almost perfectly aligned with the AgI_2_ plane. However, the NMR spectra indicate fast rotation of the WCA‐NHC ligand in solution on the NMR timescale, in agreement with time‐averaged *C*_s_‐symmetry. It should be noted that, to the best of our knowledge, no other crystal structure of an iodido‐bridged Ag/Ru complex has been reported to date.

## Conclusion

With the synthesis and characterization of the complexes [(WCA‐NHC)M(*η* ^2^‐toluene)] (**5**, M=Ag, Cu), we have again successfully exploited this class of anionic N‐heterocyclic carbenes with a weakly coordinating borate moiety (WCA‐NHC) for the generation of neutral analogues of otherwise cationic transition metal complexes for applications in nonpolar solvents.[^7^, ^8^, ^9^] Accordingly, the high solubility of the complexes **5** in toluene and other aromatic hydrocarbons provides easy access to these and potentially numerous other silver(I) and copper(I) π‐arene complexes. Attempts to use the complexes **5** as WCA‐NHC transfer reagents were unsuccessful in the case of ruthenium(II); however, the observed transfer of the intact (WCA‐NHC)M units enabled the isolation of the chlorido‐ and iodido‐bridged heterobimetallic Ag/Ru and Cu/Ru complexes **6** and **7**. While the latter complexes and related systems could find application in cooperative heterobimetallic catalysis,^[35]^ in view of the enormous importance of (NHC)Ag and (NHC)Cu in catalysis,^[36]^ it appears particularly promising to further exploit the potential of the complexes **5** to serve as homogeneous catalysts, especially in nonpolar solvents.

## Experimental Section

All operations with air‐ and moisture‐sensitive compounds were performed in a glove box under a dry argon atmosphere (MBraun 200B) or on a vacuum line using Schlenk techniques. All solvents were distilled from Na/benzophenone or CaH_2_, degassed prior to use and stored over molecular sieves (4 Å). [(WCA‐IDipp)Li(toluene)]^[8]^ and [(*η* ^6^‐*p*‐cymene)RuCl_2_(PPh_3_)]^[37]^ were prepared according to literature procedures. Full details of all analytical methods and experimental procedures can be found in the Supporting Information.

**[Li(THF)_4_][(WCA‐IDipp)_2_Ag] (2)**: A brown vial is charged with [(WCA‐IDipp)Li(toluene)] (**1**, 100 mg, 0.1 mmol, 1 equiv.) and AgCl (14.3 mg, 0.1 mmol, 1 equiv.) dissolved in THF (2 mL). The solution is stirred overnight and then concentrated under high vacuum and layered with *n*‐hexane. After 24 h at −40 °C the supernatant solution is removed, and the crude product washed with *n*‐hexane (2×2 mL). The crude product is then recrystallized from THF/*n*‐hexane to obtain product **2** as colorless crystals (43 mg, 0.019 mmol, 39 %). **Elemental analysis** (%) calc. for: C_106_H_102_AgB_2_F_30_LiN_4_O_4_: C 57.81, H 4.67, N 2.54; found C 58.22, H 5.125, N 2.39. ^**1**^ **H NMR** (400 MHz, THF‐*d*_8_): *δ* [ppm]=7.29 (t, ^3^ *J*_H,H_=7.51 Hz, 2 H, *p*‐Dipp), 7.21 (t, ^3^ *J*_H,H_=7.51 Hz, 2 H, *p*‐Dipp), 7.02 (d, ^3^ *J*_H,H_=7.76 Hz, 4 H, *m*‐Dipp), 6.81 (d, ^3^ *J*_H,H_=7.76 Hz, 4 H, *m*‐Dipp), 6.21 (s, 2 H, CH=CB), 3.64–3.59 (m, 12 H, THF), 2.72 (sept, ^3^ *J*_H,H_=6.76 Hz, 4 H, CH(CH_3_)_2_), 2.44 (sept, ^3^ *J*_H,H_=6.68 Hz, 4 H, CH(CH_3_)_2_), 1.79–1.74 (m, 12 H, THF), 0.88 (d, ^3^ *J*_H,H_=6.76 Hz, 12 H, CH(CH_3_)_2_), 0.79–0.74 (m, 24 H, CH(CH_3_)_2_), 0.72 (d, ^3^ *J*_H,H_=6.92 Hz, 12 H, CH(CH_3_)_2_. ^**11**^ **B{^1^H} NMR** (160 MHz, THF‐*d*_8_): *δ* [ppm]=−15.23 (s). ^**13**^ **C{^1^H} NMR** (125 MHz, THF‐d_*8*_): *δ* [ppm]=183.9 (2×d, ^1^ *J*_C,Ag_=224 Hz, 195 Hz, NCN), 150.5 (m, aryl‐C_6_F_5_ ) 148.6 (m, 2×CB=CH), 146.9 (s, 4×*o*‐Dipp), 146.4 (s, 4×*o*‐Dipp), 140.0 (m, aryl‐C_6_F_5_), 138.5 (s, 2×*i*‐Dipp), 138.1 (m, aryl‐C_6_F_5_), 136.9 (s, 2×*i*‐Dipp),132.7 (s, 2×CH=CB), 130.0 (s, 2×*p*‐Dipp), 129.4 (s, 2×*p*‐Dipp), 124.6 (s, 4×*m*‐Dipp), 124.0 (s, 4×*m*‐Dipp), 68.0 (s, CH_2_‐THF), 28.4 (s, 4×CH(CH_3_)_2_), 28.0 (s, 4×CH(CH_3_)_2_), 27.8 (s, 4×CH(CH_3_)_2_), 26.1 (s, CH_2_‐THF), 24.9 (s, 4×CH(CH_3_)_2_), 23.3 (s, 4×CH(CH_3_)_2_), 21.8 (s, 4×CH(CH_3_)_2_). ^**19**^ **F{^1^H} NMR** (376 MHz, THF‐*d*_8_): *δ* [ppm]=−129.5 (br s, 6 F, *o*‐F), −163.8 (t, *J*=20.3 Hz, 3 F, *p*‐F), −168.2 (t, *J*=18.7 Hz, 6 F, *m*‐F).

**[Li(THF)_n_][(WCA‐IDipp)CuCl] (3)**: A one‐neck flask is charged with [(WCA‐IDipp)Li(toluene)] (**1**, 150 mg, 0.15 mmol, 1.0 equiv.) and dissolved in THF (4 mL). CuCl (15.0 mg, 0.15 mmol, 1.0 equiv.) is added, and the solution is stirred for 2 h and then filtered through a pad of Celite®. The solvent is removed under high vacuum and the crude product recrystallized from THF/*n*‐hexane to obtain product **3** as colorless crystals (186.0 mg, 0.14 mmol, 96 %). **Elemental analysis** (%) calc. for n=3: C_57_H_59_BClCuF_15_LiN_2_O_3_: C 56.35, H 4.90, N 2.31; found C 56.17, H 4.63, N 2.30. ^**1**^ **H NMR** (400 MHz, dichloromethane‐*d*_2_): *δ* [ppm]=7.39 (t, ^3^ *J*_H,H_=7.7 Hz, 1 H, *p*‐Dipp), 7.28 (t, ^3^ *J*_H,H_=7.7 Hz, 1 H, *p*‐Dipp), 7.19 (d, ^3^ *J*_H,H_=7.8 Hz, 2 H, *m*‐Dipp), 6.97 (d, ^3^ *J*_H,H_=7.7 Hz, 2 H, *m*‐Dipp), 6.27 (br s, 1 H, CH=CB), 3.69–3.54 (m, 12 H, THF), 2.85 (sept, ^3^ *J*_H,H_=6.5 Hz, 2 H, CH(CH_3_)_2_), 2.64 (sept, ^3^ *J*_H,H_=6.8 Hz, 2 H, CH(CH_3_)_2_), 1.90–1.78 (m, 11 H, THF), 1.16–1.02 (m, 18 H, CH(CH_3_)_2_), 0.90 (d, ^3^ *J*_H,H_=6.7 Hz, 6 H, CH(CH_3_)_2_). ^**11**^ **B{^1^H} NMR** (128 MHz, dichloromethane‐*d*_2_): *δ* [ppm]=−16.18 (s). ^**13**^ **C{^1^H} NMR** (101 MHz, dichloromethane‐*d*_2_): *δ* [ppm]=177.2 (s, NCN), 150.4 (m, aryl‐C_6_F_5_), 148.0 (m, CH=CB), 147.7 (s, 2×*o*‐Dipp), 146.7 (s, 2×*o*‐Dipp), 140.3 (m, aryl‐C_6_F_5_), 138.2 (m, aryl‐C_6_F_5_), 137.5 (s, 2×*i*‐Dipp), 135.9 (m, aryl‐C_6_F_5_), 135.8 (s, 2×*i*‐Dipp), 130.4 (br s, CH=CB), 130.1 (s, 2×*p*‐Dipp), 129.6 (s, 2×*p*‐Dipp), 124.1 (s, 2×*m*‐Dipp), 123.2 (s, 2×*m*‐Dipp), 68.8 (s, CH_2_‐THF), 28.5 (s, 2×CH(CH_3_)_2_), 28.1 (s, 2×CH(CH_3_)_2_), 27.5 (s, 2×CH(CH_3_)_2_), 25.9 (s, 2×CH(CH_3_)_2_), 25.0 (s, 2×CH(CH_3_)_2_), 24.1 (s, 2×CH(CH_3_)_2_), 22.0 (s, 2×CH(CH_3_)_2_). ^**19**^ **F{^1^H} NMR** (376 MHz, dichloromethane‐*d*_2_): *δ* [ppm]=−129.3 (br s, 6 F, *o*‐F), −162.8 (t, *J*=20.4 Hz, 3 F, *p*‐F), −167.5 (t, *J*=18.5 Hz, 6 F, *m*‐F). **HRMS** (ES−) *m/z* calcd for [(WCA‐IDipp)CuCl]^−^ (C_45_H_35_BCuClF_15_N_2_): 997.1638, found 997.1628.

**[(WCA‐IDipp)Ag(PPh_3_)] (4** **a)**: A brown one‐neck flask is charged with [(WCA‐IDipp)Li(toluene)] (**1**, 150 mg, 0.15 mmol, 1.0 equiv.) and suspended in toluene (8 mL). Chloro(triphenylphosphine)silver(I) (61.0 mg, 0.15 mmol, 1.0 equiv.) is added and the suspension is stirred for 2 h and then filtered through a pad of Celite®. The solvent is removed under high vacuum, and the crude product recrystallized from DCM/*n*‐hexane to obtain product **4** **a** as colorless crystals (130.0 mg, 0.10 mmol, 69 %). **Elemental analysis** (%) calc. for C_63_H_50_AgBF_15_N_2_P: C 59.59, H 3.97, N 2.21; found C 59.71, H 4.00, N 2.14. ^**1**^ **H NMR** (400 MHz, dichloromethane‐*d*_2_): *δ* [ppm]=7.50–7.43 (m, 4 H, PPh_3_), 7.41 (d, ^3^ *J*_H,H_=7.7 Hz, 1 H, *p*‐Dipp) 7.36–7.27 (m, 6 H, PPh_3_+*p*‐Dipp), 7.25 (d, ^3^ *J*_H,H_=7.8 Hz, 2 H, *m*‐Dipp), 7.08 (d, ^3^ *J*_H,H_=7.8 Hz, 2 H, *m*‐Dipp), 6.95–6.85 (m, 6 H, PPh_3_), 6.47 (br s, 1 H), 2.97 (sept, ^3^ *J*_H,H_=6.7 Hz, 2 H, CH(CH_3_)_2_), 2.73 (sept, ^3^ *J*_H,H_=6.5 Hz, 2 H, CH(CH_3_)_2_), 1.14 (d, ^3^ *J*_H,H_=6.8 Hz, 6 H, CH(CH_3_)_2_), 1.09 (m, 12 H, CH(CH_3_)_2_), 0.93 (d, ^3^ *J*_H,H_=6.7 Hz, 6 H, CH(CH_3_)_2_). ^**11**^ **B{^1^H} NMR** (128 MHz, dichloromethane‐*d*_2_): *δ* [ppm]=−16.12 (s). ^**13**^ **C{^1^H} NMR** (101 MHz, dichloromethane‐*d*_2_): *δ* [ppm]=150.5 (m, aryl‐C_6_F_5_), 148.2 (m, CH=CB), 147.9 (s, 2×*o*‐Dipp), 146.9 (s, 2×*o*‐Dipp), 140.4 (m, aryl‐C_6_F_5_), 138.4 (m, aryl‐C_6_F_5_), 137.9 (s, 2×*i*‐Dipp), 136.0 (m, aryl‐C_6_F_5_), 135.7 (s, 2×*i*‐Dipp), 134.1 (dd, ^2^ *J*_C,P_=15.9, *J*_C,Ag_=2.6 Hz, *o*‐PPh_3_), 132.1 (d, ^4^ *J*_C,P_=2.2 Hz, *p*‐PPh_3_), 130.8 (br s, CH=CB), 130.5 (s, 2×*p*‐Dipp), 130.0 (s, 2×*p*‐Dipp), 129.9 (d, ^3^ *J*_C,P_=10.9 Hz, *m*‐PPh_3_), 129.0 (dd, ^1^ *J*_C,P_=42.3, *J*_C,Ag_=3.3 Hz, *i*‐PPh_3_), 124.4 (s, 2×*m*‐Dipp), 123.7 (s, 2×*m*‐Dipp), 28.6 (s, 2×CH(CH_3_)_2_), 28.2 (s, 2×CH(CH_3_)_2_), 28.0 (s, 2×CH(CH_3_)_2_), 25.1 (s, 2×CH(CH_3_)_2_), 24.4 (s, 2×CH(CH_3_)_2_), 22.1 (s, 2×CH(CH_3_)_2_). ^**19**^ **F{^1^H} NMR** (376 MHz, dichloromethane‐*d*_2_): *δ* [ppm]=−129.5 (br s, 6 F, *o*‐F), −162.4 (t, *J*=20.3 Hz, 3 F, *p*‐F), −167.3 (t, *J*=18.6 Hz, 6 F, *m*‐F). ^**31**^ **P{^1^H} NMR** (162 MHz, dichloromethane‐*d*_2_): *δ* [ppm]=18.3 (dd, ^1^ *J*_P,Ag_=532.9, 461.7 Hz). **HRMS** (ES−) *m/z* calcd for [(WCA‐IDipp)AgCl]^−^ (C_45_H_35_BAgClF_15_N_2_): 1041.1393, found 1041.1398.

**[(WCA‐IDipp)Cu(PPh_3_)] (4** **b)**: A Schlenk flask is charged with chloro(triphenylphosphine)copper(I) (36.17 mg, 0.1 mmol, 1 equiv.) and [(WCA‐IDipp)Li(toluene)] (**1**, 100.0 mg, 0.1 mmol, 1 equiv.) dissolved in chlorobenzene (5 mL) under exclusion of light. The mixture is stirred at room temperature for 2.5 h and then filtered through a pad of Celite®. The solvent removed under high vacuum, and the yellowish solid recrystallized from THF/DCM layered with *n*‐hexane to obtain product **4** **b** as yellow crystals (77.0 mg, 0.063 mmol, 63 %). **Elemental analysis** (%) calc. for. C_63_H_50_BCuF_15_N_2_P: C 61.75, H 4.11, N, 2.29; found C 62.09, H 4.375, N 2.31. ^**1**^ **H NMR** (500 MHz, THF‐*d*_8_): *δ* [ppm]=7.46 (m, 5 H, PPh_3_), 7.32 (m, 8 H, PPh_3_+aryl‐Dipp), 7.16 (d, ^3^ *J*_H,H_=6.80 Hz, 2 H, *m*‐Dipp), 6.87 (m, 6 H, PPh_3_+aryl‐Dipp), 6.67 (s, 1 H, CH=CB), 3.04 (sept, ^3^ *J*_H,H_=6.80 Hz, 2 H, CH(CH_3_)_2_), 2.78 (sept, ^3^ *J*_H,H_=6.86 Hz, 2 H, CH(CH_3_)_2_), 1.13 (d, ^3^ *J*_H,H_=6.85 Hz, 6 H, CH(CH_3_)_2_), 1.08 (m, 12 H, CH(CH_3_)_2_), 0.96 (d, ^3^ *J*_H,H_=6.83 Hz, 6 H, CH(CH_3_)_2_). ^**11**^ **B{^1^H} NMR** (160 MHz, THF‐*d*_8_): *δ* [ppm]=−15.58 (s). ^**13**^ **C{^1^H} NMR** (125 MHz, THF‐*d*_8_): *δ* [ppm]=177.2 (d, ^2^ *J*_C,P_=68.6 Hz, NCN), 150.5 (m, aryl‐C_6_F_5_), 148.6 (m, aryl‐C_6_F_5_+CH=CB), 148.1 (s, 2×*o*‐Dipp), 147.0 (s, 2×*o*‐Dipp), 140.3 (m, aryl‐C_6_F_5_), 138.4 (m, aryl‐C_6_F_5_), 137.8 (s, *i*‐Dipp), 136.3 (m, aryl‐C_6_F_5_), 135.7 (s, *i*‐Dipp), 134.2 (d, ^2^ *J*_C,P_=14.8 Hz, *o‐*PPh_3_), 132.2 (s, *p*‐PPh_3_), 131.2 (br s, CH=CB), 130.5 (d, ^1^ *J*_C,P_=42.4 Hz, *i‐*PPh_3_), 130.0 (d ^3^ *J*_C,P_=10.6 Hz, *m*‐PPh_3_), 129.1 (s, *p‐*Dipp), 128.7 (s, *p‐*Dipp), 124.5 (s, 2×*m*‐Dipp), 123.8 (s, 2×*m*‐Dipp), 28.8 (s, 2×CH(CH_3_)_2_), 28.4 (s, 2×CH(CH_3_)_2_), 28.3 (s, 2×CH(CH_3_)_2_), 24.7 (s, 2×CH(CH_3_)_2_), 24.4 (s, 2×CH(CH_3_)_2_), 21.6 (s, 2×CH(CH_3_)_2_). ^**19**^ **F{^1^H} NMR** (470 MHz, THF‐*d*_8_): *δ* [ppm]=−128.5 (br s, 6 F, *o‐*F), −162.6 (m, 3 F, *p*‐F), −167.0 (s, 6 F, *m*‐F). ^**31**^ **P{^1^H} NMR** (202 MHz, THF‐*d*_8_): *δ* [ppm]=8.6 (s).

**[(WCA‐IDipp)Ag(*η* ^2^‐toluene)] (5** **a)**: A brown one‐neck flask is charged with [(WCA‐IDipp)Li(toluene)] (**1**, 100 mg, 0.1 mmol, 1.0 equiv.) and suspended in toluene (5 mL). Silver(I) trifluoromethanesulfonate (25.7 mg, 0.1 mmol, 1.0 equiv.) is added and the suspension is stirred for 10 minutes and then filtered through a pad of Celite®. The solvent is removed under high vacuum and the crude product recrystallized from toluene/DCM to obtain product **5** **a** as colorless crystals (90.0 mg, 0.08 mmol, 82 %). **Elemental analysis** (%) calc. for C_52_H_43_AgBF_15_N_2_ ⋅ 1/2 C_7_H_8_: C 58.19, H 4.14, N 2.45; found C 58.06, H 3.76, N 2.42. ^**1**^ **H** **NMR** (400 MHz, THF‐*d_8_*): *δ* [ppm]=7.44–7.38 (t, ^3^ *J*_H,H_=8.0 Hz, 1 H, *p*‐Dipp), 7.32 (t, ^3^ *J*_H,H_=7.8 Hz, 2 H, *p*‐Dipp) 7.28 (d, ^3^ *J*_H,H_=7.7 Hz, 2 H, *m*‐Dipp), 7.21–7.17 (m, 2 H, Toluene), 7.16–7.04 (m, 5 H, Toluene+*m*‐Dipp), 6.58 (br s, 1 H, CH=CB), 3.03 (sept, ^3^ *J*_H,H_=6.8 Hz, 2 H, CH(CH_3_)_2_), 2.77 (sept, ^3^ *J*_H,H_=6.9 Hz, 2 H, CH(CH_3_)_2_), 2.31 (s, 4 H, Toluene), 1.22 (dd, ^3^ *J*_H,H_=9.1, 6.8 Hz, 12 H, CH(CH_3_)_2_), 1.14 (d, ^3^ *J*_H,H_=6.8 Hz, 6 H, CH(CH_3_)_2_), 0.97 (d, ^3^ *J*_H,H_=6.7 Hz, 6 H, CH(CH_3_)_2_). ^**11**^ **B{^1^H}** **NMR** (128 MHz, dichloromethane‐*d_2_*): *δ* [ppm]=−16.04 (s). ^**13**^ **C{^1^H}** **NMR** (101 MHz, THF‐*d_8_*): *δ* [ppm]=182.1 (dd, ^2^ *J*_C,Ag_=299.9, 347.4 Hz, NCN), 151.2 (m, aryl‐C_6_F_5_), 148.8 (m, 2×CB=CH), 148.4 (s, 2×*o*‐Dipp), 147.4 (s, 2×*o*‐Dipp), 140.9 (m, aryl‐C_6_F_5_), 139.5 (s, *i*‐Dipp), 138.7 (m, aryl‐C_6_F_5_), 138.6 (s, C_1_‐Tol), 137.5 (s, *i*‐Dipp), 136.4 (m, aryl‐C_6_F_5_), 131.5 (br s, CH=CB), 130.6 (s, *p‐*Dipp), 130.2 (s, *p‐*Dipp), 129.8 (s, C_2,6_‐Tol), 129.1 (s, C_3,5_‐Tol), 126.2 (s, C_4_‐Tol), 124.8 (s, *m‐*Dipp), 124.1 (s, *m‐*Dipp), 29.0 (s, 2×CH(CH_3_)_2_), 28.6 (s, 2×CH(CH_3_)_2_), 27.8 (s, 2×CH(CH_3_)_2_), 25.1 (s, 2×CH(CH_3_)_2_), 24.4 (s, 2×CH(CH_3_)_2_), 22.3 (s, 2×CH(CH_3_)_2_), 21.6 (CH_3_‐Tol). ^**19**^ **F{^1^H}** **NMR** (376 MHz, THF‐*d_8_*): *δ* [ppm]=−129.5 (br s, 6 F, *o*‐F), −163.8 (t, *J*=20.3 Hz, 3 F, *p*‐F), −168.2 (t, *J*=18.7 Hz, 6 F, *m*‐F). **HRMS** (ES−) *m/z* calcd for [(WCA‐IDipp)AgCl]^−^ (C_45_H_35_BAgClF_15_N_2_): 1041.1393, found 1041.1385.

**[(WCA‐IDipp)Cu(*η* ^2^‐toluene)] (5** **b)**: A one‐neck flask is charged with [(WCA‐IDipp)Li(toluene)] (**1**, 120 mg, 0.12 mmol, 1.0 equiv.) and suspended in toluene (5 mL). CuCl (24.0 mg, 0.24 mmol, 2.0 equiv.) is added and the suspension is stirred for 16 h and then filtered through a pad of Celite®. The solvent is removed under high vacuum and the crude product recrystallized from toluene/diethyl ether to obtain product **5** **b** as colorless crystals (115.0 mg, 0.11 mmol, 91 %). **Elemental analysis** (%) calc. for C_52_H_43_BCuF_15_N_2_⋅C_7_H_8_: C 61.76, H 4.48, N 2.44; found C 61.74, H 4.42, N 2.67. ^**1**^ **H NMR** (400 MHz, dichloromethane‐*d*_2_): *δ* [ppm]=7.47 (t, ^3^ *J*_H,H_=7.8 Hz, 1 H, *p*‐Dipp), 7.41 (t, ^3^ *J*_H,H_=7.8 Hz, 1 H, *p*‐Dipp), 7.26 (d, ^3^ *J*_H,H_=7.8 Hz, 2 H, *m*‐Dipp), 7.10 (d, *J*=7.7 Hz, 2 H, *m*‐Dipp), 7.04–7.00 (m, 5 H, Toluene), 6.89–6.80 (m, 1 H, Toluene), 6.30 (br s, 1 H, CH=CB), 2.75 (sept, ^3^ *J*_H,H_=6.8 Hz, 2 H, CH(CH_3_)_2_), 2.41 (sept, ^3^ *J*_H,H_=7.0 Hz, 2 H, CH(CH_3_)_2_), 2.12 (s, 5 H, Toluene), 1.18 (d, ^3^ *J*_H,H_=6.9 Hz, 6 H, CH(CH_3_)_2_), 1.08 (d, ^3^ *J*_H,H_=6.8 Hz, 6 H, CH(CH_3_)_2_), 1.04 (d, ^3^ *J*_H,H_=6.8 Hz, 6 H, CH(CH_3_)_2_), 0.88 (d, ^3^ *J*_H,H_=6.7 Hz, 6 H, CH(CH_3_)_2_). ^**11**^ **B{^1^H} NMR** (128 MHz, dichloromethane‐*d*_2_): *δ* [ppm]=−16.21 (s). ^**13**^ **C{^1^H} NMR** (101 MHz, dichloromethane‐*d*_2_): *δ* [ppm] =175.1 (s, NCN), 150.4 (m, aryl‐C_6_F_5_), 148.0 (s, CH=CB), 147.4 (s, 2×*o*‐Dipp), 146.5 (s, 2×*o*‐Dipp), 140.4 (m, aryl‐C_6_F_5_), 139.5 (s, *i*‐Dipp), 138.3 (m, aryl‐C_6_F_5_), 138.1 (s, C_1_‐Tol), 135.9 (m, aryl‐C_6_F_5_), 135.4 (s, *i*‐Dipp), 130.8 (CH=CB), 130.5 (s, *p‐*Dipp), 130.0 (s, *p‐*Dipp), 127.5 (s, C_2,6_‐Tol), 125.1 (br s, C_3,5_‐Tol), 124.2 (s, *m‐*Dipp), 123.6 (s, *m‐*Dipp), 121.3 (s, C_4_‐Tol), 28.5 (s, 2×CH(CH_3_)_2_), 28.1 (s, 2×CH(CH_3_)_2_), 27.7 (s, 2×CH(CH_3_)_2_), 24.9 (s, 2×CH(CH_3_)_2_), 24.4 (s, 2×CH(CH_3_)_2_), 22.1 (s, 2×CH(CH_3_)_2_), 21.6 (CH_3_‐Tol). ^**19**^ **F{^1^H} NMR** (376 MHz, dichloromethane‐*d*_2_): *δ* [ppm]=−129.6 (br s, 6 F, *o*‐F), −163.4 (t, *J*=20.4 Hz, 3 F, *p*‐F), −167.3 (t, *J*=18.5 Hz, 6 F, *m*‐F). **HRMS** (ES−) *m/z* calcd for [(WCA‐IDipp)CuCl]^−^ (C_45_H_35_BCuClF_15_N_2_): 997.1638, found 997.1624.

**[(WCA‐IDipp)Ag(*μ*‐Cl_2_)Ru(PPh_3_)(*η* ^6^‐*p‐*cymene)] (6** **a)**: A brown one‐neck flask is charged with [(WCA‐IDipp)Ag(toluene)] (**5** **a**, 55.0 mg, 0.05 mmol, 1.0 equiv.) and suspended in toluene (5 mL). [(*η* ^6^‐*p*‐cymene)RuCl_2_(PPh_3_)] (28.4 mg, 0.05 mmol, 1.0 equiv.) is added and the mixture is stirred for 1 h and then filtered through a pad of Celite®. The solvent is removed under high vacuum and the crude product recrystallized at −37 °C from DCM/*n*‐hexane to obtain product **6** **a** as orange/red crystals (54.0 mg, 0.034 mmol, 69 %). **Elemental analysis** (%) calc. for: C_73_H_64_AgBCl_2_F_15_N_2_PRu⋅CH_2_Cl_2_: C 53.72, H 4.21, N 1.67; found C 53.98, H 3.78, N 1.73. ^**1**^ **H NMR** (400 MHz, dichloromethane‐*d*_2_): *δ* [ppm]=7.55–7.34 (m, 11 H, PPh_3_+*p*‐Dipp), 7.33–7.21 (m, 8 H, PPh_3_+*m*‐Dipp), 7.10 (d, ^3^ *J*_H,H_=7.7 Hz, 2 H, *m*‐Dipp), 6.39 (d, *J*_H,F_=2.3 Hz, 1 H), 4.83 (d, ^3^ *J*_H,H_=6.0 Hz, 2 H, CH‐Cymene), 4.71 (d, ^3^ *J*_H,H_=6.1 Hz, 2 H, CH‐Cymene), 2.94 (sept, ^3^ *J*_H,H_=6.8 Hz, 2 H, CH(CH_3_)_2_‐Dipp), 2.73 (sept, ^3^ *J*_H,H_=6.6 Hz, 2 H, CH(CH_3_)_2_‐Dipp), 2.28 (sept, ^3^ *J*_H,H_=6.8 Hz, 1 H, CH(CH_3_)_2_‐Cymene), 1.83 (s, 3 H), 1.29 (d, ^3^ *J*_H,H_=6.9 Hz, 6 H, CH(CH_3_)_2_), 1.20 (d, ^3^ *J*_H,H_=6.8 Hz, 6 H, CH(CH_3_)_2_), 1.12 (d, ^3^ *J*_H,H_=6.8 Hz, 6 H, CH(CH_3_)_2_), 0.98 (d, ^3^ *J*_H,H_=6.9 Hz, 6 H, CH(CH_3_)_2_), 0.91 (d, ^3^ *J*_H,H_=6.7 Hz, 6 H, CH(CH_3_)_2_). ^**11**^ **B{^1^H} NMR** (128 MHz, dichloromethane‐*d*_2_): *δ* [ppm]=−16.02 (s). ^**13**^ **C{^1^H} NMR** (101 MHz, dichloromethane‐*d*_2_): *δ* [ppm]=185.0 (dd, ^1^ *J*_C,Ag_=317.1, 274.7 Hz, NCN) 150.5 (m, aryl‐C_6_F_5_), 148.2 (m, CH=CB), 148.0 (s, 2×*o*‐Dipp), 147.4 (s, 2×*o*‐Dipp), 140.3 (m, aryl‐C_6_F_5_), 138.9 (s, 2×*i*‐Dipp), 138.1 (m, aryl‐C_6_F_5_), 136.9 (s, 2×*i*‐Dipp), 135.8 (m, aryl‐C_6_F_5_), 134.2 (d, ^2^ *J*_C,P_=9.6 Hz, *o*‐PPh_3_), 132.8 (d, ^1^ *J*_C,P_=46.7 Hz, *i*‐PPh_3_), 131.4 (d, ^4^ *J*_C,P_=2.2 Hz, *p*‐PPh_3_), 130.4 (br s, CH=CB), 129.9 (s, 2×*p*‐Dipp), 129.3 (s, 2×*p*‐Dipp), 128.8 (d, ^3^ *J*_C,P_=10.1 Hz, *m*‐PPh_3_), 124.0 (s, 2×*m*‐Dipp), 123.3 (s, 2×*m*‐Dipp), 109.4 (s, C_Ar_‐Cymene), 97.3 (s, C_Ar_‐Cymene), 90.9 (d, ^2^ *J*_C,P_=4.0 Hz, CH_Ar_‐Cymene), 86.7 (d, ^2^ *J*_CP_=5.1 Hz, CH_Ar_‐Cymene), 31.1 (s, CH(CH_3_)_2_‐Cymene), 28.6 (s, 2×CH(CH_3_)_2_‐Dipp), 28.2 (s, 2×CH(CH_3_)_2_‐Dipp), 27.6 (s, 2×CH(CH_3_)_2_‐Dipp), 24.8 (s, 2×CH(CH_3_)_2_‐Dipp), 24.7 (s, 2×CH(CH_3_)_2_‐Dipp), 22.4 (s, 2×CH(CH_3_)_2_‐Cymene), 22.4 (s, 2×CH(CH_3_)_2_‐Dipp), 18.4 (s, CH_3_‐Cymene). ^**19**^ **F{^1^H} NMR** (376 MHz, dichloromethane‐*d*_2_): *δ* [ppm]=−129.7 (br s, 6 F, *o*‐F), −162.8 (t, *J*=20.4 Hz, 3 F, *p*‐F), −167.5 (t, *J*=18.5 Hz, 6 F, *m*‐F). ^**31**^ **P{^1^H} NMR** (162 MHz, dichloromethane‐*d*_2_): *δ* [ppm]=25.6 (s). **HRMS** (ES−) *m/z* calcd for [(WCA‐IDipp)AgCl]^−^ (C_45_H_35_BN_2_F_15_ClAg): 1041.1393, found 1041.1379. **HRMS** (ES+) *m/z* calcd for [(*p*‐cymene)Ru(PPh_3_)]^+^ (C_28_H_28_PRu): 497.0972, found 497.0971.

**[(WCA‐IDipp)Cu(*μ*‐Cl_2_)Ru(PPh_3_)(*η* ^6^‐*p‐*cymene)] (6** **b)**: A brown one‐neck flask is charged with [(WCA‐IDipp)Cu(toluene)] (**5** **b**, 39.0 mg, 0.037 mmol, 1.0 equiv.) and suspended in toluene (4 mL). [(*η* ^6^‐*p*‐cymene)RuCl_2_(PPh_3_)] (21.0 mg, 0.037 mmol, 1.0 equiv.) is added and the mixture is stirred for 16 h and then filtered through a pad of Celite®. The solvent is removed under high vacuum and the crude product recrystallized from DCM/*n*‐hexane to obtain product **6** **b** as orange‐red crystals (55.0 mg, 0.036 mmol, 97 %). **Elemental analysis** (%) calc. for C_74_H_68_BCl_2_CuF_15_N_2_PRu⋅CH_2_Cl_2_: C 54.98, H 4.12, N 1.73; found C 55.41, H 4.10, N 1.87. ^**1**^ **H NMR** (400 MHz, dichloromethane‐*d*_2_): *δ* [ppm]=7.51–7.36 (m, 11 H, PPh_3_+*p*‐Dipp), 7.32 (d, ^3^ *J*_H,H_=7.7 Hz, 2 H, *m*‐Dipp), 7.26 (dt, ^3^ *J*_H,H_=8.0, 2.3 Hz, 6 H, PPh_3_), 7.10 (d, ^3^ *J*_H,H_=7.7 Hz, 2 H, *m*‐Dipp), 6.32 (br s, 1 H, CH=CB), 4.77 (d, ^3^ *J*_H,H_=6.0 Hz, 2 H, CH‐Cymene), 4.66 (d, ^3^ *J*_H,H_=6.1 Hz, 2 H, CH‐Cymene), 2.97 (sept, ^3^ *J*_H,H_=6.2 Hz, 2 H, CH(CH_3_)_2_‐Dipp), 2.78 (sept, ^3^ *J*_H,H_=6.3 Hz, 2 H, CH(CH_3_)_2_‐Dipp), 2.23 (sept, ^3^ *J*_H,H_=6.9 Hz, 1 H, CH(CH_3_)_2_‐Cymene), 1.81 (s, 3 H, CH_3_‐Cymene), 1.34 (d, ^3^ *J*_H,H_=6.8 Hz, 6 H, CH(CH_3_)_2_‐Dipp), 1.24 (d, ^3^ *J*_H,H_=6.8 Hz, 6 H, CH(CH_3_)_2_‐Dipp), 1.10 (d, ^3^ *J*_H,H_=6.8 Hz, 6 H, CH(CH_3_)_2_‐Dipp), 0.96 (d, ^3^ *J*_H,H_=6.9 Hz, 6 H, CH(CH_3_)_2_‐Cymene), 0.89 (d, ^3^ *J*_H,H_=6.7 Hz, 6 H, CH(CH_3_)_2_‐Dipp). ^**11**^ **B{^1^H} NMR** (128 MHz, dichloromethane‐*d*_2_): *δ* [ppm]=−15.96 (s). ^**13**^ **C{^1^H} NMR** (101 MHz, dichloromethane‐*d*_2_): *δ* [ppm]=180.9 (s, NCN), 150.5 (m, aryl‐C_6_F_5_), 148.1 (m, CH=CB), 148.0 (s, 2×*o*‐Dipp), 147.5 (s, 2×*o*‐Dipp), 140.2 (m, aryl‐C_6_F_5_), 138.9 (s, *i*‐Dipp), 138.4–137.8 (m, aryl‐C_6_F_5_), 136.9 (s, *i*‐Dipp), 135.8 (m, aryl‐C_6_F_5_), 134.2 (d, ^2^ *J*_C,P_=9.7 Hz, 6×*o*‐PPh_3_), 132.8 (d, ^1^ *J*_C,P_=47.0 Hz, 3×*i*‐PPh_3_), 131.4 (d, ^4^ *J*_C,P_=2.6 Hz, 3×*p*‐PPh_3_), 130.4 (br s, CH=CB), 129.8 (s, *p*‐Dipp), 129.2 (s, *p*‐Dipp), 128.9 (d, ^3^ *J*_C,P_=10.2 Hz, 6×*m*‐PPh_3_), 124.0 (s, 2×*m*‐Dipp), 123.2 (s, 2×*m*‐Dipp), 108.3 (s, C_Ar_‐Cymene), 97.5 (s, C_Ar_‐Cymene), 90.8 (d, *J*_C,P_=4.8 Hz, 2×CH_Ar_‐Cymene), 87.4 (d, *J*_C,P_=5.2 Hz, 2×CH_Ar_‐Cymene), 31.2 (s, CH(CH_3_)_2_‐Cymene), 28.7 (s, 2×CH(CH_3_)_2_‐Dipp), 28.3 (s, 2×CH(CH_3_)_2_‐Dipp), 27.5 (s, 2×CH(CH_3_)_2_‐Dipp), 25.2 (s, 2×CH(CH_3_)_2_‐Dipp), 24.3 (s, 2×CH(CH_3_)_2_‐Dipp), 22.4 (s, 2×CH(CH_3_)_2_‐Cymene), 22.4 (s, 2×CH(CH_3_)_2_‐Dipp), 18.3 (s, CH_3_‐Cymene). ^**19**^ **F{^1^H} NMR** (376 MHz, dichloromethane‐*d*_2_): *δ* [ppm]=−129.4 (br s, 6 F, *o*‐F), −162.9 (t, *J*=20.4 Hz, 3 F, *p*‐F), −167.5 (m, 6 F, *m*‐F). ^**31**^ **P{^1^H} NMR** (162 MHz, dichloromethane‐*d*_2_): *δ* [ppm]=24.9 (s). **HRMS** (ES−) *m/z* calcd for [(WCA‐IDipp)CuCl]^−^ (C_45_H_35_BClCuF_15_N_2_): 997.16383, found 997.16598. **HRMS** (ES−) *m/z* calcd for [((WCA‐IDipp)Cu)_2_(*μ*‐Cl)]^−^ (C_90_H_70_B_2_ClCu_2_F_30_N_4_): 1961.35700, found 1961.35881. **HRMS** (ES+) *m/z* calcd for [(*p*‐cymene)RuCl(PPh_3_)]^+^ (C_28_H_28_PRu): 533.07389, found 533.07422.

**[(WCA‐IDipp)Ag(*μ*‐I_2_)Ru(PPh_3_)(*η* ^6^‐*p‐*cymene)] (7)**: A brown one‐neck flask is charged with [(WCA‐IDipp)Ag(toluene)] (**5** **a**, 33.0 mg, 0.03 mmol, 1.0 equiv.) and dissolved in DCM (1 mL). Ruthenium complex [(*η* ^6^‐*p*‐cymene)RuI_2_(PPh_3_)] (22.5 mg, 0.03 mmol, 1.0 equiv.) is added, and the mixture is stirred for 1 h and then filtered through a pad of Celite®. The solvent is removed under high vacuum and the crude product recrystallized from DCM/*n*‐hexane at −37 °C to obtain product **7** as red crystals (46.0 mg, 0.026 mmol, 87 %). **Elemental analysis** (%) calc. for: C_73_H_68_AgBF_15_I_2_N_2_PRu: C 49.85, H 3.67, N 1.59; found C 50.07, H 3.75, N 1.70. ^**1**^ **H NMR** (400 MHz, dichloromethane‐*d*_2_): *δ* [ppm]=7.47–7.34 (m, 10 H, PPh_3_+*p*‐Dipp), 7.31–7.26 (m, 7 H, PPh_3_+*p*‐Dipp), 7.19 (d, ^3^ *J*_H,H_=7.7 Hz, 2 H, *m*‐Dipp), 7.01 (d, ^3^ *J*_H,H_=7.7 Hz, 2 H, *m*‐Dipp), 6.32 (d, ^3^ *J*_H,F_=2.2 Hz, 2 H, CH=CB), 5.15 (d, ^3^ *J*_H,H_=6.1 Hz, 2 H, CH‐Cymene), 4.83 (d, ^3^ *J*_H,H_=6.3 Hz, 2 H, CH‐Cymene), 2.98–2.82 (m, 3 H, CH(CH_3_)_2_‐Dipp+CH(CH_3_)_2_‐Cymene), 2.65 (sept, ^3^ *J*_H,H_=6.9 Hz, 2 H, CH(CH_3_)_2_‐Dipp), 1.88 (s, 3 H, CH_3_‐Cymene), 1.22 (d, ^3^ *J*_H,H_=6.9 Hz, 6 H, CH(CH_3_)_2_‐Dipp), 1.17 (d, ^3^ *J*_H,H_=6.8 Hz, 6 H, CH(CH_3_)_2_‐Dipp), 1.08 (d, ^3^ *J*_H,H_=6.8 Hz, 12 H, CH(CH_3_)_2_‐Dipp), 0.89 (d, ^3^ *J*_H,H_=6.7 Hz, 6 H, CH(CH_3_)_2_‐Cymene). ^**11**^ **B{^1^H} NMR** (128 MHz, dichloromethane‐*d*_2_): *δ* [ppm]=−16.03 (s). ^**13**^ **C{^1^H} NMR** (101 MHz, dichloromethane‐*d*_2_): *δ* [ppm]=182.8 (dd, ^1^ *J*_C,Ag_=289.9, 253.5 Hz, NCN), 150.5 (m, aryl‐C_6_F_5_), 148.1 (m, CH=CB), 147.5 (s, 2×*o*‐Dipp), 146.8 (s, 2×*o*‐Dipp), 140.3 (m, aryl‐C_6_F_5_), 138.7 (s, *i*‐Dipp), 138.1 (m, aryl‐C_6_F_5_), 136.5 (s, *i*‐Dipp), 135.8 (m, aryl‐C_6_F_5_), 135.1 (d, ^2^ *J*_C,P_=9.4 Hz, 6×*o*‐PPh_3_), 135.0 (d, ^1^ *J*_C,P_=48.0 Hz, 3×*i*‐PPh_3_), 131.3 (d, ^4^ *J*_C,P_=2.6 Hz, 3×*p*‐PPh_3_), 130.3 (br s, CH=CB), 129.8 (s, *p*‐Dipp), 129.3 (s, *p*‐Dipp), 128.5 (d, ^3^ *J*_C,P_=10.0 Hz, 6×*m*‐PPh_3_), 124.0 (s, 2×*m*‐Dipp), 123.3 (s, 2×*m*‐Dipp), 114.7 (d, ^2^ *J*_C,P_=3.9 Hz, C_Ar_‐Cymene), 101.1 (s, C_Ar_‐Cymene), 90.2 (d, ^2^ *J*_C,P_=2.4 Hz, 2×CH_Ar_‐Cymene), 88.4 (d, ^2^ *J*_C,P_=4.6 Hz, 2×CH_Ar_‐Cymene), 32.0 (s, CH(CH_3_)_2_‐Cymene), 28.5 (s, 2×CH(CH_3_)_2_‐Dipp), 28.3 (s, 2×CH(CH_3_)_2_‐Dipp), 28.2 (s, 2×CH(CH_3_)_2_‐Dipp), 25.0 (s, 2×CH(CH_3_)_2_‐Dipp), 24.8 (s, 2×CH(CH_3_)_2_‐Dipp), 23.0 (s, 2×CH(CH_3_)_2_‐Dipp), 22.3 (s, 2×CH(CH_3_)_2_‐Cymene), 19.4 (s, CH_3_‐Cymene). ^**19**^ **F{^1^H} NMR** (376 MHz, dichloromethane‐*d*_2_): *δ* [ppm]=−129.4 (br s, 6 F, *o*‐F), −163.0 (t, *J*=20.4 Hz, 3 F, *p*‐F), −167.6 (m, 6 F, *m*‐F). ^**31**^ **P{^1^H} NMR** (162 MHz, dichloromethane‐*d*_2_): *δ* [ppm]=25.4 (s). **HRMS** (ES−) *m/z* calcd for [(WCA‐IDipp)AgI]^−^ (C_45_H_35_AgBF_15_IN_2_): 1135.07460, found 1135.07703. **HRMS** (ES+) *m/z* calcd for [(*p*‐cymene)RuI(PPh_3_)]^+^ (C_28_H_29_IPRu): 625.00950, found 625.00955.

**Crystal Structures**: Crystallographic details and presentations of all compounds can be found in the Supporting Information. ref=“https://www.ccdc.cam.ac.uk/services/structures?id=doi:10.1002/chem.202102553”>2096235 (for **2**⋅THF), 2096236 (for **3**), 2096237 (for **4** **a**), 2096238 (for **4** **b**), 2096239 (for **5** **a**⋅0.5 toluene), 2096240 (for **5** **b** ⋅ 0.5 toluene), 2096241 (for **6** **a**⋅CH_2_Cl_2_), 2096242 (for **6** **b**⋅CH_2_Cl_2_), 2096243 (for **7**), 2096244 (for [Li(THF)_4_][{(WCA‐IDipp)Cu}_2_(*μ*‐Cl)] ⋅ 1.5 C_6_H_5_Cl) contain the supplementary crystallographic data for this paper. These data are provided free of charge by the joint Cambridge Crystallographic Data Centre and Fachinformationszentrum Karlsruhe http://www.ccdc.cam.ac.uk/structures “Access Structures service.”

## Conflict of interest

The authors declare no conflict of interest.

## Supporting information

As a service to our authors and readers, this journal provides supporting information supplied by the authors. Such materials are peer reviewed and may be re‐organized for online delivery, but are not copy‐edited or typeset. Technical support issues arising from supporting information (other than missing files) should be addressed to the authors.

Supporting Information

## References

[1] A. Nasr , A. Winkler , M. Tamm , Coord. Chem. Rev. 2016, 316, 68–12.

[2a] J. B. Waters , J. M. Goicoechea , Coord. Chem. Rev. 2015, 293–294, 80–94;

[2b] R. S. Ghadwal , Dalton Trans. 2016, 45, 16081–16095; 27427200 10.1039/c6dt02158a

[2c] M. Uzelac , E. Hevia , Chem. Commun. 2018, 54, 2455–2462. 10.1039/c8cc00049b 29411838

[3] Y. Wang , Y. Xie , M. Y. Abraham , P. Wei , H. F. Schaefer , P. v. R. Schleyer , G. H. Robinson , J. Am. Chem. Soc. 2010, 132, 14370–14372. 20863065 10.1021/ja106631r

[4] M. Vogt , C. Wu , A. G. Oliver , C. J. Meyer , W. F. Schneider , B. L. Ashfeld , Chem. Commun. 2013, 49, 11527–11529. 10.1039/c3cc46555a 24177269

[5a] Y. Wang , Y. Xie , M. Y. Abraham , R. J. Gilliard , P. Wei , C. F. Campana , H. F. Schaefer , P. v. R. Schleyer , G. H. Robinson , Angew. Chem. Int. Ed. Engl. 2012, 51, 10173–10176; 22962055 10.1002/anie.201204712

[5b] D. R. Armstrong , S. E. Baillie , V. L. Blair , N. G. Chabloz , J. Diez , J. Garcia-Alvarez , A. R. Kennedy , S. D. Robertson , E. Hevia , Chem. Sci. 2013, 4, 4259–4266.

[6] J. B. Waters , J. M. Goicoechea , Dalton Trans. 2014, 43, 14239–14248. 24827736 10.1039/c4dt00954a

[7] S. Kronig , E. Theuergarten , C. G. Daniliuc , P. G. Jones , M. Tamm , Angew. Chem. Int. Ed. Engl. 2012, 51, 3240–3244. 22337636 10.1002/anie.201108813

[8] E. L. Kolychev , S. Kronig , K. Brandhorst , M. Freytag , P. G. Jones , M. Tamm , J. Am. Chem. Soc. 2013, 135, 12448–12459. 23883399 10.1021/ja406529c

[9] M. Koneczny , L. Phong Ho , A. Nasr , M. Freytag , P. G. Jones , M. Tamm , Adv. Synth. Catal. 2020, 362, 3857–3863.

[10] J. Frosch , M. Freytag , P. G. Jones , M. Tamm , J. Organomet. Chem. 2020, 918, 121311.

[11] A. Winkler , K. Brandhorst , M. Freytag , P. G. Jones , M. Tamm , Organometallics 2016, 35, 1160–1169.

[12a] A. Igarashi , E. L. Kolychev , M. Tamm , K. Nomura , Organometallics 2016, 35, 1778–1784;

[12b] G. Nagai , T. Mitsudome , K. Tsutsumi , S. Sueki , T. Ina , M. Tamm , K. Nomura , J. Jpn. Pet. Inst. 2017, 60, 256–262;

[12c] K. Nomura , G. Nagai , I. Izawa , T. Mitsudome , M. Tamm , S. Yamazoe , ACS Omega 2019, 4, 18833–18845; 31737845 10.1021/acsomega.9b02828 PMC6854829

[12d] K. Nomura , G. Nagai , A. Nasr , K. Tsutsumi , Y. Kawamoto , K. Koide , M. Tamm , Organometallics 2019, 38, 3233–3244.

[13a] N. Phillips , R. Tirfoin , S. Aldridge , Dalton Trans. 2014, 43, 15279–15282; 25198297 10.1039/c4dt02662d

[13b] H. Niu , R. J. Mangan , A. V. Protchenko , N. Phillips , W. Unkrig , C. Friedmann , E. L. Kolychev , R. Tirfoin , J. Hicks , S. Aldridge , Dalton Trans. 2018, 47, 7445–7455. 29782026 10.1039/c8dt01661e

[14] L. P. Ho , L. Anders , M. Tamm , Chem. Asian J. 2020, 15, 845–851. 32011782 10.1002/asia.201901774 PMC7154526

[15a] L. P. Ho , A. Nasr , P. G. Jones , A. Altun , F. Neese , G. Bistoni , M. Tamm , Chem. Eur. J. 2018, 24, 18922–18932; 30357989 10.1002/chem.201804714

[15b] L. P. Ho , M.-K. Zaretzke , T. Bannenberg , M. Tamm , Chem. Commun. 2019, 55, 10709–10712; 10.1039/c9cc05739k 31429453

[15c] L. P. Ho , M. Tamm , Dalton Trans. 2021, 50, 1202–1205. 33480906 10.1039/d1dt00140j

[16a] L. P. Ho , L. Körner , T. Bannenberg , M. Tamm , Dalton Trans. 2020, 49, 13207–13217; 32785308 10.1039/d0dt02392b

[16b] L. P. Ho , M. Koneczny , T. Bannenberg , M. Tamm , Inorg. Chem. 2021, 60, 9019–9028. 34042436 10.1021/acs.inorgchem.1c01004

[17] J. Frosch , M. Koneczny , T. Bannenberg , M. Tamm , Chem. Eur. J. 2021, 27, 4349–4363. 33094865 10.1002/chem.202004418 PMC7986712

[18] J. Frosch , L. Körner , M. Koneczny , M. Tamm , Z. Anorg. Allg. Chem. 2021, 647, 998–1004.

[19a] J. C. Garrison , W. J. Youngs , Chem. Rev. 2005, 105, 3978–4008; 16277368 10.1021/cr050004s

[19b] I. J. B. Lin , C. S. Vasam , Coord. Chem. Rev. 2007, 251, 642–670;

[19c] F. Nahra , A. Gómez-Herrera , C. S. J. Cazin , Dalton Trans. 2017, 46, 628–631; 27882367 10.1039/c6dt03687b

[19d] T. Scattolin , S. P. Nolan , Trends Chem. 2020, 2, 721–736.

[20a] D. V. Partyka , N. Deligonul , Inorg. Chem. 2009, 48, 9463–9475; 19728693 10.1021/ic901371g

[20b] S. Tang , J. Monot , A. El-Hellani , B. Michelet , R. Guillot , C. Bour , V. Gandon , Chem. Eur. J. 2012, 18, 10239–10243; 22674541 10.1002/chem.201201202

[20c] C. Gibard , K. Fauché , R. Guillot , L. Jouffret , M. Traïkia , A. Gautier , F. Cisnetti , J. Organomet. Chem. 2017, 840, 70–74;

[20d] G. Wang , L. Pecher , G. Frenking , H. V. R. Dias , Eur. J. Inorg. Chem. 2018, 2018, 4142–4152.

[21a] A. J. Arduengo , H. V. R. Dias , J. C. Calabrese , F. Davidson , Organometallics 1993, 12, 3405–3409;

[21b] P. de Frémont , N. M. Scott , E. D. Stevens , T. Ramnial , O. C. Lightbody , C. L. B. Macdonald , J. A. C. Clyburne , C. D. Abernethy , S. P. Nolan , Organometallics 2005, 24, 6301–6309;

[21c] X.-Y. Yu , B. O. Patrick , B. R. James , Organometallics 2006, 25, 2359–2363.

[22] D. Tapu , D. A. Dixon , C. Roe , Chem. Rev. 2009, 109, 3385–3407. 19281270 10.1021/cr800521g

[23a] H. Kaur , F. K. Zinn , E. D. Stevens , S. P. Nolan , Organometallics 2004, 23, 1157–1160;

[23b] N. P. Mankad , T. G. Gray , D. S. Laitar , J. P. Sadighi , Organometallics 2004, 23, 1191–1193.

[24] S. Díez-González , E. C. Escudero-Adán , J. Benet-Buchholz , E. D. Stevens , A. M. Z. Slawin , S. P. Nolan , Dalton Trans. 2010, 39, 7595–7606. 20625598 10.1039/c0dt00218f

[25a] V. César , C. Barthes , Y. C. Farré , S. V. Cuisiat , B. Y. Vacher , R. Brousses , N. Lugan , G. Lavigne , Dalton Trans. 2013, 42, 7373–7385; 23361332 10.1039/c3dt32919d

[25b] L. Benhamou , N. Vujkovic , V. César , H. Gornitzka , N. Lugan , G. Lavigne , Organometallics 2010, 29, 2616–2630.

[26] S. Guo , M. H. Lim , H. V. Huynh , Organometallics 2013, 32, 7225–7233.

[27] H.-L. Su , L. M. Pérez , S.-J. Lee , J. H. Reibenspies , H. S. Bazzi , D. E. Bergbreiter , Organometallics 2012, 31, 4063–4071.

[28] S. M. Hubig , S. V. Lindeman , J. K. Kochi , Coord. Chem. Rev. 2000, 200–202, 831–873.

[29] M. M. D. Roy , M. J. Ferguson , R. McDonald , E. Rivard , Chem. Commun. 2018, 54, 483–486. 10.1039/c7cc08418h 29260806

[30a] N. Parvin , S. Pal , J. Echeverría , S. Alvarez , S. Khan , Chem. Sci. 2018, 9, 4333–4337; 29780565 10.1039/c8sc00459e PMC5944381

[30b] N. Parvin , J. Hossain , A. George , P. Parameswaran , S. Khan , Chem. Commun. 2019, 56, 273–276. 10.1039/c9cc09115g 31807740

[31a] N. Parvin , N. Sen , S. Tothadi , S. Muhammed , P. Parameswaran , S. Khan , Organometallics 2021, 40, 1626–1632;

[31b] M. Ghosh , S. Khan , Dalton Trans. 2021, 50, 10674–10688. 34236058 10.1039/d1dt01955d

[32] A. Jana , K. Grela , Chem. Commun. 2017, 54, 122–139. 10.1039/c7cc06535c 29188265

[33a] M. K. Karunananda , N. P. Mankad , J. Am. Chem. Soc. 2015, 137, 14598–14601; 26550848 10.1021/jacs.5b10357

[33b] S. Banerjee , M. K. Karunananda , S. Bagherzadeh , U. Jayarathne , S. R. Parmelee , G. W. Waldhart , N. P. Mankad , Inorg. Chem. 2014, 53, 11307–11315. 25275927 10.1021/ic5019778

[34] C. Cesari , S. Conti , S. Zacchini , V. Zanotti , M. C. Cassani , R. Mazzoni , Dalton Trans. 2014, 43, 17240–17243. 25338159 10.1039/c4dt02747g

[35a] B. G. Cooper , J. W. Napoline , C. M. Thomas , Catal. Rev. 2012, 54, 1–40;

[35b] P. Kalck , J. K. Bera , Eds, Homo- and heterobimetallic complexes in catalysis: Cooperative catalysis; Springer, Cham, 2016.

[36a] S. Díez-González , N. Marion , S. P. Nolan , Chem. Rev. 2009, 109, 3612–3676; 19588961 10.1021/cr900074m

[36b] L. Zhang , Z. Hou , Chem. Sci. 2013, 4, 3395;

[36c] J. D. Egbert , C. S. J. Cazin , S. P. Nolan , Catal. Sci. Technol. 2013, 3, 912;

[36d] Z. Wang , N. V. Tzouras , S. P. Nolan , X. Bi , Trends Chem. 2021.

[37] E. Hodson , S. J. Simpson , Polyhedron 2004, 23, 2695–2707.

