# Supplementary material for: Heterobimetallic Coinage Metal‐Ruthenium Complexes Supported by Anionic N‐Heterocyclic Carbenes
Source: Chemistry. 2021 Sep 3;27(61):15218–26. doi: 10.1002/chem.202102553 (PMC8597159; doi:10.1002/chem.202102553)
Supplement: Supplementary file 1 — Supporting Information [file CHEM-27-15218-s001.pdf]

Chemistry–A European Journal

Supporting Information

Heterobimetallic Coinage Metal-Ruthenium Complexes Supported by Anionic N-Heterocyclic Carbenes

Chemistry–A European Journal

Supporting Information

Heterobimetallic Coinage Metal-Ruthenium Complexes Supported by Anionic N-Heterocyclic Carbenes

Table of Contents

1 General Remarks 3

1.1 Materials and Methods 3

2 Experimental Procedures 4

2.1 [Li(THF)$_4$][(WCA-IDipp)$_2$Ag] (2) 4

2.2 [Li(THF)$_n$][(WCA-IDipp)CuCl] (3) 5

2.3 [(WCA-IDipp)Ag(PPh$_3$)] (4a) 6

2.4 [(WCA-IDipp)Cu(PPh$_3$)] (4b) 7

2.5 [(WCA-IDipp)Ag(η^2-Tol)] (5a) 8

2.6 [(WCA-IDipp)Cu(η^2-Tol)] (5b) 9

2.7 [(WCA-IDipp)Ag(μ-Cl$_2$)Ru(PPh$_3$)(η^6-*p*-cymene)] (6a) 10

2.8 [(WCA-IDipp)Cu(μ-Cl$_2$)Ru(PPh$_3$)(η^6-*p*-cymene)] (6b) 11

2.9 [(WCA-IDipp)Ag(μ-I$_2$)Ru(PPh$_3$)(η^6-*p*-cymene)] (7) 13

2.10 [(WCA-IDipp)Cu(Cl)][(Ru(η^6-*p*-cymene))$_2$(μ-Cl$_3$)] 14

3 Spectra 16

3.1 [Li(THF)$_4$][(WCA-IDipp)$_2$Ag] (2) 16

3.2 [Li(THF)$_n$][(WCA-IDipp)CuCl] (3) 19

3.3 [(WCA-IDipp)Ag(PPh$_3$)] (4a) 21

3.4 [(WCA-IDipp)Cu(PPh$_3$)] (4b) 24

3.5 [(WCA-IDipp)Ag(η^2-Tol)] (5a) 27

3.6 [(WCA-IDipp)Cu(η^2-Tol)] (5b) 29

3.7 [(WCA-IDipp)Ag(μ-Cl$_2$)Ru(PPh$_3$)(η^6-*p*-cymene)] (6a) 31

3.8 [(WCA-IDipp)Cu(μ-Cl$_2$)Ru(PPh$_3$)(η^6-*p*-cymene)] (6b) 34

3.9 [(WCA-IDipp)Ag(μ-I$_2$)Ru(PPh$_3$)(η^6-*p*-cymene)] (7) 36

3.10 [(WCA-IDipp)Cu(Cl)][(Ru(η^6-*p*-cymene))$_2$(μ-Cl$_3$)] 39

4 X-Ray Crystal Structure Determinations 41

4.1 [Li(THF)$_4$][(WCA-IDipp)$_2$Ag] (2·THF) 42

4.2 [Li(THF)$_n$][(WCA-IDipp)CuCl] (3) 44
4.3 [(WCA-IDipp)Ag(PPh$_3$)] (4a) 46
4.4 [(WCA-IDipp)Cu(PPh$_3$)] (4b) 48
4.5 [(WCA-IDipp)Ag(η^2-Tol)] (5a) 50
4.6 [(WCA-IDipp)Cu(η^2-Tol)] (5b) 52
4.7 [(WCA-IDipp)Ag(μ-Cl$_2$)Ru(PPh$_3$)(η^6-*p*-cymene)] (6a·CH$_2$Cl$_2$) 54
4.8 [(WCA-IDipp)Cu(μ-Cl$_2$)Ru(PPh$_3$)(η^6-*p*-cymene)] (6b·CH$_2$Cl$_2$) 56
4.9 [(WCA-IDipp)Ag(μ-I$_2$)Ru(PPh$_3$)(η^6-*p*-cymene)] (7·solvent) 58
4.10 [(WCA-IDipp)Cu(Cl)][(Ru(η^6-*p*-cymene))$_2$(μ-Cl$_3$)] 60
4.11 [Li(THF)$_4$][((WCA-IDipp)Cu)$_2$(μ-Cl)]·1.5C$_6$H$_5$Cl 61

1 General Remarks

1.1 Materials and Methods

All operations with air- and moisture-sensitive compounds were performed in a glove box under a dry argon atmosphere (MBraun 200B) or on a vacuum line using Schlenk techniques. All solvents were distilled from Na/benzophenone or CaH_2, degassed prior to use and stored over molecular sieves (4 Å). The ^{1}H, $^{13}C\{^{1}H\}$, $^{11}B\{^{1}H\}$, $^{19}F\{^{1}H\}$ and $^{31}P\{^{1}H\}$ NMR spectra were recorded on Bruker DPX 200, Bruker AV 300, Bruker DRX 400, Bruker AV II 600, AVIII400, AVIIIHD500 and Agilent Mercury 400 spectrometers at room temperature. ^{1}H and $^{13}C\{^{1}H\}$ NMR spectra were referenced against the (residual) solvent signals.[1] Boron trifluoride diethyl etherate ($BF_3{\cdot}OEt_2$) was used as external reference for $^{11}B\{^{1}H\}$.[2] Trichlorofluoromethane ($CFCl_3$) or hexafluorobenzene (C_6F_6) was used as external reference for $^{19}F\{^{1}H\}$. For $^{31}P\{^{1}H\}$ NMR spectra H_3PO_4 was used as external reference.[2] Chemical shifts are reported in ppm (parts per million). $^{11}B\{^{1}H\}$, $^{13}C\{^{1}H\}$, $^{19}F\{^{1}H\}$ and $^{31}P\{^{1}H\}$ NMR spectra were obtained applying composite pulse proton decoupling. Coupling constants (J) are reported in Hertz (Hz), and splitting patterns are indicated as s (singlet), d (doublet), t (triplet), q (quartet), m (multiplet), sept (septet) and br (broad). NMR assignments were made using additional 2D NMR experiments.

Elemental analysis was carried out with a Vario Micro Cube System or a Vario EL III apparatus (Polish Academy of Science, Institute of Organic Chemistry). Unless otherwise indicated, all starting materials were obtained from Sigma-Aldrich, ABRC, TCI, Acros or Fluka and were purified if necessary. [(WCA-IDipp)Li(toluene)][3] and [(η^6-*p*-cymene)$RuCl_2(PPh_3)$][4] were prepared according to literature procedures.

[1] G. R. Fulmer, A. J. M. Miller, N. H. Sherden, H. E. Gottlieb, A. Nudelman, B. M. Stoltz, J. E. Bercaw, K. I. Goldberg, *Organometallics* **2010**, *29*, 2176.

[2] R. K. Harris, E. D. Becker, S. M. C. de Menezes, P. Granger, R. E. Hoffman, K. W. Zilm, *Magn. Reson. Chem.* **2008**, *46*, 582.

[3] S. Kronig, E. Theuergarten, C. G. Daniliuc, P. G. Jones, M. Tamm, *Angew. Chem. Int. Ed.* **2012**, *51*, 3240.

[4] E. Hodson, S. J. Simpson, *Polyhedron* **2004**, *23*, 2695–2707.

2 Experimental Procedures

2.1 [Li(THF)$_4$][(WCA-IDipp)$_2$Ag] (2)

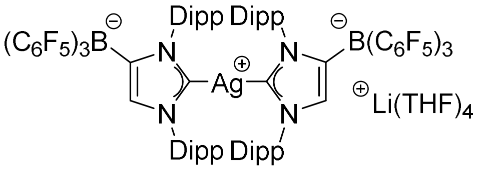

A brown vial ischarged with [(WCA-IDipp)Li(toluene)] (**1**, 100 mg, 0.1 mmol, 1 equiv.) and AgCl (14.3 mg, 0.1 mmol, 1 equiv.) dissolved in THF (2 mL). The solution is stirred overnight and then concentrated under high vacuum and layered with *n*-hexane. After 24 h at −40 °C the supernatant solution is removed and the crude product washed with *n*-hexane (2 × 2 mL). The crude product is then recrystallized from THF/*n*-hexane to obtain product **2** as colorless crystals (43 mg, 0.019 mmol, 39%).

elemental analysis (%) calc. for $C_{106}H_{102}AgB_2F_{30}LiN_4O_4$: C 57.81, H 4.67, N 2.54; found C 58.22, H 5.125, N 2.39.

^{1}H NMR (400 MHz, THF-*d*$_8$) for = 3: δ [ppm] = 7.29 (t, $^3J_{H,H}$ = 7.51 Hz, 2 H, *p*-Dipp), 7.21 (t, $^3J_{H,H}$ = 7.51 Hz, 2 H, *p*-Dipp), 7.02 (d, $^3J_{H,H}$ = 7.76 Hz, 4 H, *m*-Dipp), 6.81 (d, $^3J_{H,H}$ = 7.76 Hz, 4 H, *m*-Dipp), 6.21 (s, 2 H, C<u>H</u>=CB), 3.64−3.59 (m, 12 H, THF), 2.72 (sept, $^3J_{H,H}$ = 6.76 Hz, 4 H, C<u>H</u>(CH$_3$)$_2$), 2.44 (sept, $^3J_{H,H}$ = 6.68 Hz, 4 H, C<u>H</u>(CH$_3$)$_2$), 1.79−1.74 (m, 12 H, THF), 0.88 (d, $^3J_{H,H}$ = 6.76 Hz, 12 H, CH(C<u>H</u>$_3$)$_2$), 0.79−0.74 (m, 24 H, CH(C<u>H</u>$_3$)$_2$), 0.72 (d, $^3J_{H,H}$ = 6.92 Hz, 12 H, CH(C<u>H</u>$_3$)$_2$.

^{11}B{^{1}H} NMR (160 MHz, THF-*d*$_8$): δ [ppm] = −15.23 (s).

^{13}C{^{1}H} NMR (125 MHz, THF-*d*$_8$): δ [ppm] = 183.9 (2×d, $^1J_{C,Ag}$ = 224 Hz, 195 Hz, NCN), 150.5 (m, aryl-C$_6$F$_5$) 148.6 (m, 2×<u>C</u>B=CH), 146.9 (s, 4×*o*-Dipp), 146.4 (s, 4×*o*-Dipp), 140.0 (m, aryl-C$_6$F$_5$), 138.5 (s, 2×*i*-Dipp), 138.1 (m, aryl-C$_6$F$_5$), 136.9 (s, 2×*i*-Dipp),132.7 (s, 2×<u>C</u>H=CB), 130.0 (s, 2×*p*-Dipp), 129.4 (s, 2×*p*-Dipp), 124.6 (s, 4×*m*-Dipp), 124.0 (s, 4×*m*-Dipp), 68.0 (s, CH$_2$-THF), 28.4 (s, 4×<u>C</u>H(CH$_3$)$_2$), 28.0 (s, 4×<u>C</u>H(CH$_3$)$_2$), 27.8 (s, 4×CH(<u>C</u>H$_3$)$_2$), 26.1 (s, CH$_2$-THF), 24.9 (s, 4×CH(<u>C</u>H$_3$)$_2$), 23.3 (s, 4×CH(<u>C</u>H$_3$)$_2$), 21.8 (s, 4×CH(<u>C</u>H$_3$)$_2$).

^{19}F{^{1}H} NMR (376 MHz, THF-*d*$_8$): δ [ppm] = −129.5 (br s, 6 F, *o*-F), −163.8 (t, *J* = 20.3 Hz, 3 F, *p*-F), −168.2 (t, *J* = 18.7 Hz, 6 F, *m*-F).

2.2 [Li(THF)$_n$][(WCA-IDipp)CuCl] (3)

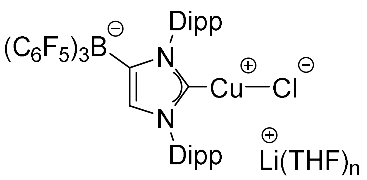

A one-neck flask is charged with [(WCA-IDipp)Li(toluene)] (**1**, 150 mg, 0.15 mmol, 1.0 equiv.) and dissolved in THF (4 mL). CuCl (15.0 mg, 0.15 mmol, 1.0 equiv.) is added and the solution is stirred for 2 h and then filtered through a pad of Celite®. The solvent is removed under high vacuum and the crude product recrystallized from THF/*n*-hexane to obtain product **3** as colorless crystals (186.0 mg, 0.14 mmol, 96%).

elemental analysis (%) calc. for n = 3: $C_{57}H_{59}BClCuF_{15}LiN_2O_3$: C 56.35, H 4.90, N 2.31; found C 56.17, H 4.63, N 2.30.

^{1}H NMR (400 MHz, dichloromethane-*d*$_2$): *δ* [ppm] = 7.39 (t, $^3J_{H,H}$ = 7.7 Hz, 1 H, *p*-Dipp), 7.28 (t, $^3J_{H,H}$ = 7.7 Hz, 1 H, *p*-Dipp), 7.19 (d, $^3J_{H,H}$ = 7.8 Hz, 2 H, *m*-Dipp), 6.97 (d, $^3J_{H,H}$ = 7.7 Hz, 2 H, *m*-Dipp), 6.27 (br s, 1 H, $C\underline{H}$=CB), 3.69−3.54 (m, 12 H, THF), 2.85 (sept, $^3J_{H,H}$ = 6.5 Hz, 2 H, $C\underline{H}(CH_3)_2$), 2.64 (sept, $^3J_{H,H}$ = 6.8 Hz, 2 H, $C\underline{H}(CH_3)_2$), 1.90−1.78 (m, 11 H, THF), 1.16−1.02 (m, 18 H, $CH(C\underline{H}_3)_2$), 0.90 (d, $^3J_{H,H}$ = 6.7 Hz, 6 H, $C\underline{H}(CH_3)_2$).

^{11}B{^{1}H} NMR (128 MHz, dichloromethane-*d*$_2$): *δ* [ppm] = −16.18 (s).

^{13}C{^{1}H} NMR (101 MHz, dichloromethane-*d*$_2$): *δ* [ppm] = 177.2 (s, NCN), 150.4 (m, aryl-C_6F_5), 148.0 (m, CH=$\underline{C}$B), 147.7 (s, 2×*o*-Dipp), 146.7 (s, 2×*o*-Dipp), 140.3 (m, aryl-C_6F_5), 138.2 (m, aryl-C_6F_5), 137.5 (s, 2×*i*-Dipp), 135.9 (m, aryl-C_6F_5), 135.8 (s, 2×*i*-Dipp), 130.4 (br s, $\underline{C}$H=CB), 130.1 (s, 2×*p*-Dipp), 129.6 (s, 2×*p*-Dipp), 124.1 (s, 2×*m*-Dipp), 123.2 (s, 2×*m*-Dipp), 68.8 (s, CH_2-THF), 28.5 (s, 2×$\underline{C}H(CH_3)_2$), 28.1 (s, 2×$CH(\underline{C}H_3)_2$), 27.5 (s, 2×$\underline{C}H(CH_3)_2$), 25.9 (s, 2×$CH(\underline{C}H_3)_2$), 25.0 (s, 2×$CH(\underline{C}H_3)_2$), 24.1 (s, 2×$CH(\underline{C}H_3)_2$), 22.0 (s, 2×$CH(\underline{C}H_3)_2$).

^{19}F{^{1}H} NMR (376 MHz, dichloromethane-*d*$_2$): *δ* [ppm] = −129.3 (br s, 6 F, *o*-F), −162.8 (t, *J* = 20.4 Hz, 3 F, *p*-F), −167.5 (t, *J* = 18.5 Hz, 6 F, *m*-F).

HRMS (ES−) *m/z* calcd for [(WCA-IDipp)CuCl]$^-$ ($C_{45}H_{35}BCuClF_{15}N_2$): 997.1638, found 997.1628.

2.3 [(WCA-IDipp)Ag(PPh_3)] (4a)

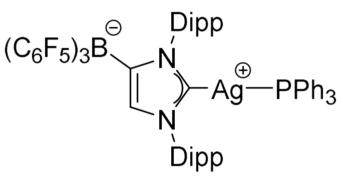

A brown one-neck flask is charged with [(WCA-IDipp)Li(toluene)] (**1**, 150 mg, 0.15 mmol, 1.0 equiv.) and suspended in toluene (8 mL). Chloro(triphenylphos-phine)silver(I) (61.0 mg, 0.15 mmol, 1.0 equiv.) is added and the suspension is stirred for 2 h and then filtered through a pad of Celite®. The solvent is removed under high vacuum and the crude product recrystallized from DCM/*n*-hexane to obtain product **4a** as colorless crystals (130.0 mg, 0.10 mmol, 69%).

elemental analysis (%) calc. for $C_{63}H_{50}AgBF_{15}N_2P$: C 59.59, H 3.97, N 2.21; found C 59.71, H 4.00, N 2.14.

^{1}H NMR (400 MHz, dichloromethane-d_2): δ [ppm] = 7.50−7.43 (m, 4 H, PPh_3), 7.41 (d, $^3J_{H,H}$ = 7.7 Hz, 1 H, *p*-Dipp) 7.36−7.27 (m, 6 H, PPh_3 + *p*-Dipp), 7.25 (d, $^3J_{H,H}$ = 7.8 Hz, 2 H, *m*-Dipp), 7.08 (d, $^3J_{H,H}$ = 7.8 Hz, 2 H, *m*-Dipp), 6.95−6.85 (m, 6 H, PPh_3), 6.47 (br s, 1 H), 2.97 (sept, $^3J_{H,H}$ = 6.7 Hz, 2 H, $C\underline{H}(CH_3)_2$), 2.73 (sept, $^3J_{H,H}$ = 6.5 Hz, 2 H, $C\underline{H}(CH_3)_2$), 1.14 (d, $^3J_{H,H}$ = 6.8 Hz, 6 H, $CH(C\underline{H}_3)_2$), 1.09 (m, 12 H, $CH(C\underline{H}_3)_2$), 0.93 (d, $^3J_{H,H}$ = 6.7 Hz, 6 H, $CH(C\underline{H}_3)_2$).

^{11}B{^{1}H} NMR (128 MHz, dichloromethane-d_2): δ [ppm] = −16.12 (s).

^{13}C{^{1}H} NMR (101 MHz, dichloromethane-d_2): δ [ppm] = 150.5 (m, aryl-C_6F_5), 148.2 (m, CH=$\underline{C}$B), 147.9 (s, 2×*o*-Dipp), 146.9 (s, 2×*o*-Dipp), 140.4 (m, aryl-C_6F_5), 138.4 (m, aryl-C_6F_5), 137.9 (s, 2×*i*-Dipp), 136.0 (m, aryl-C_6F_5), 135.7 (s, 2×*i*-Dipp), 134.1 (dd, $^2J_{C,P}$ = 15.9, $J_{C,Ag}$ = 2.6 Hz, *o*-PPh_3), 132.1 (d, $^4J_{C,P}$ = 2.2 Hz, *p*-PPh_3), 130.8 (br s, $\underline{C}$H=CB), 130.5 (s, 2×*p*-Dipp), 130.0 (s, 2×*p*-Dipp), 129.9 (d, $^3J_{C,P}$ = 10.9 Hz, *m*-PPh_3), 129.0 (dd, $^1J_{C,P}$ = 42.3, $J_{C,Ag}$ = 3.3 Hz, *i*-PPh_3), 124.4 (s, 2×*m*-Dipp), 123.7 (s, 2×*m*-Dipp), 28.6 (s, 2×$\underline{C}H(CH_3)_2$), 28.2 (s, 2×$CH(\underline{C}H_3)_2$), 28.0 (s, 2×$\underline{C}H(CH_3)_2$), 25.1 (s, 2×$CH(\underline{C}H_3)_2$), 24.4 (s, 2×$CH(\underline{C}H_3)_2$), 22.1 (s, 2×$CH(\underline{C}H_3)_2$).

^{19}F{^{1}H} NMR (376 MHz, dichloromethane-d_2): δ [ppm] = −129.5 (br s, 6 F, *o*-F), −162.4 (t, J = 20.3 Hz, 3 F, *p*-F), −167.3 (t, J = 18.6 Hz, 6 F, *m*-F).

^{31}P{^{1}H} NMR (162 MHz, dichloromethane-d_2): δ [ppm] = 18.3 (dd, $^1J_{P,Ag}$ = 532.9, 461.7 Hz).

HRMS (ES−) *m/z* calcd for [(WCA-IDipp)AgCl]$^-$ ($C_{45}H_{35}BAgClF_{15}N_2$): 1041.1393, found 1041.1398.

2.4 [(WCA-IDipp)Cu(PPh_3)] (4b)

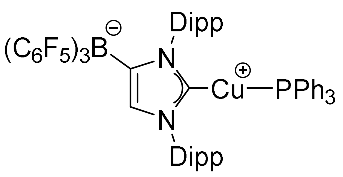

A Schlenk flask was charged with chloro(triphenylphosphine)copper(I) (36.17 mg, 0.1 mmol, 1 equiv.) and [(WCA-IDipp)Li(toluene)] (**1**, 100.0 mg, 0.1 mmol, 1 equiv.) dissolved in chlorobenzene (5 mL) under exclusion of light. The mixture is stirred at room temperature for 2.5 h and then filtered through a pad of Celite®. The solvent is removed under high vacuum and the yellowish solid recrystallized from THF/DCM layered with *n*-hexane to obtain product **4b** as yellow crystals (77.0 mg, 0.063 mmol, 63%).

elemental analysis (%) calc. for. $C_{63}H_{50}BCuF_{15}N_2P$: C 61.75, H 4.11, N, 2.29; found C 62.09, H 4.375, N 2.31.

^{1}H NMR (500 MHz, THF-d_8): δ [ppm] = 7.46 (m, 5 H, PPh_3), 7.32 (m, 8 H, PPh_3 + aryl-Dipp), 7.16 (d, $^3J_{H,H}$ = 6.80 Hz, 2 H, *m*-Dipp), 6.87 (m, 6 H, PPh_3 + aryl-Dipp), 6.67 (s, 1 H, C$\underline{H}$=CB), 3.04 (sept, $^3J_{H,H}$ = 6.80 Hz, 2 H, C$\underline{H}$(CH_3)$_2$), 2.78 (sept, $^3J_{H,H}$ = 6.86 Hz, 2 H, C$\underline{H}$(CH_3)$_2$), 1.13 (d, $^3J_{H,H}$ = 6.85 Hz, 6 H, CH(C$\underline{H}_3$)$_2$), 1.08 (m, 12 H, CH(C$\underline{H}_3$)$_2$), 0.96 (d, $^3J_{H,H}$ = 6.83 Hz, 6 H, CH(C$\underline{H}_3$)$_2$).

^{11}B{^{1}H} NMR (160 MHz, THF-d_8): δ [ppm] = −15.58 (s).

^{13}C{^{1}H} NMR (125 MHz, THF-d_8): δ [ppm] = 177.2 (d, $^2J_{C,P}$ = 68.6 Hz, NCN), 150.5 (m, aryl-C_6F_5), 148.6 (m, aryl-C_6F_5 + CH=$\underline{C}$B), 148.1 (s, 2×*o*-Dipp), 147.0 (s, 2×*o*-Dipp), 140.3 (m, aryl-C_6F_5), 138.4 (m, aryl-C_6F_5), 137.8 (s, *i*-Dipp), 136.3 (m, aryl-C_6F_5), 135.7 (s, *i*-Dipp), 134.2 (d, $^2J_{C,P}$ = 14.8 Hz, *o*-PPh_3), 132.2 (s, *p*-PPh_3), 131.2 (br s, $\underline{C}$H=CB), 130.5 (d, $^1J_{C,P}$ = 42.4 Hz, *i*-PPh_3), 130.0 (d $^3J_{C,P}$ = 10.6 Hz, *m*-PPh_3), 129.1 (s, *p*-Dipp), 128.7 (s, *p*-Dipp), 124.5 (s, 2×*m*-Dipp), 123.8 (s, 2×*m*-Dipp), 28.8 (s, 2×$\underline{C}$H(CH_3)$_2$), 28.4 (s, 2×CH($\underline{C}H_3$)$_2$), 28.3 (s, 2×$\underline{C}$H(CH_3)$_2$), 24.7 (s, 2×CH($\underline{C}H_3$)$_2$), 24.4 (s, 2×CH($\underline{C}H_3$)$_2$), 21.6 (s, 2×CH($\underline{C}H_3$)$_2$).

^{19}F{^{1}H} NMR (470 MHz, THF-d_8): δ [ppm] = −128.5 (br s, 6 F, *o*-F), −162.6 (m, 3 F, *p*-F), −167.0 (s, 6 F, *m*-F).

$^{31}P\{^1H\}$ **NMR** (202 MHz, THF-d_8): δ [ppm] = 8.6 (s).

2.5 [(WCA-IDipp)Ag(η^2-Tol)] (5a)

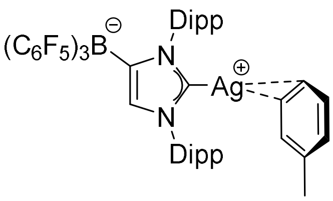

A brown one-neck flask is charged with [(WCA-IDipp)Li(toluene)] (**1**, 100 mg, 0.1 mmol, 1.0 equiv.) and suspended in toluene (5 mL). Silver(**I**) trifluoromethanesulfonate (25.7 mg, 0.1 mmol, 1.0 equiv.) is added and the suspension is stirred for 10 minutes and then filtered through a pad of Celite®. The solvent is removed under high vacuum and the crude product recrystallized from toluene/DCM to obtain product **5a** as colorless crystals (90.0 mg, 0.08 mmol, 82%).

elemental analysis (%) calc. for $C_{52}H_{43}AgBF_{15}N_2 \cdot ½C_7H_8$: C 58.19, H 4.14, N 2.45; found C 58.06, H 3.76, N 2.42.

^1H **NMR** (400 MHz, THF-d_8): δ [ppm] = 7.44−7.38 (t, $^3J_{H,H}$ = 8.0 Hz, 1 H, *p*-Dipp), 7.32 (t, $^3J_{H,H}$ = 7.8 Hz, 2 H, *p*-Dipp) 7.28 (d, $^3J_{H,H}$ = 7.7 Hz, 2 H, *m*-Dipp), 7.21−7.17 (m, 2 H, Toluene), 7.16−7.04 (m, 5 H, Toluene + *m*-Dipp), 6.58 (br s, 1 H, C<u>H</u>=CB), 3.03 (sept, $^3J_{H,H}$ = 6.8 Hz, 2 H, C<u>H</u>(CH_3)$_2$), 2.77 (sept, $^3J_{H,H}$ = 6.9 Hz, 2 H, C<u>H</u>(CH_3)$_2$), 2.31 (s, 4 H, Toluene), 1.22 (dd, $^3J_{H,H}$ = 9.1, 6.8 Hz, 12 H, CH(C<u>H</u>$_3$)$_2$), 1.14 (d, $^3J_{H,H}$ = 6.8 Hz, 6 H, CH(C<u>H</u>$_3$)$_2$), 0.97 (d, $^3J_{H,H}$ = 6.7 Hz, 6 H, CH(C<u>H</u>$_3$)$_2$).

$^{11}B\{^1H\}$ **NMR** (128 MHz, dichloromethane-d_2): δ [ppm] = −16.04 (s).

$^{13}C\{^1H\}$ **NMR** (101 MHz, THF-d_8): δ [ppm] = 182.1 (dd, $^2J_{C,Ag}$ = 299.9, 347.4 Hz, NCN), 151.2 (m, aryl-C_6F_5), 148.8 (m, <u>C</u>B=CH), 148.4 (s, 2×*o*-Dipp), 147.4 (s, 2×*o*-Dipp), 140.9 (m, aryl-C_6F_5), 139.5 (s, *i*-Dipp), 138.7 (m, aryl-C_6F_5), 138.6 (s, C_1-Tol), 137.5 (s, *i*-Dipp), 136.4 (m, aryl-C_6F_5), 131.5 (br s, <u>C</u>H=CB), 130.6 (s, *p*-Dipp), 130.2 (s, *p*-Dipp), 129.8 (s, $C_{2,6}$-Tol), 129.1 (s, $C_{3,5}$-Tol), 126.2 (s, C_4-Tol), 124.8 (s, *m*-Dipp), 124.1 (s, *m*-Dipp), 29.0 (s, 2×<u>C</u>H(CH_3)$_2$), 28.6 (s, 2×CH(<u>C</u>H$_3$)$_2$), 27.8 (s, 2×<u>C</u>H(CH_3)$_2$), 25.1 (s, 2×CH(<u>C</u>H$_3$)$_2$), 24.4 (s, 2×CH(<u>C</u>H$_3$)$_2$), 22.3 (s, 2×CH(<u>C</u>H$_3$)$_2$), 21.6 (CH_3-Tol).

$^{19}F\{^1H\}$ **NMR** (376 MHz, THF-d_8): δ [ppm] = −129.5 (br s, 6 F, *o*-F), −163.8 (t, *J* = 20.3 Hz, 3 F, *p*-F), −168.2 (t, *J* = 18.7 Hz, 6 F, *m*-F).

HRMS (ES−) *m/z* calcd for [(WCA-IDipp)AgCl]$^-$ ($C_{45}H_{35}BAgClF_{15}N_2$): 1041.1393, found 1041.1385.

2.6 [(WCA-IDipp)Cu(η^2-Tol)] (5b)

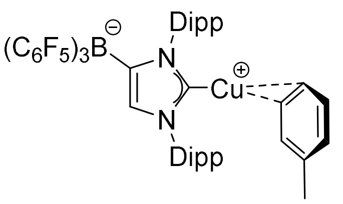

A one-neck flask is charged with [(WCA-IDipp)Li(toluene)] (**1**, 120 mg, 0.12 mmol, 1.0 equiv.) and suspended in toluene (5 mL). CuCl (24.0 mg, 0.24 mmol, 2.0 equiv.) is added and the suspension is stirred for 16 h and then filtered through a pad of Celite®. The solvent is removed under high vacuum and the crude product recrystallized from toluene/diethylether to obtain product **5b** as colorless crystals (115.0 mg, 0.11 mmol, 91%).

elemental analysis (%) calc. for $C_{52}H_{43}BCuF_{15}N_2 \cdot C_7H_8$: C 61.76, H 4.48, N 2.44; found C 61.74, H 4.42, N 2.67.

^{1}H NMR (400 MHz, dichloromethane-*d*$_2$): δ [ppm] = 7.47 (t, $^3J_{H,H}$ = 7.8 Hz, 1 H, *p*-Dipp), 7.41 (t, $^3J_{H,H}$ = 7.8 Hz, 1 H, *p*-Dipp), 7.26 (d, $^3J_{H,H}$ = 7.8 Hz, 2 H, *m*-Dipp), 7.10 (d, *J* = 7.7 Hz, 2 H, *m*-Dipp), 7.04−7.00 (m, 5 H, Toluene), 6.89−6.80 (m, 1 H, Toluene), 6.30 (br s, 1 H, $C\underline{H}$=CB), 2.75 (sept, $^3J_{H,H}$ = 6.8 Hz, 2 H, $C\underline{H}(CH_3)_2$), 2.41 (sept, $^3J_{H,H}$ = 7.0 Hz, 2 H, $C\underline{H}(CH_3)_2$), 2.12 (s, 5 H, Toluene), 1.18 (d, $^3J_{H,H}$ = 6.9 Hz, 6 H, $CH(C\underline{H}_3)_2$), 1.08 (d, $^3J_{H,H}$ = 6.8 Hz, 6 H, $CH(C\underline{H}_3)_2$), 1.04 (d, $^3J_{H,H}$ = 6.8 Hz, 6 H, $CH(C\underline{H}_3)_2$), 0.88 (d, $^3J_{H,H}$ = 6.7 Hz, 6 H, $CH(C\underline{H}_3)_2$).

^{11}B{^{1}H} NMR (128 MHz, dichloromethane-*d*$_2$): δ [ppm] = −16.21 (s).

^{13}C{^{1}H} NMR (101 MHz, dichloromethane-*d*$_2$): δ [ppm] =175.1 (s, NCN), 150.4 (m, aryl-C_6F_5), 148.0 (s, CH=$\underline{C}$B), 147.4 (s, 2×*o*-Dipp), 146.5 (s, 2×*o*-Dipp), 140.4 (m, aryl-C_6F_5), 139.5 (s, *i*-Dipp), 138.3 (m, aryl-C_6F_5), 138.1 (s, C_1-Tol), 135.9 (m, aryl-C_6F_5), 135.4 (s, *i*-Dipp), 130.8 ($\underline{C}$H=CB), 130.5 (s, *p*-Dipp), 130.0 (s, *p*-Dipp), 127.5 (s, $C_{2,6}$-Tol), 125.1 (br s, $C_{3,5}$-Tol), 124.2 (s, *m*-Dipp), 123.6 (s, *m*-Dipp), 121.3 (s, C_4-Tol), 28.5 (s, 2×$\underline{C}H(CH_3)_2$), 28.1 (s, 2×$CH(\underline{C}H_3)_2$), 27.7 (s, 2×$\underline{C}H(CH_3)_2$), 24.9 (s, 2×$CH(\underline{C}H_3)_2$), 24.4 (s, 2×$CH(\underline{C}H_3)_2$), 22.1 (s, 2×$CH(\underline{C}H_3)_2$), 21.6 (CH_3-Tol).

^{19}F{^{1}H} NMR (376 MHz, dichloromethane-*d*$_2$): δ [ppm] = −129.6 (br s, 6 F, *o*-F), −163.4 (t, *J* = 20.4 Hz, 3 F, *p*-F), −167.3 (t, *J* = 18.5 Hz, 6 F, *m*-F).

HRMS (ES−) *m/z* calcd for [(WCA-IDipp)CuCl]$^-$ ($C_{45}H_{35}BCuClF_{15}N_2$): 997.1638, found 997.1624.

2.7 [(WCA-IDipp)Ag(μ-Cl_2)Ru(PPh_3)(η^6-*p*-cymene)] (6a)

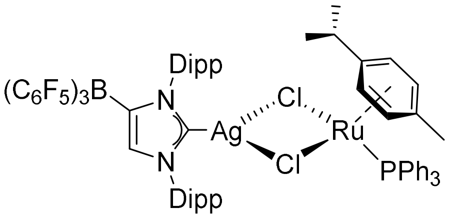

A brown one-neck flask is charged with [(WCA-IDipp)Ag(toluene)] (**5a**, 55.0 mg, 0.05 mmol, 1.0 equiv.) and suspended in toluene (5 mL). Ruthenium complex [(η^6-*p*-cymene)RuCl_2(PPh_3)] (28.4 mg, 0.05 mmol, 1.0 equiv.) is added and the mixture is stirred for 1 h and then filtered through a pad of Celite®. The solvent is removed under high vacuum and the crude product recrystallized at −37 °C from DCM/*n*-hexane to obtain product **6a** as orange/red crystals (54.0 mg, 0.034 mmol, 69%).

elemental analysis (%) calc. for: $C_{73}H_{64}AgBCl_2F_{15}N_2PRu·CH_2Cl_2$: C 53.72, H 4.21, N 1.67; found C 53.98, H 3.78, N 1.73.

^{1}H NMR (400 MHz, dichloromethane-*d*$_2$): δ [ppm] = 7.55−7.34 (m, 11 H, PPh_3 + *p*-Dipp), 7.33−7.21 (m, 8 H, PPh_3 + *m*-Dipp), 7.10 (d, $^3J_{H,H}$ = 7.7 Hz, 2 H, *m*-Dipp), 6.39 (d, $J_{H,F}$ = 2.3 Hz, 1 H), 4.83 (d, $^3J_{H,H}$ = 6.0 Hz, 2 H, CH-Cymene), 4.71 (d, $^3J_{H,H}$ = 6.1 Hz, 2 H, CH-Cymene), 2.94 (sept, $^3J_{H,H}$ = 6.8 Hz, 2 H, C$\underline{H}$(CH_3)$_2$-Dipp), 2.73 (sept, $^3J_{H,H}$ = 6.6 Hz, 2 H, C$\underline{H}$(CH_3)$_2$-Dipp), 2.28 (sept, $^3J_{H,H}$ = 6.8 Hz, 1 H, C$\underline{H}$(CH_3)$_2$-Cymene), 1.83 (s, 3 H), 1.29 (d, $^3J_{H,H}$ = 6.9 Hz, 6 H, CH(C$\underline{H}_3$)$_2$), 1.20 (d, $^3J_{H,H}$ = 6.8 Hz, 6 H, CH(C$\underline{H}_3$)$_2$), 1.12 (d, $^3J_{H,H}$ = 6.8 Hz, 6 H, CH(C$\underline{H}_3$)$_2$), 0.98 (d, $^3J_{H,H}$ = 6.9 Hz, 6 H, CH(C$\underline{H}_3$)$_2$), 0.91 (d, $^3J_{H,H}$ = 6.7 Hz, 6 H, CH(C$\underline{H}_3$)$_2$).

^{11}B{^{1}H} NMR (128 MHz, dichloromethane-*d*$_2$): δ [ppm] = −16.02 (s).

^{13}C{^{1}H} NMR (101 MHz, dichloromethane-*d*$_2$): δ [ppm] = 185.0 (dd, $^1J_{C,Ag}$ = 317.1, 274.7 Hz, NCN) 150.5 (m, aryl-C_6F_5), 148.2 (m, CH=$\underline{C}$B), 148.0 (s, 2×*o*-Dipp), 147.4 (s, 2×*o*-Dipp), 140.3 (m, aryl-C_6F_5), 138.9 (s, 2×*i*-Dipp), 138.1 (m, aryl-C_6F_5), 136.9 (s, 2×*i*-Dipp), 135.8 (m, aryl-C_6F_5), 134.2 (d, $^2J_{C,P}$ = 9.6 Hz, *o*-PPh_3), 132.8 (d, $^1J_{C,P}$ = 46.7 Hz, *i*-PPh_3), 131.4 (d, $^4J_{C,P}$ = 2.2 Hz, *p*-PPh_3), 130.4 (br s, $\underline{C}$H=CB), 129.9 (s, 2×*p*-Dipp), 129.3 (s, 2×*p*-Dipp), 128.8 (d, $^3J_{C,P}$ = 10.1 Hz, *m*-PPh_3), 124.0 (s, 2×*m*-Dipp), 123.3 (s, 2×*m*-Dipp), 109.4 (s, C_{Ar}-Cymene), 97.3 (s, C_{Ar}-Cymene), 90.9 (d, $^2J_{C,P}$ = 4.0

Hz, CH_{Ar}-Cymene), 86.7 (d, $^2J_{CP}$ = 5.1 Hz, CH_{Ar}-Cymene), 31.1 (s, <u>C</u>H(CH_3)$_2$-Cymene), 28.6 (s, 2×<u>C</u>H(CH_3)$_2$-Dipp), 28.2 (s, 2×CH(<u>C</u>H_3)$_2$-Dipp), 27.6 (s, 2×<u>C</u>H(CH_3)$_2$-Dipp), 24.8 (s, 2×CH(<u>C</u>H_3)$_2$-Dipp), 24.7 (s, 2×CH(<u>C</u>H_3)$_2$-Dipp), 22.4 (s, 2×CH(<u>C</u>H_3)$_2$-Cymene), 22.4 (s, 2×CH(<u>C</u>H_3)$_2$-Dipp), 18.4 (s, CH_3-Cymene).

^{19}F{^{1}H} NMR (376 MHz, dichloromethane-*d*$_2$): *δ* [ppm] = −129.7 (br s, 6 F, *o*-F), −162.8 (t, *J* = 20.4 Hz, 3 F, *p*-F), −167.5 (t, *J* = 18.5 Hz, 6 F, *m*-F).

^{31}P{^{1}H} NMR (162 MHz, dichloromethane-*d*$_2$): *δ* [ppm] = 25.6 (s).

HRMS (ES−) *m/z* calcd for [(WCA-IDipp)AgCl]$^-$ ($C_{45}H_{35}BN_2F_{15}ClAg$): 1041.1393, found 1041.1379.

HRMS (ES+) *m/z* calcd for [(*p*-cymene)Ru(PPh_3)]$^+$ ($C_{28}H_{28}PRu$): 497.0972, found 497.0971.

2.8 [(WCA-IDipp)Cu(*μ*-Cl_2)Ru(PPh_3)(*η*^{6}-*p*-cymene)] (6b)

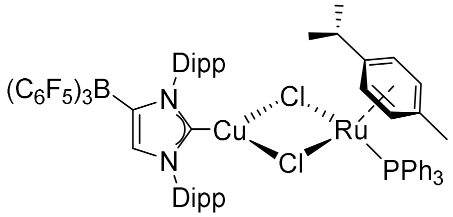

A brown one-neck flask is charged with [(WCA-IDipp)Cu(toluene)] (**5b**, 39.0 mg, 0.037 mmol, 1.0 equiv.) and suspended in toluene (4 mL). Ruthenium complex [(*η*^{6}-*p*-cymene)RuCl_2(PPh_3)] (21.0 mg, 0.037 mmol, 1.0 equiv.) is added and the mixture is stirred for 16 h and then filtered through a pad of Celite®. The solvent is removed under high vacuum and the crude product recrystallized from DCM/*n*-hexane to obtain product **6b** as orange-red crystals (55.0 mg, 0.036 mmol, 97%).

elemental analysis (%) calc. for $C_{74}H_{68}BCl_2CuF_{15}N_2PRu\cdot CH_2Cl_2$: C 54.98, H 4.12, N 1.73; found C 55.41, H 4.10, N 1.87.

^{1}H NMR (400 MHz, dichloromethane-*d*$_2$): *δ* [ppm] = 7.51−7.36 (m, 11 H, PPh_3 + *p*-Dipp), 7.32 (d, $^3J_{H,H}$ = 7.7 Hz, 2 H, *m*-Dipp), 7.26 (dt, $^3J_{H,H}$ = 8.0, 2.3 Hz, 6 H, PPh_3), 7.10 (d, $^3J_{H,H}$ = 7.7 Hz, 2 H, *m*-Dipp), 6.32 (br s, 1 H, C<u>H</u>=CB), 4.77 (d, $^3J_{H,H}$ = 6.0 Hz, 2 H, CH-Cymene), 4.66 (d, $^3J_{H,H}$ = 6.1 Hz, 2 H, CH-Cymene), 2.97 (sept, $^3J_{H,H}$ = 6.2 Hz, 2 H, C<u>H</u>(CH_3)$_2$-Dipp), 2.78 (sept, $^3J_{H,H}$ = 6.3 Hz, 2 H, C<u>H</u>(CH_3)$_2$-Dipp), 2.23 (sept, $^3J_{H,H}$ = 6.9 Hz, 1 H, C<u>H</u>(CH_3)$_2$-Cymene), 1.81 (s, 3 H, CH_3-Cymene), 1.34 (d, $^3J_{H,H}$ =

6.8 Hz, 6 H, $CH(C\underline{H}_3)_2$-Dipp), 1.24 (d, $^3J_{H,H}$ = 6.8 Hz, 6 H, $CH(C\underline{H}_3)_2$-Dipp), 1.10 (d, $^3J_{H,H}$ = 6.8 Hz, 6 H, $CH(C\underline{H}_3)_2$-Dipp), 0.96 (d, $^3J_{H,H}$ = 6.9 Hz, 6 H, $CH(C\underline{H}_3)_2$-Cymene), 0.89 (d, $^3J_{H,H}$ = 6.7 Hz, 6 H, $CH(C\underline{H}_3)_2$-Dipp).

$^{11}B\{^1H\}$ NMR (128 MHz, dichloromethane-d_2): δ [ppm] = −15.96 (s).

$^{13}C\{^1H\}$ NMR (101 MHz, dichloromethane-d_2): δ [ppm] = 180.9 (s, NCN), 150.5 (m, aryl-C_6F_5), 148.1 (m, CH=$\underline{C}$B), 148.0 (s, 2×*o*-Dipp), 147.5 (s, 2×*o*-Dipp), 140.2 (m, aryl-C_6F_5), 138.9 (s, *i*-Dipp), 138.4−137.8 (m, aryl-C_6F_5), 136.9 (s, *i*-Dipp), 135.8 (m, aryl-C_6F_5), 134.2 (d, $^2J_{C,P}$ = 9.7 Hz, 6×*o*-PPh_3), 132.8 (d, $^1J_{C,P}$ = 47.0 Hz, 3×*i*-PPh_3), 131.4 (d, $^4J_{C,P}$ = 2.6 Hz, 3×*p*-PPh_3), 130.4 (br s, $\underline{C}$H=CB), 129.8 (s, *p*-Dipp), 129.2 (s, *p*-Dipp), 128.9 (d, $^3J_{C,P}$ = 10.2 Hz, 6×*m*-PPh_3), 124.0 (s, 2×*m*-Dipp), 123.2 (s, 2×*m*-Dipp), 108.3 (s, C_{Ar}-Cymene), 97.5 (s, C_{Ar}-Cymene), 90.8 (d, $J_{C,P}$ = 4.8 Hz, 2×CH_{Ar}-Cymene), 87.4 (d, $J_{C,P}$ = 5.2 Hz, 2×CH_{Ar}-Cymene), 31.2 (s, $\underline{C}H(CH_3)_2$-Cymene), 28.7 (s, 2×$\underline{C}H(CH_3)_2$-Dipp), 28.3 (s, 2×$\underline{C}H(CH_3)_2$-Dipp), 27.5 (s, 2×$CH(\underline{C}H_3)_2$-Dipp), 25.2 (s, 2×$CH(\underline{C}H_3)_2$-Dipp), 24.3 (s, 2×$CH(\underline{C}H_3)_2$-Dipp), 22.4 (s, 2×$CH(\underline{C}H_3)_2$-Cymene), 22.4 (s, 2×$CH(\underline{C}H_3)_2$-Dipp), 18.3 (s, CH_3-Cymene).

$^{19}F\{^1H\}$ NMR (376 MHz, dichloromethane-d_2): δ [ppm] = −129.4 (br s, 6 F, *o*-F), −162.9 (t, J = 20.4 Hz, 3 F, *p*-F), −167.5 (m, 6 F, *m*-F).

$^{31}P\{^1H\}$ NMR (162 MHz, dichloromethane-d_2): δ [ppm] = 24.9 (s).

HRMS (ES−) *m/z* calcd for $[(WCA\text{-}IDipp)CuCl]^-$ ($C_{45}H_{35}BClCuF_{15}N_2$): 997.16383, found 997.16598.

HRMS (ES−) *m/z* calcd for $[((WCA\text{-}IDipp)Cu)_2(\mu\text{-}Cl)]^-$ ($C_{90}H_{70}B_2ClCu_2F_{30}N_4$): 1961.35700, found 1961.35881.

HRMS (ES+) *m/z* calcd for $[(p\text{-}cymene)RuCl(PPh_3)]^+$ ($C_{28}H_{28}PRu$): 533.07389, found 533.07422.

2.9 [(WCA-IDipp)Ag(μ-I_2)Ru(PPh_3)(η^6-*p*-cymene)] (7)

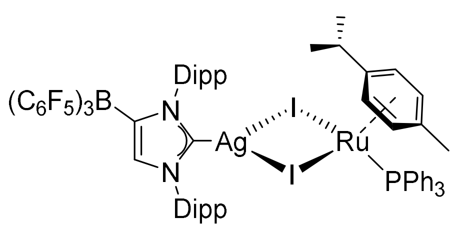

A brown one-neck flask is charged with [(WCA-IDipp)Ag(toluene)] (**5a** ,33.0 mg, 0.03 mmol, 1.0 equiv.) and dissolved in DCM (1 mL). Ruthenium complex [(η^6-*p*-cymene)RuI_2(PPh_3)] (22.5 mg, 0.03 mmol, 1.0 equiv.) is added and the mixture is stirred for 1 h and then filtered through a pad of Celite®. The solvent is removed under high vacuum and the crude product recrystallized from DCM/*n*-hexane at −37 °C to obtain product **7** as red crystals (46.0 mg, 0.026 mmol, 87%).

elemental analysis (%) calc. for: $C_{73}H_{68}AgBF_{15}I_2N_2PRu$: C 49.85, H 3.67, N 1.59; found C 50.07, H 3.75, N 1.70.

^{1}H NMR (400 MHz, dichloromethane-*d*$_2$): δ [ppm] = 7.47−7.34 (m, 10 H, PPh_3 + *p*-Dipp), 7.31−7.26 (m, 7 H, PPh_3 + *p*-Dipp), 7.19 (d, $^3J_{H,H}$ = 7.7 Hz, 2 H, *m*-Dipp), 7.01 (d, $^3J_{H,H}$ = 7.7 Hz, 2 H, *m*-Dipp), 6.32 (d, $^3J_{H,F}$ = 2.2 Hz, 2 H, C$\underline{\text{H}}$=CB), 5.15 (d, $^3J_{H,H}$ = 6.1 Hz, 2 H, CH-Cymene), 4.83 (d, $^3J_{H,H}$ = 6.3 Hz, 2 H, CH-Cymene), 2.98−2.82 (m, 3 H, C$\underline{\text{H}}$(CH_3)$_2$-Dipp + C$\underline{\text{H}}$(CH_3)$_2$-Cymene), 2.65 (sept, $^3J_{H,H}$ = 6.9 Hz, 2 H, C$\underline{\text{H}}$(CH_3)$_2$-Dipp), 1.88 (s, 3 H, CH_3-Cymene), 1.22 (d, $^3J_{H,H}$ = 6.9 Hz, 6 H, CH(C$\underline{\text{H}}_3$)$_2$-Dipp), 1.17 (d, $^3J_{H,H}$ = 6.8 Hz, 6 H, CH(C$\underline{\text{H}}_3$)$_2$-Dipp), 1.08 (d, $^3J_{H,H}$ = 6.8 Hz, 12 H, CH(C$\underline{\text{H}}_3$)$_2$-Dipp), 0.89 (d, $^3J_{H,H}$ = 6.7 Hz, 6 H, CH(C$\underline{\text{H}}_3$)$_2$-Cymene).

^{11}B{^{1}H} NMR (128 MHz, dichloromethane-*d*$_2$): δ [ppm] = −16.03 (s).

^{13}C{^{1}H} NMR (101 MHz, dichloromethane-*d*$_2$): δ [ppm] = 182.8 (dd, $^1J_{C,Ag}$ = 289.9, 253.5 Hz, NCN), 150.5 (m, aryl-C_6F_5), 148.1 (m, CH=$\underline{\text{C}}$B), 147.5 (s, 2×*o*-Dipp), 146.8 (s, 2×*o*-Dipp), 140.3 (m, aryl-C_6F_5), 138.7 (s, *i*-Dipp), 138.1 (m, aryl-C_6F_5), 136.5 (s, *i*-Dipp), 135.8 (m, aryl-C_6F_5), 135.1 (d, $^2J_{C,P}$ = 9.4 Hz, 6×*o*-PPh_3), 135.0 (d, $^1J_{C,P}$ = 48.0 Hz, 3×*i*-PPh_3), 131.3 (d, $^4J_{C,P}$ = 2.6 Hz, 3×*p*-PPh_3), 130.3 (br s, $\underline{\text{C}}$H=CB), 129.8 (s, *p*-Dipp), 129.3 (s, *p*-Dipp), 128.5 (d, $^3J_{C,P}$ = 10.0 Hz, 6×*m*-PPh_3), 124.0 (s, 2×*m*-Dipp), 123.3 (s, 2×*m*-Dipp), 114.7 (d, $^2J_{C,P}$ = 3.9 Hz, C_{Ar}-Cymene), 101.1 (s, C_{Ar}-Cymene), 90.2 (d, $^2J_{C,P}$ = 2.4 Hz, 2×CH_{Ar}-Cymene), 88.4 (d, $^2J_{C,P}$ = 4.6 Hz, 2×CH_{Ar}-Cymene), 32.0 (s, $\underline{\text{C}}$H(CH_3)$_2$-Cymene), 28.5 (s, 2×$\underline{\text{C}}$H(CH_3)$_2$-Dipp), 28.3 (s, 2×CH($\underline{\text{C}}$H$_3$)$_2$-Dipp), 28.2 (s, 2×$\underline{\text{C}}$H(CH_3)$_2$-Dipp), 25.0 (s, 2×CH($\underline{\text{C}}$H$_3$)$_2$-Dipp), 24.8 (s, 2×CH($\underline{\text{C}}$H$_3$)$_2$-Dipp), 23.0 (s, 2×CH($\underline{\text{C}}$H$_3$)$_2$-Dipp), 22.3 (s, 2×CH($\underline{\text{C}}$H$_3$)$_2$-Cymene), 19.4 (s, CH_3-Cymene).

^{19}F{^{1}H} NMR (376 MHz, dichloromethane-d_2): δ [ppm] = −129.4 (br s, 6 F, *o*-F), −163.0 (t, J = 20.4 Hz, 3 F, *p*-F), −167.6 (m, 6 F, *m*-F).

^{31}P{^{1}H} NMR (162 MHz, dichloromethane-d_2): δ [ppm] = 25.4 (s).

HRMS (ES−) *m/z* calcd for [(WCA-IDipp)AgI]$^-$ ($C_{45}H_{35}AgBF_{15}IN_2$): 1135.07460, found 1135.07703.

HRMS (ES+) *m/z* calcd for [(*p*-cymene)RuI(PPh$_3$)]$^+$ ($C_{28}H_{29}IPRu$): 625.00950, found 625.00955.

2.10 [(WCA-IDipp)Cu(Cl)][(Ru(η^6-*p*-cymene))$_2$(μ-Cl$_3$)]

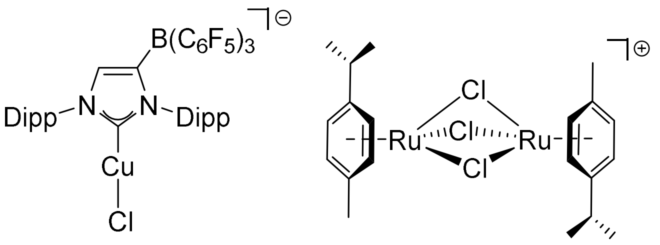

A one-neck flask is charged with [Li(THF)$_4$][(WCA-IDipp)CuCl] (**3**, 51.8 mg, 0.040 mmol, 1.0 equiv.) and dissolved in DCM (2 mL). Ruthenium complex [(η^6-*p*-cymene)RuCl$_2$]$_2$ (24.5 mg, 0.040 mmol, 1.0 equiv.) is added and the resulting turbid orange solution is stirred for 2 h and then filtered through a pad of Celite®. The solvent is removed under high vacuum and the crude product recrystallized from DCM/*n*-hexane at −37 °C to obtain the product as orange crystals (59 mg, 0.037 mmol, 94%).

The structural composition of this compound is supported by a preliminary X-ray diffraction analysis (see Figure S10). Because of low crystal quality, no data sufficient for publication could be obtained.

elemental analysis (%) calc. for $C_{65}H_{63}BCl_4CuF_{15}N_2Ru_2$: C 49.55, H 4.03, N 1.78; found C 49.40, H 3.84, N 1.85.

^{1}H NMR (400 MHz, dichloromethane-d_2): δ [ppm] = 7.41 (t, $^3J_{H,H}$ = 7.8 Hz, 1 H, *p*-Dipp), 7.32 (t, $^3J_{H,H}$ = 7.7 Hz, 1 H, *p*-Dipp), 7.23 (d, $^3J_{H,H}$ = 7.7 Hz, 2 H, *m*-Dipp), 7.03 (d, $^3J_{H,H}$ = 7.7 Hz, 2 H, *m*-Dipp), 6.27 (br s, 1 H, C<u>H</u>=CB), 5.60 (d, $^3J_{H,H}$ = 6.3 Hz, 4 H, CH-Cymene), 5.41 (d, $^3J_{H,H}$ = 6.2 Hz, 4 H, CH-Cymene), 2.92 (sept, $^3J_{H,H}$ = 6.8 Hz, 2 H, C<u>H</u>(CH$_3$)$_2$-Dipp), 2.81−2.68 (m, 4 H, C<u>H</u>(CH$_3$)$_2$-Dipp + C<u>H</u>(CH$_3$)$_2$-Cymene), 2.18 (s, 6 H, CH$_3$-Cymene), 1.28 (d, $^3J_{H,H}$ = 6.9 Hz, 12 H, CH(C<u>H</u>$_3$)$_2$-Cymene), 1.25−1.23 (m, 12

H, 2×$CH(C\underline{H}_3)_2$-Dipp), 1.12 (d, $^3J_{H,H}$ = 6.9 Hz, 6 H, $CH(C\underline{H}_3)_2$-Dipp), 0.93 (d, $^3J_{H,H}$ = 6.7 Hz, 6 H, $CH(C\underline{H}_3)_2$-Dipp).

^{11}B{^{1}H} NMR (128 MHz, dichloromethane-d_2): δ [ppm] = −16.11 (s).

^{13}C{^{1}H} NMR (101 MHz, dichloromethane-d_2): δ [ppm] = 179.4 (s, NCN), 150.5 (m, aryl-C_6F_5), 148.1 (m, CH=$\underline{C}$B), 147.9 (s, 2×*o*-Dipp), 146.8 (s, 2×*o*-Dipp), 140.2 (m, aryl-C_6F_5), 138.2 (m, aryl-C_6F_5), 137.8 (s, *i*-Dipp), 136.2 (s, *i*-Dipp), 135.8 (m, aryl-C_6F_5), 130.1 (br s, $\underline{C}$H=CB), 129.9 (s, *p*-Dipp), 129.4 (s, *p*-Dipp), 124.1 (s, 2×*m*-Dipp), 123.1 (s, 2×*m*-Dipp), 102.5 (s, 2×C_{Ar}-Cymene), 97.7 (s, 2×C_{Ar}-Cymene), 79.3 (s, 4×CH_{Ar}-Cymene), 78.6 (s, 4×CH_{Ar}-Cymene), 32.0 (s, 2×$\underline{C}H(CH_3)_2$-Cymene), 28.6 (s, 2×$\underline{C}H(CH_3)_2$-Dipp), 28.2 (s, 2×$\underline{C}H(CH_3)_2$-Dipp), 27.5 (s, 2×$CH(\underline{C}H_3)_2$-Dipp), 25.0 (s, 2×$CH(\underline{C}H_3)_2$-Dipp), 24.2 (s, 2×$CH(\underline{C}H_3)_2$-Dipp), 22.3 (s, 4×$CH(\underline{C}H_3)_2$-Cymene), 22.1 (s, 2×$CH(\underline{C}H_3)_2$-Dipp), 19.2 (s, 2×CH_3-Cymene).

^{19}F{^{1}H} NMR (376 MHz, dichloromethane-d_2): δ [ppm] = −129.2 (br s, 6 F, *o*-F), −163.0 (t, J = 20.4 Hz, 3 F, *p*-F), −167.6 (m, 6 F, *m*-F).

3 Spectra

3.1 [Li(THF)$_4$][(WCA-IDipp)$_2$Ag] (2)

^{1}H NMR (500 MHz, THF-d_8) of [Li(THF)$_4$][(WCA-IDipp)$_2$Ag] (**2**):

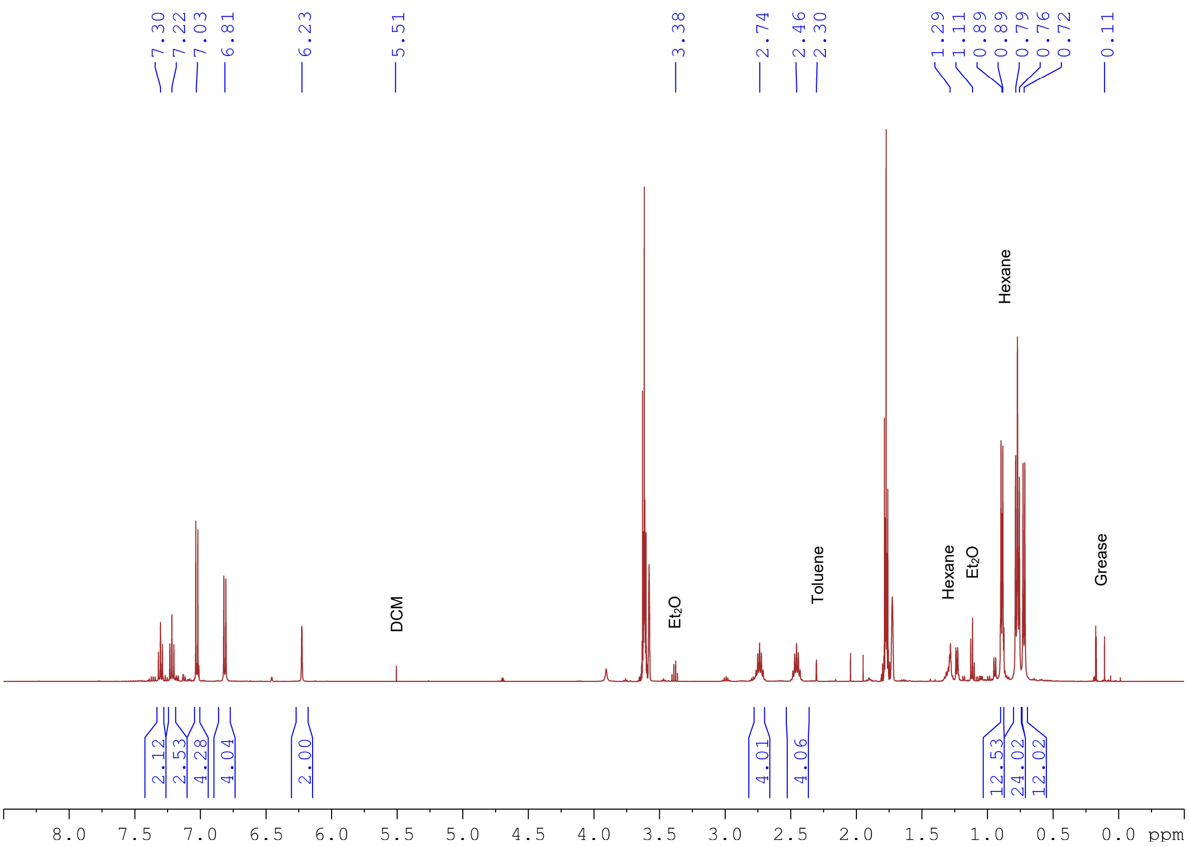

^{13}C NMR (125 MHz, THF-*d*$_8$) of [Li(THF)$_4$][(WCA-IDipp)$_2$Ag] (**2**):

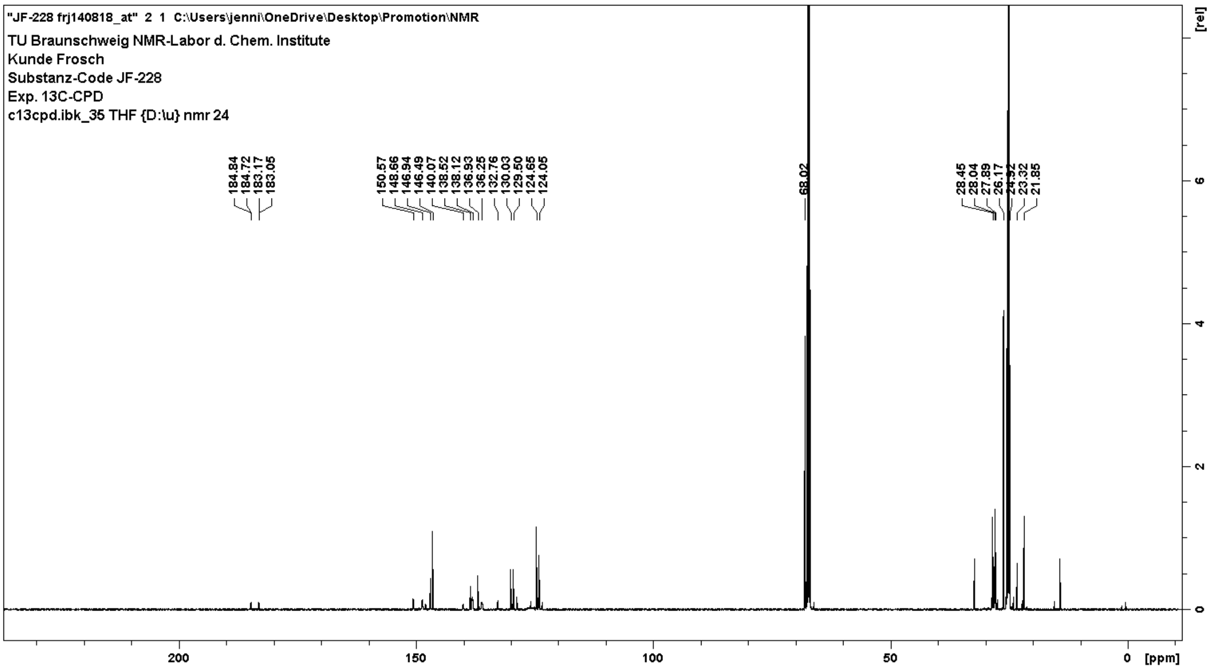

^{13}C NMR (125 MHz, THF-*d*$_8$) of [Li(THF)$_4$][(WCA-IDipp)$_2$Ag] (**2**) in the region of the aryl ^{13}C atoms:

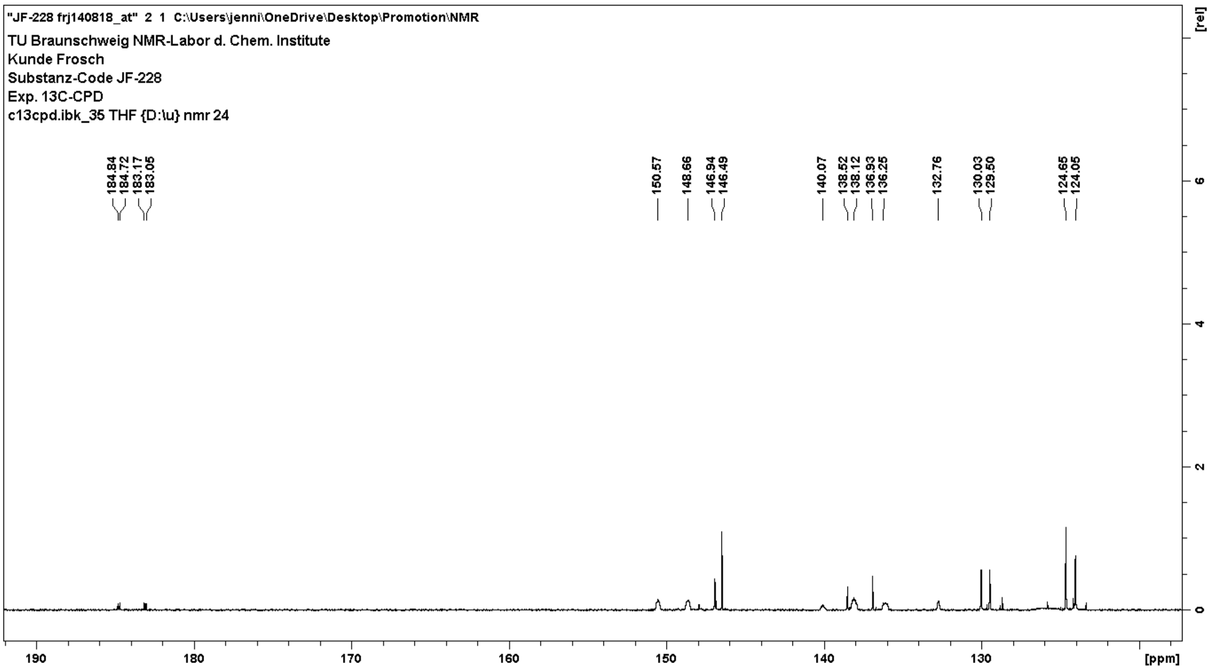

^{13}C NMR (125 MHz, THF-d_8) of [Li(THF)$_4$][(WCA-IDipp)$_2$Ag] (**2**) in the region of the alkyl ^{13}C atoms:

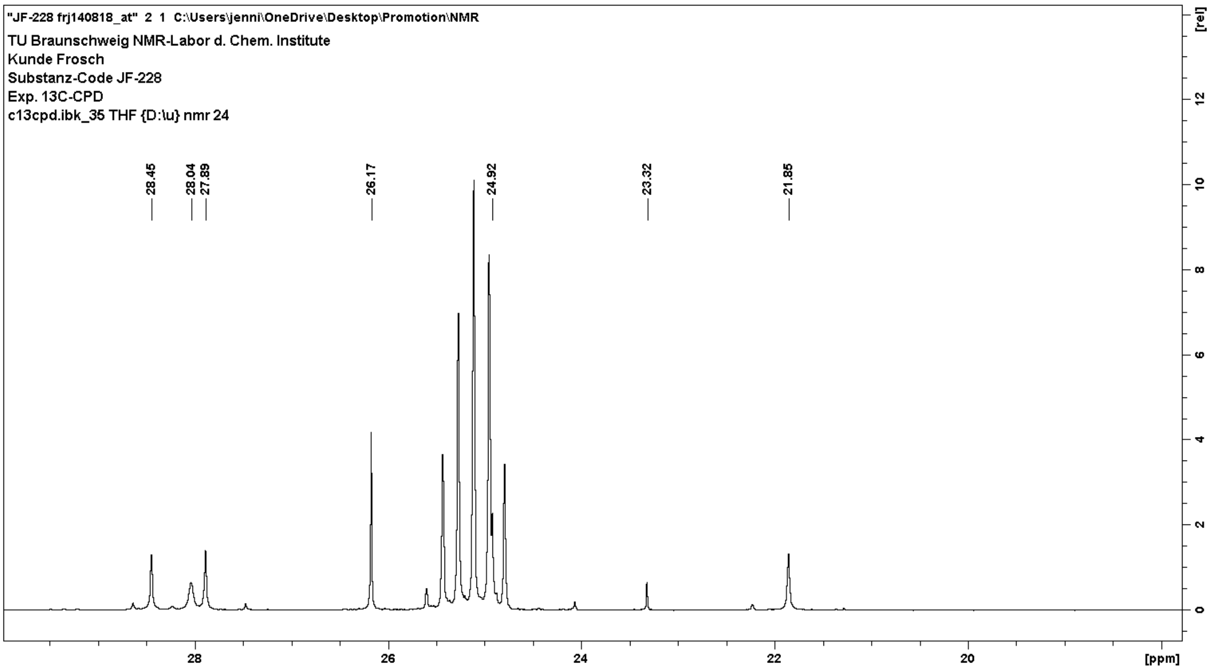

^{11}B{^{1}H} NMR (128 MHz, THF-d_8) of [Li(THF)$_4$][(WCA-IDipp)$_2$Ag] (**2**):

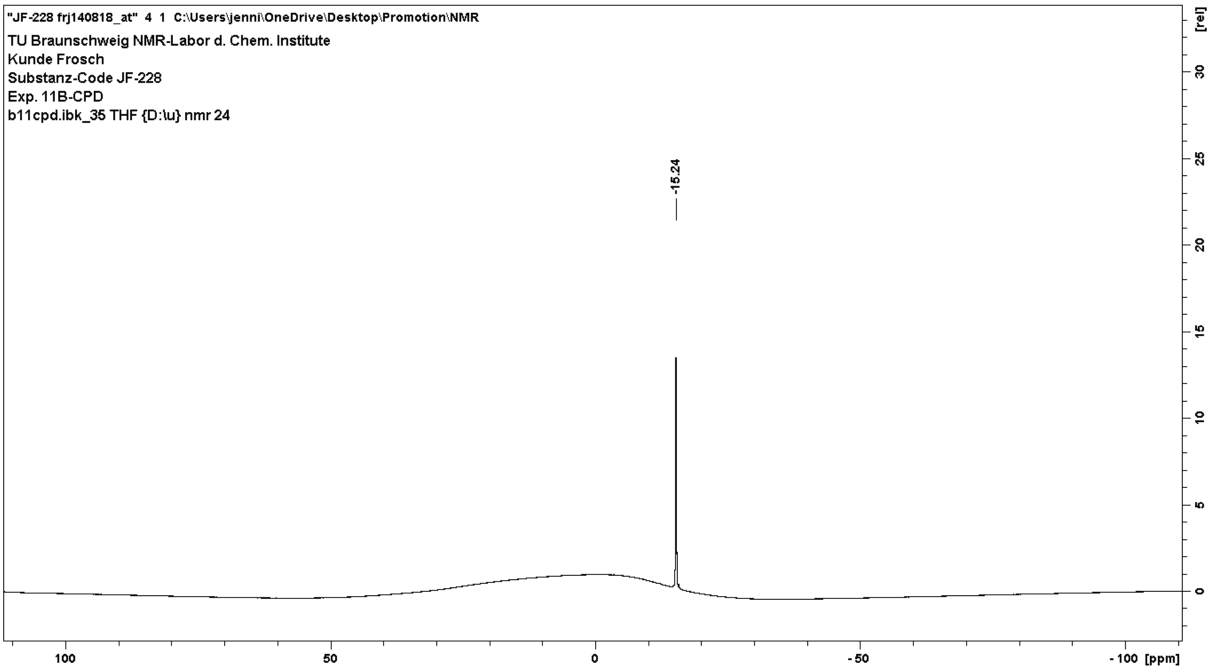

$^{19}F\{^1H\}$ NMR (376 MHz, THF-d_8) of [Li(THF)$_4$][(WCA-IDipp)$_2$Ag] (**2**):

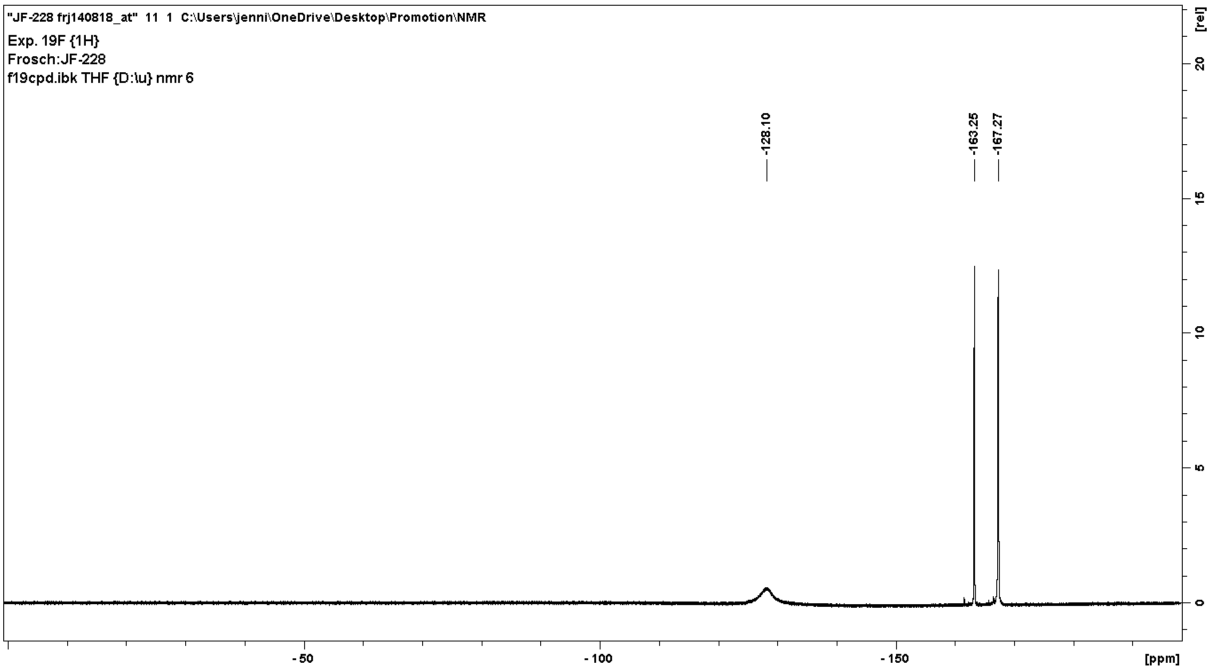

3.2 [Li(THF)$_n$][(WCA-IDipp)CuCl] (3)

^1H NMR (400 MHz, dichloromethane-d_2) of [Li(THF)$_n$][(WCA-IDipp)CuCl] (**3**):

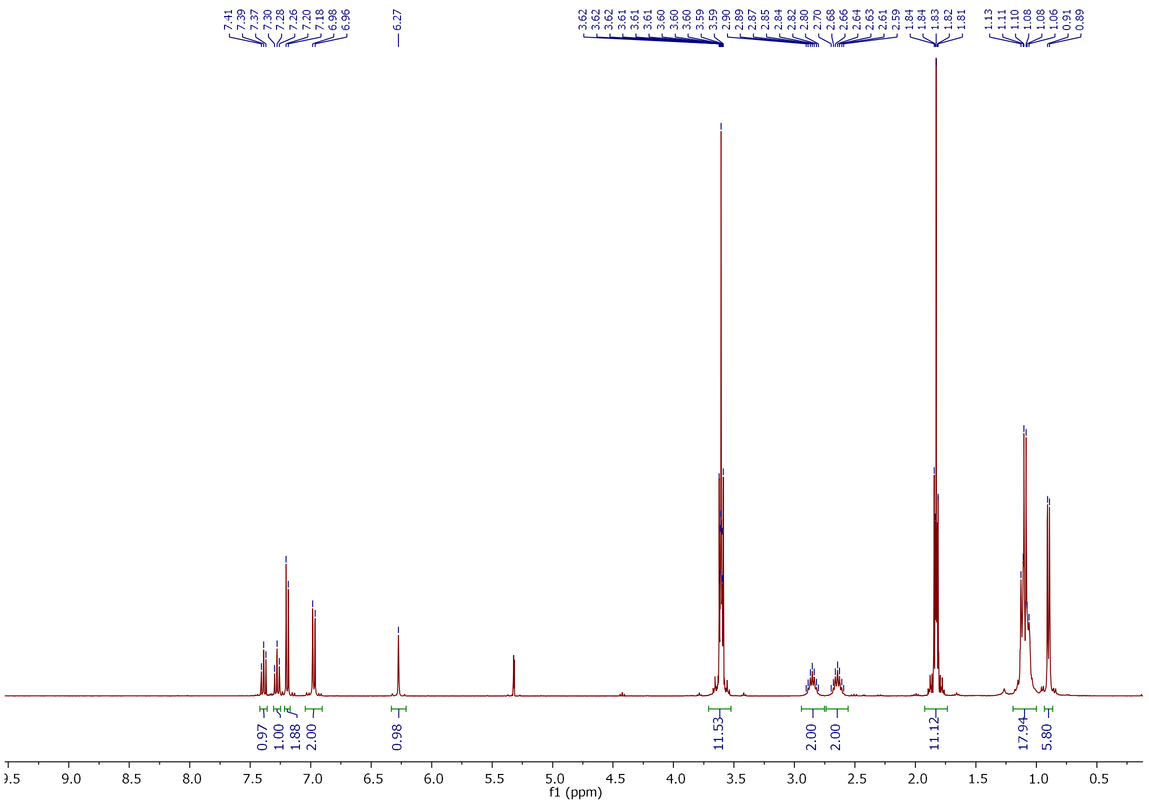

$^{13}C\{^1H\}$ NMR (101 MHz, dichloromethane-d_2) of [Li(THF)$_n$][(WCA-IDipp)CuCl] (**3**):

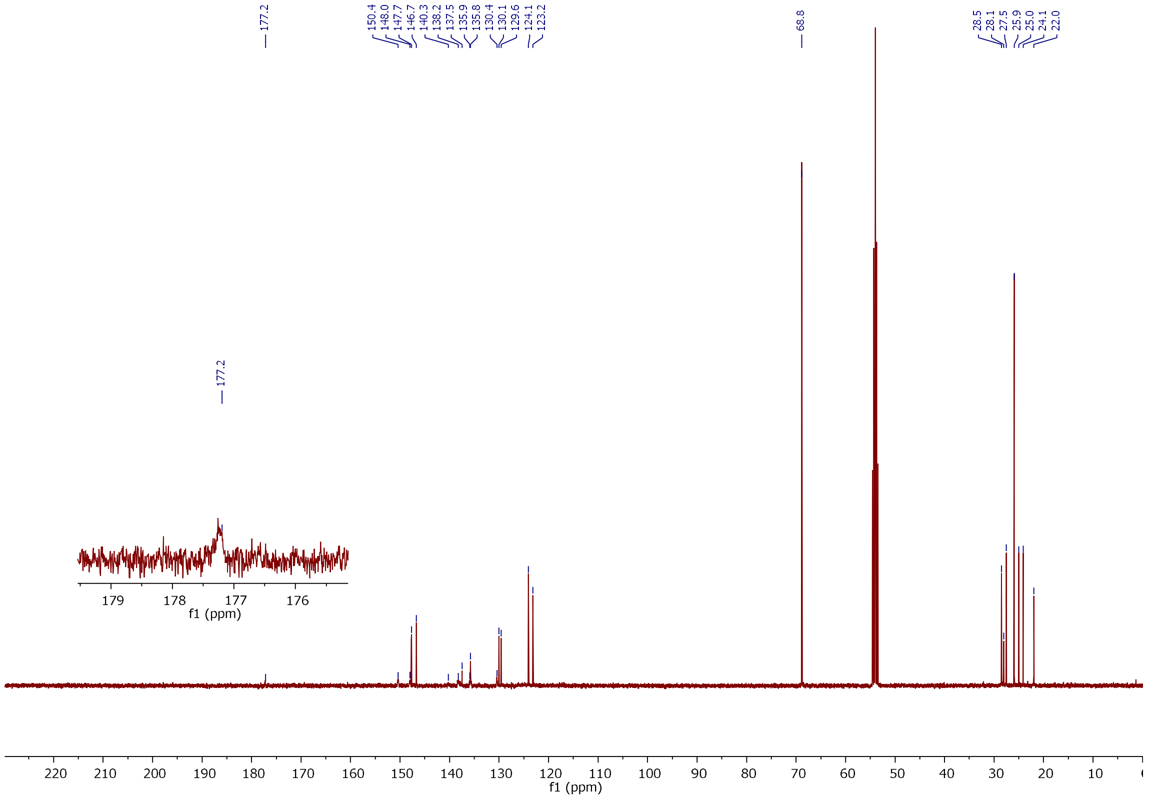

$^{11}B\{^1H\}$ NMR (128 MHz, dichloromethane-d_2) of [Li(THF)$_n$][(WCA-IDipp)CuCl] (**3**):

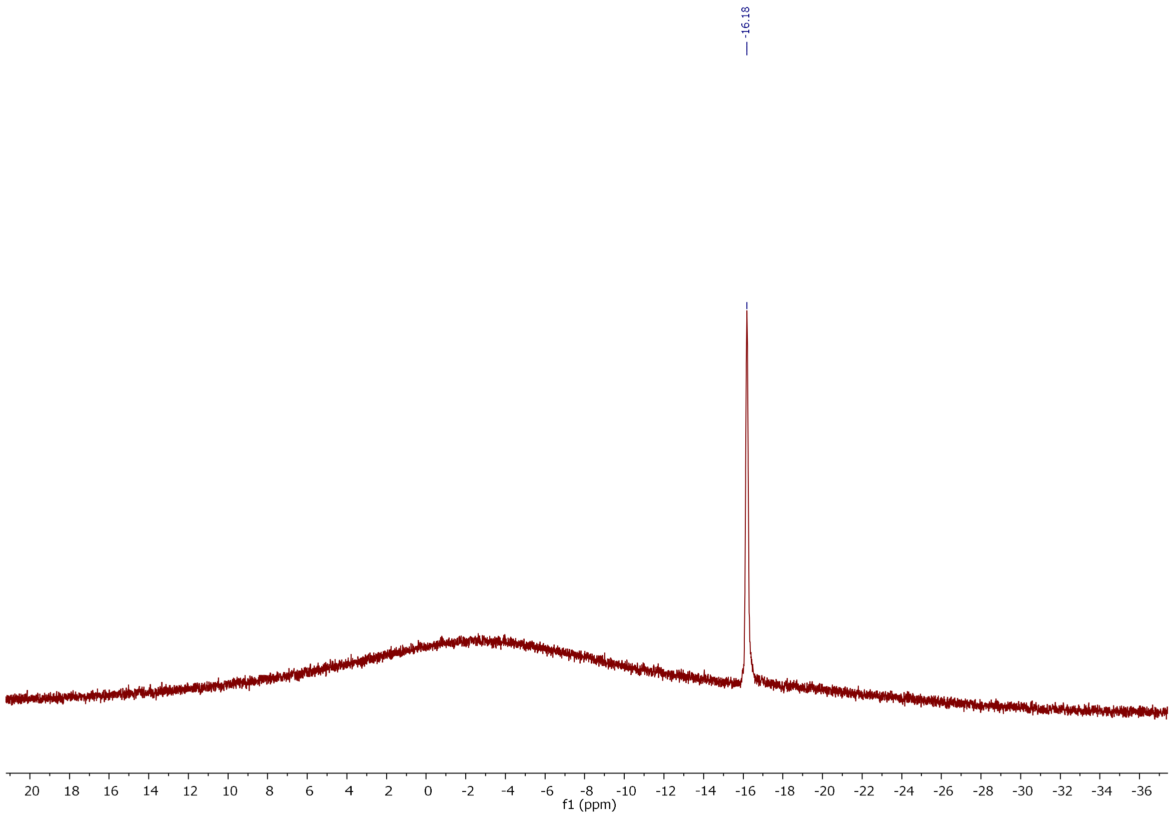

$^{19}F\{^{1}H\}$ NMR (376 MHz, dichloromethane-d_2) of [Li(THF)$_n$][(WCA-IDipp)CuCl] (**3**):

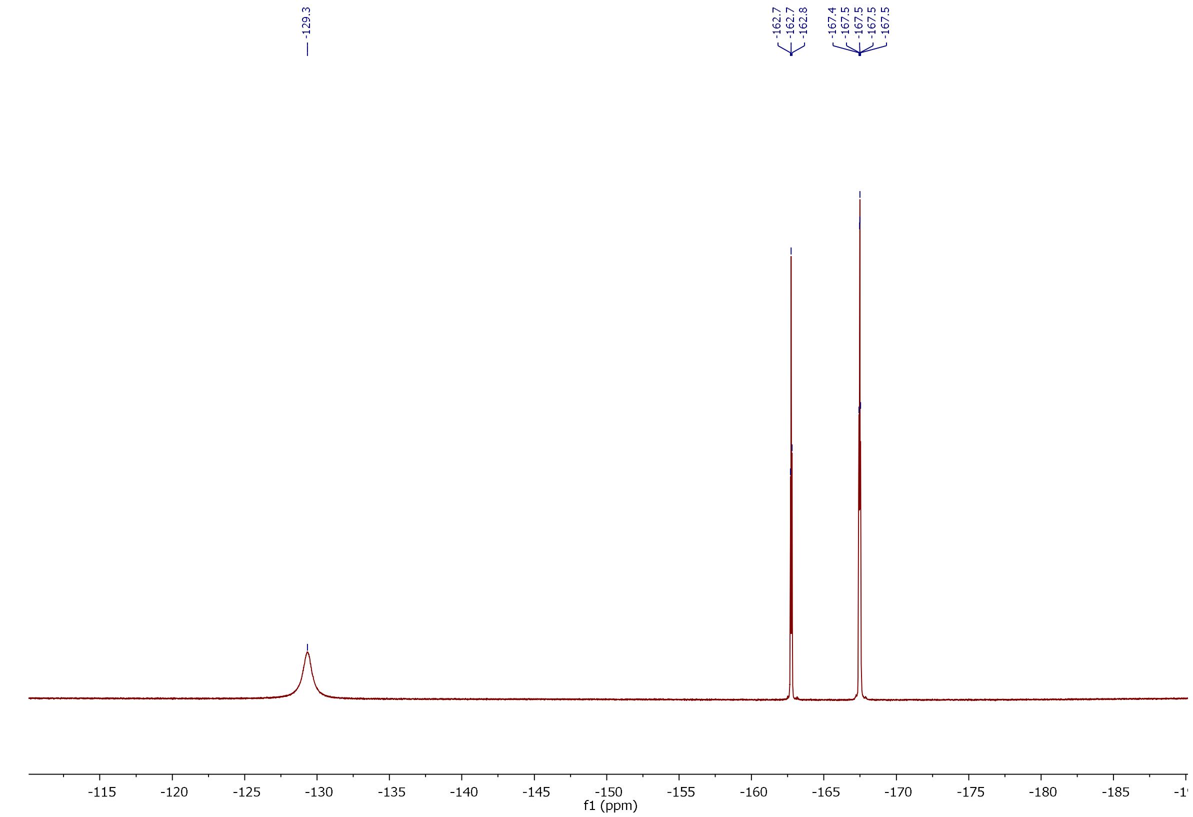

3.3 [(WCA-IDipp)Ag(PPh$_3$)] (4a)

^{1}H NMR (400 MHz, dichloromethane-d_2) of [(WCA-IDipp)Ag(PPh$_3$)] (**4a**):

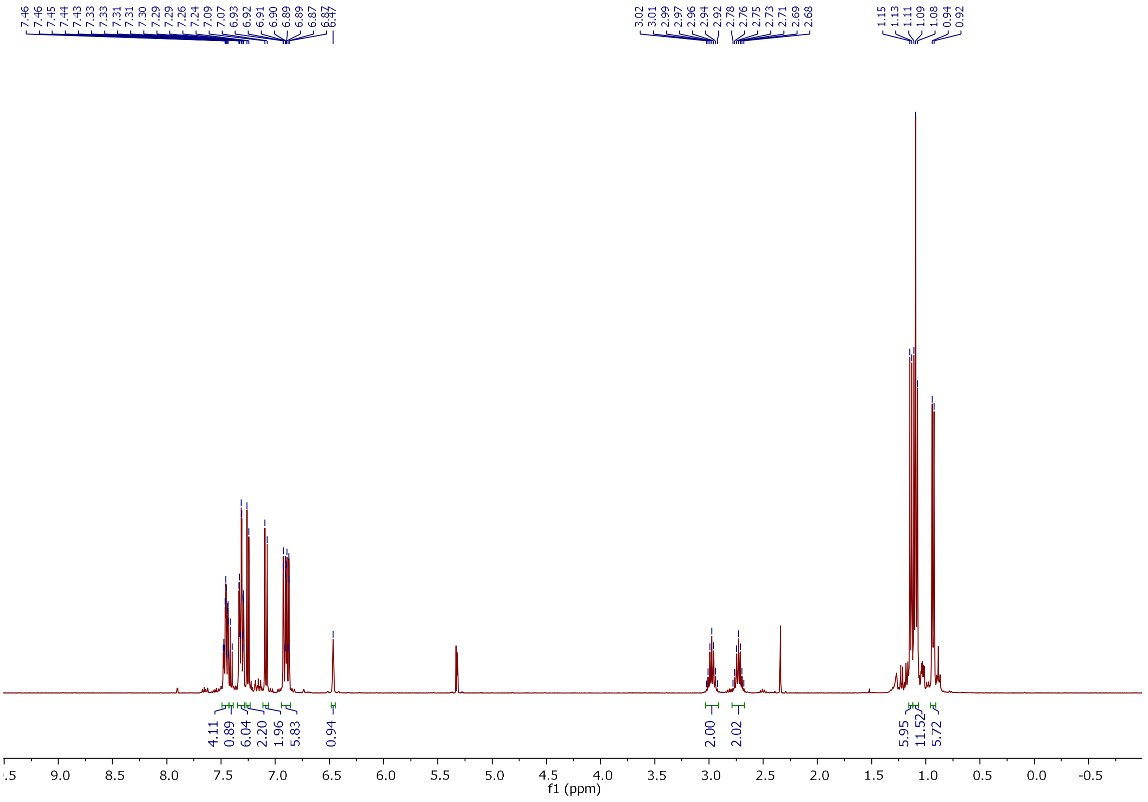

$^{13}C\{^{1}H\}$ NMR (101 MHz, dichloromethane-d_2) of [(WCA-IDipp)Ag(PPh$_3$)] (**4a**):

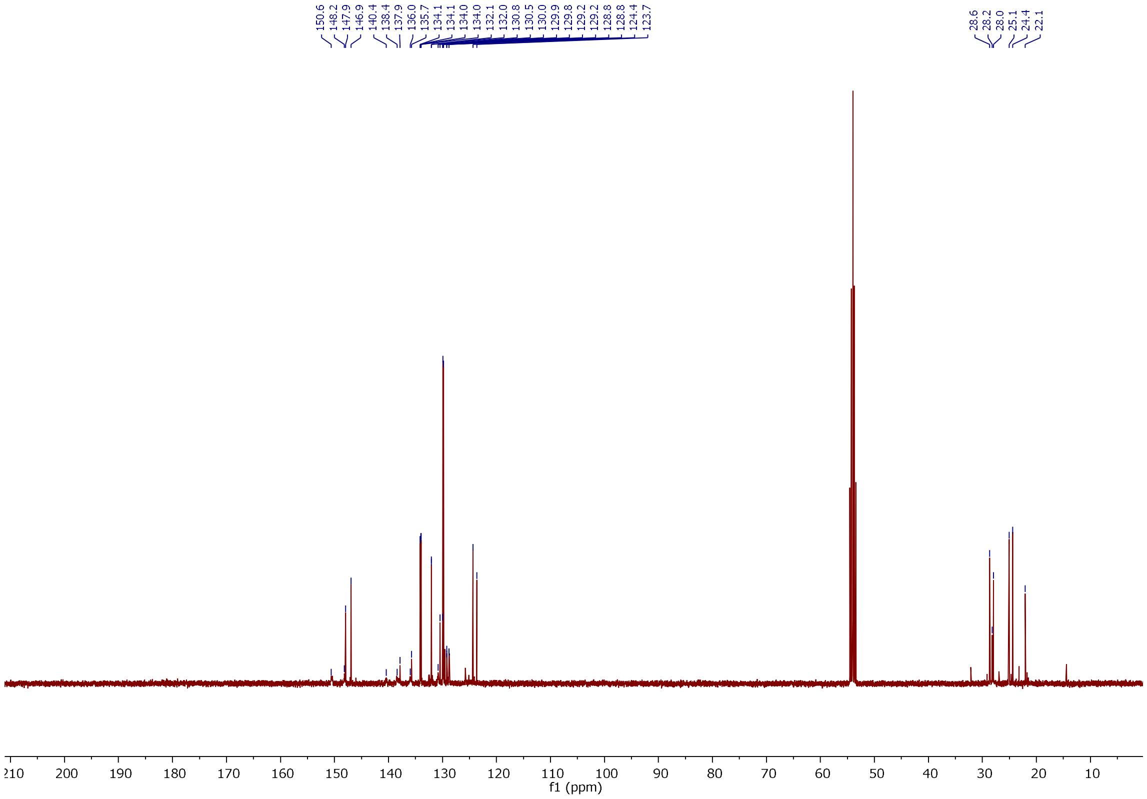

$^{11}B\{^{1}H\}$ NMR (128 MHz, dichloromethane-d_2) of [(WCA-IDipp)Ag(PPh$_3$)] (**4a**):

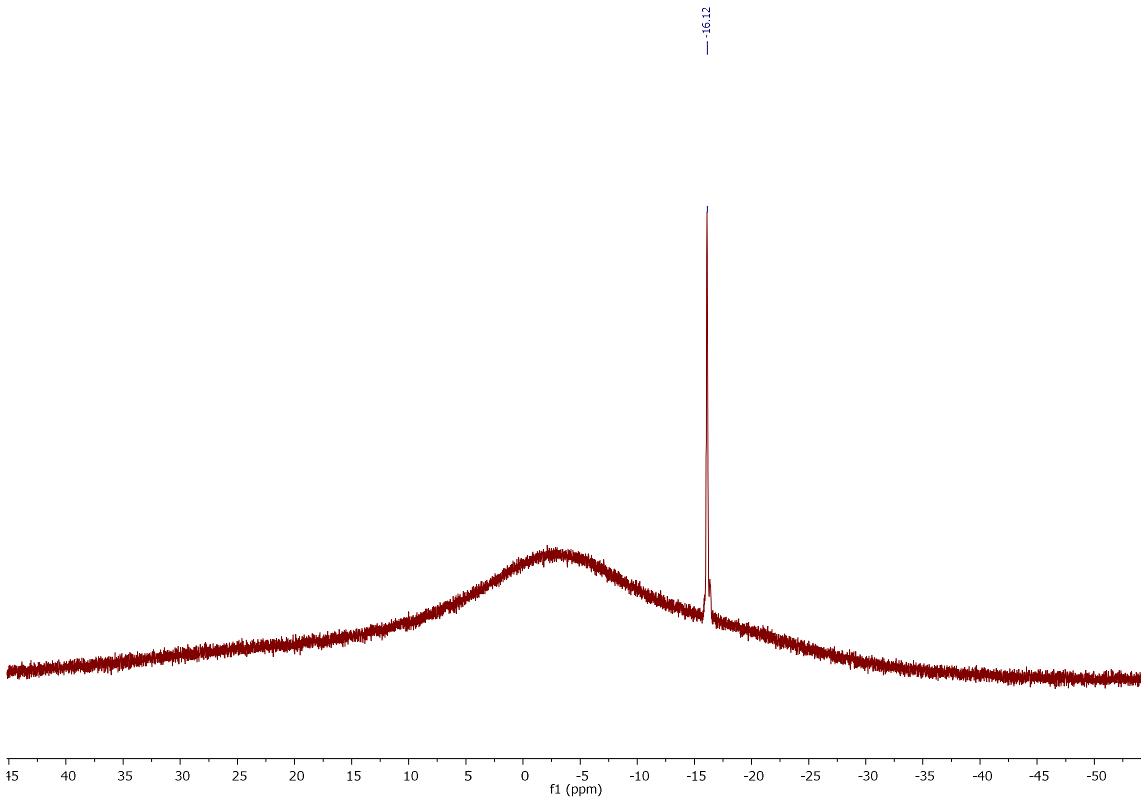

$^{19}F\{^1H\}$ NMR (376 MHz, dichloromethane-d_2) of [(WCA-IDipp)Ag(PPh3)] (**4a**):

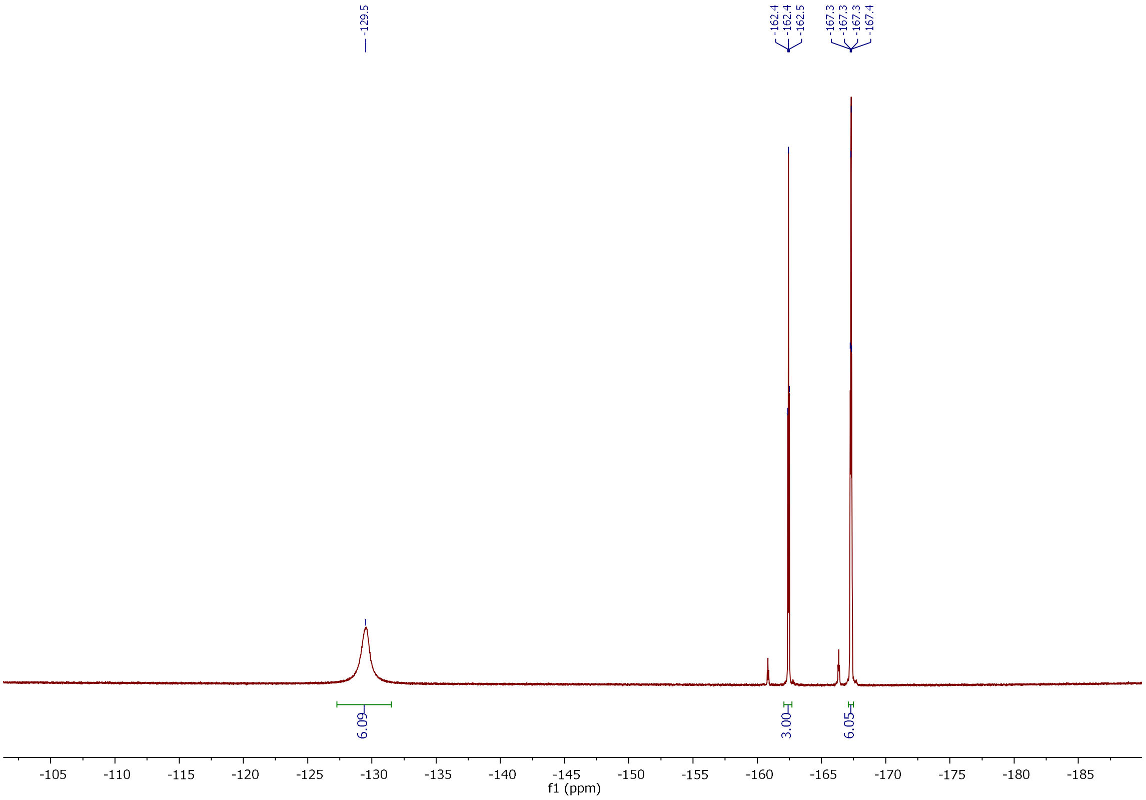

$^{31}P\{^1H\}$ NMR (162 MHz, dichloromethane-d_2) of [(WCA-IDipp)Ag(PPh3)] (**4a**):

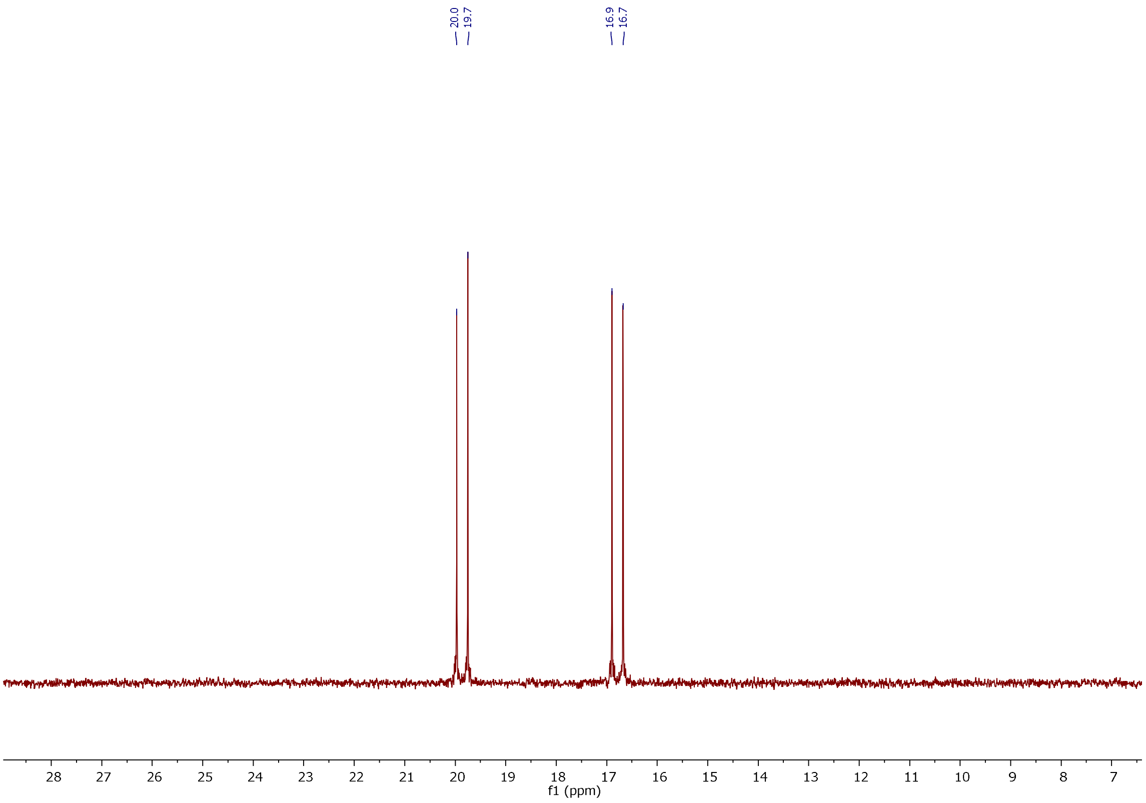

3.4 [(WCA-IDipp)Cu(PPh$_3$)] (4b)

^{1}H NMR (500 MHz, THF-*d*$_8$) of [(WCA-IDipp)Cu(PPh$_3$)] (**4b**):

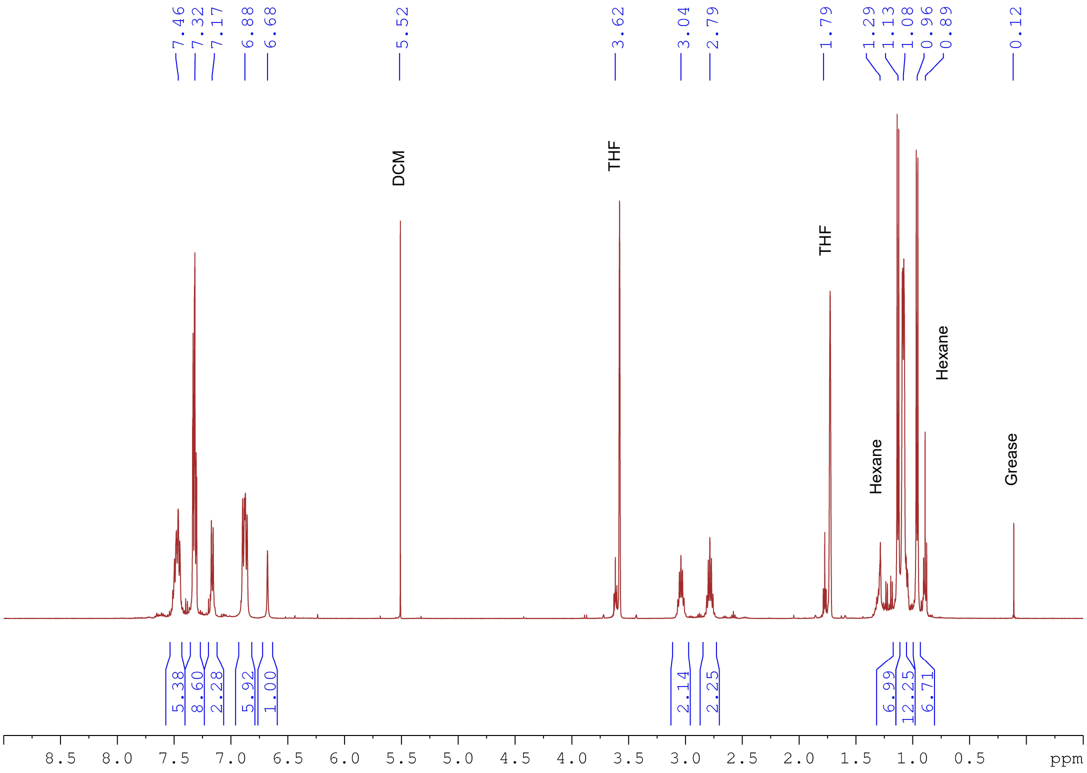

^{13}C{^{1}H} NMR (125 MHz, THF-*d*$_8$) of [(WCA-IDipp)Cu(PPh$_3$)] (**4b**):

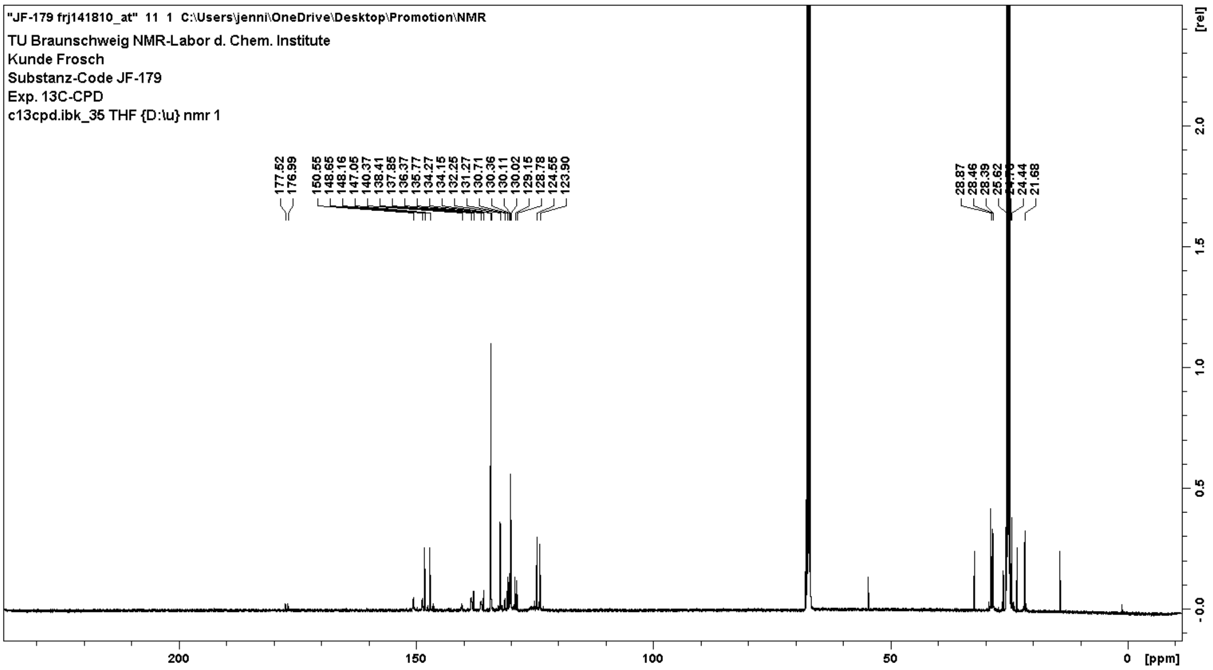

^{13}**C NMR** (125 MHz, THF-*d*$_8$) of [(WCA-IDipp)Cu(PPh$_3$)] (**4b**) in the region of the aryl ^{13}C atoms:

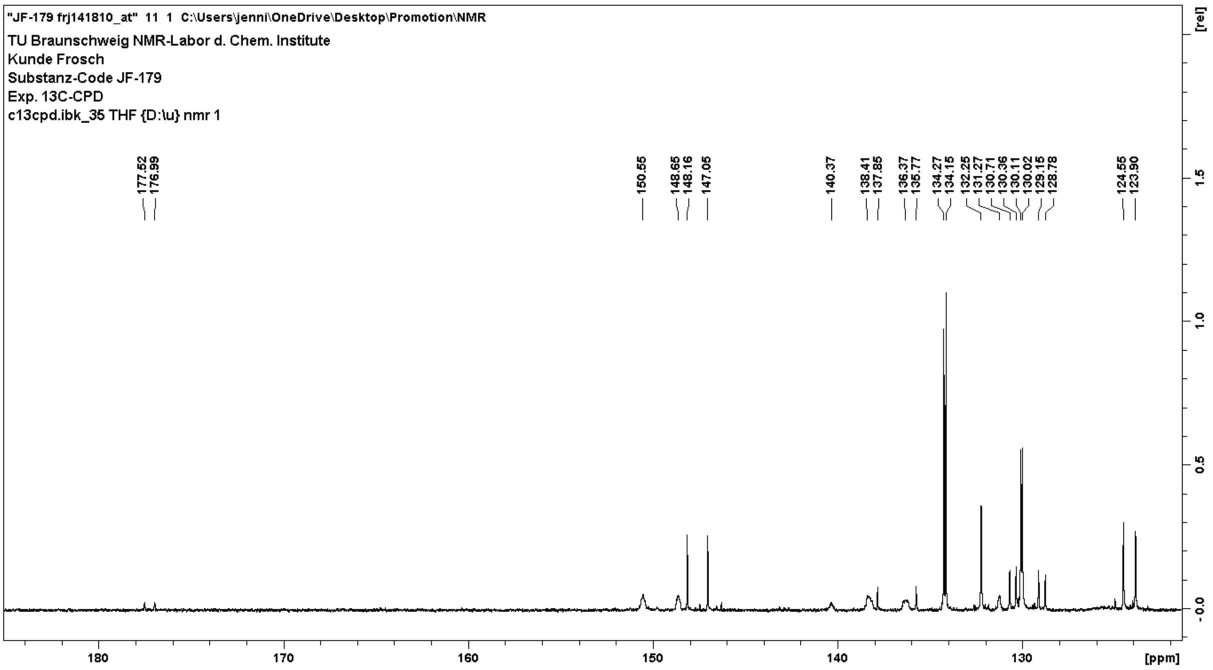

^{13}**C NMR** (125 MHz, THF-*d*$_8$) of [(WCA-IDipp)Cu(PPh$_3$)] (**4b**) in the region of the alkyl ^{13}C atoms:

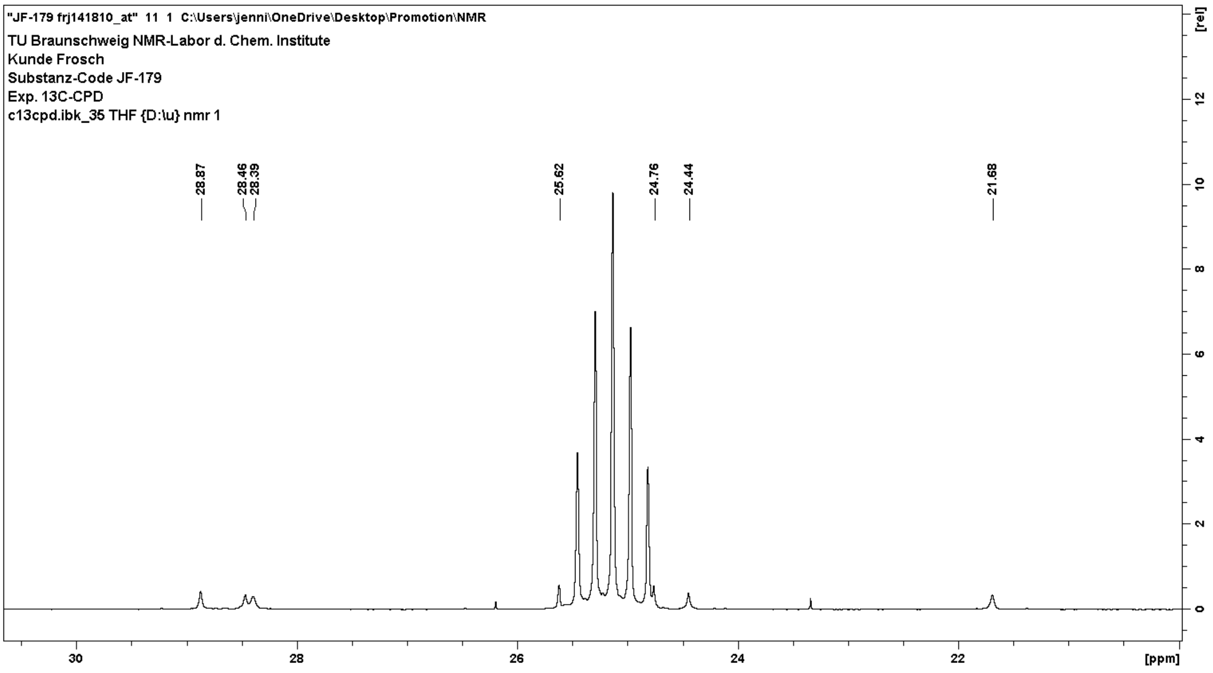

$^{11}B\{^1H\}$ **NMR** (160 MHz, THF-d_8) of [(WCA-IDipp)Cu(PPh_3)] (**4b**):

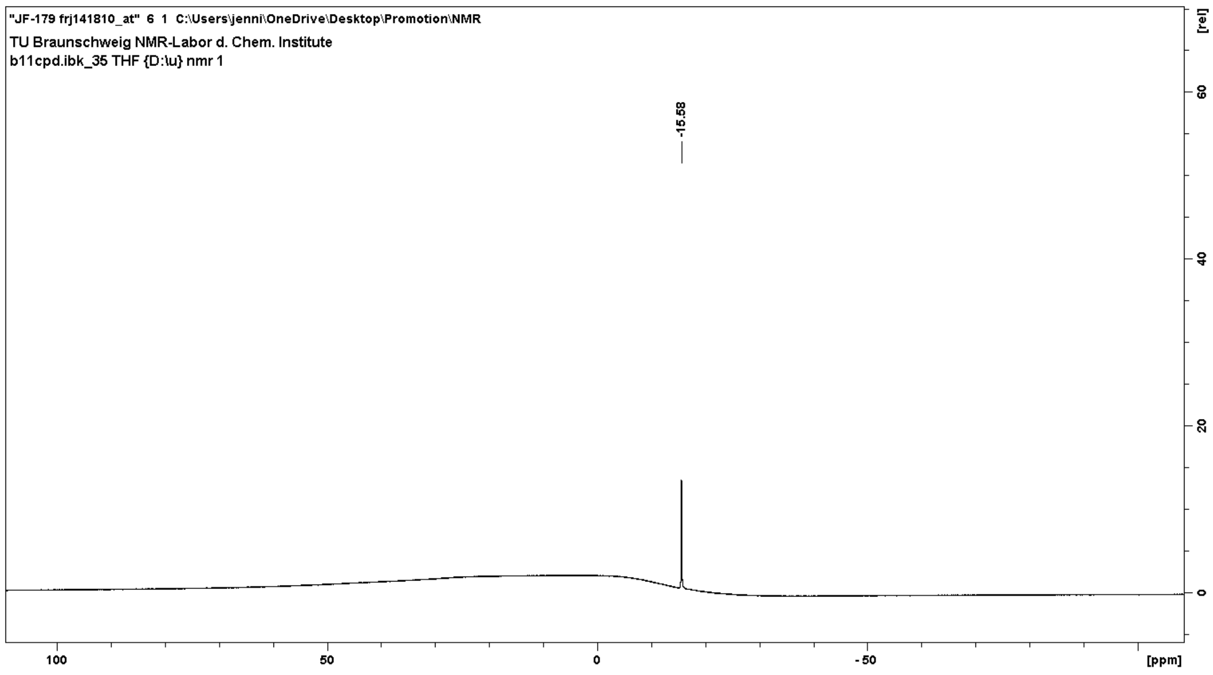

$^{19}F\{^1H\}$ **NMR** (470 MHz, THF-d_8) of [(WCA-IDipp)Cu(PPh_3)] (**4b**):

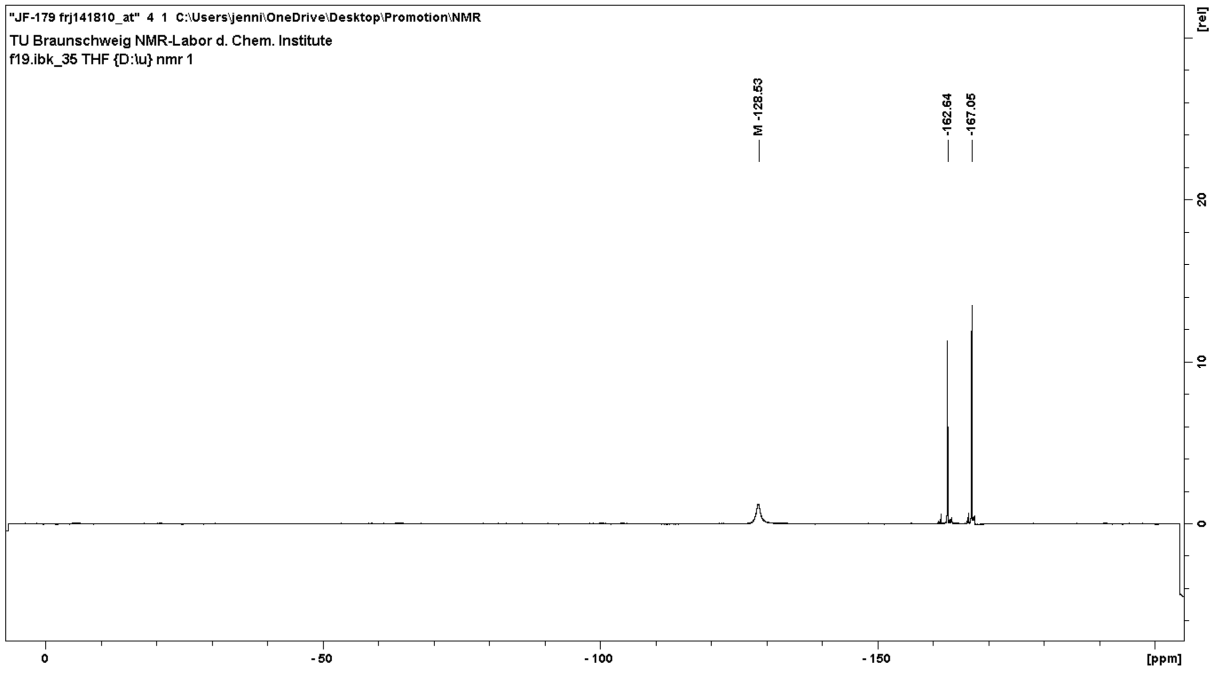

$^{31}P\{^{1}H\}$ NMR (202 MHz, THF-*d*8) of [(WCA-IDipp)Cu(PPh3)] (**4b**):

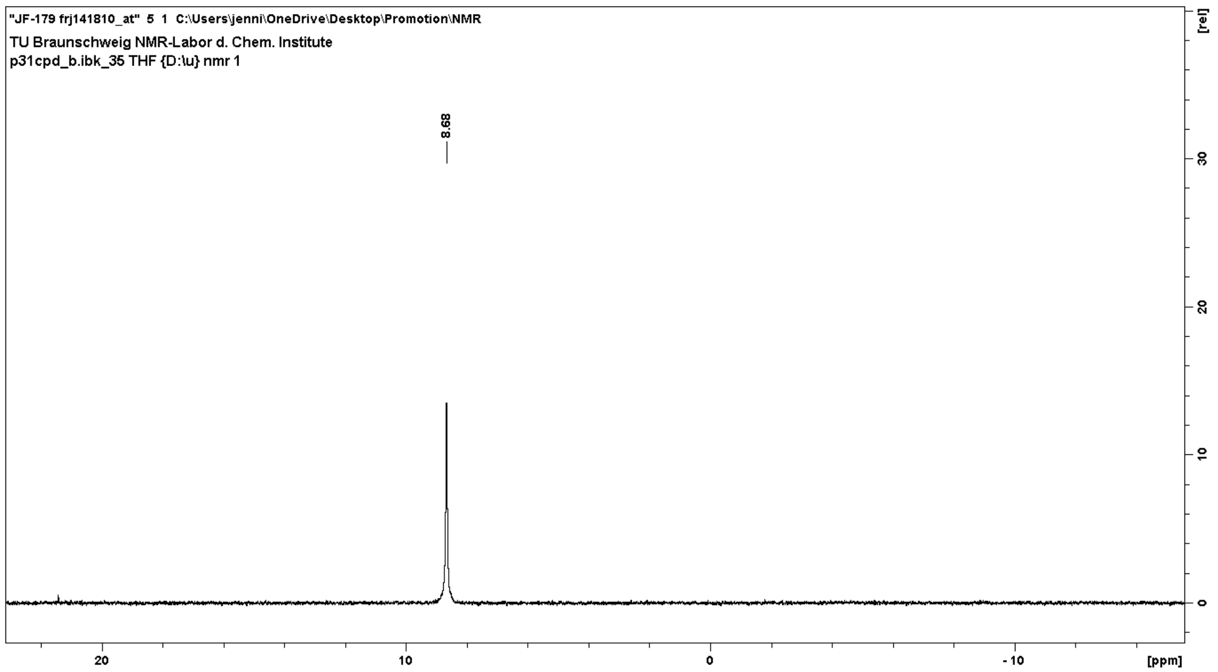

3.5 [(WCA-IDipp)Ag(η^2-Tol)] (5a)

^{1}H NMR (400 MHz, THF-*d*8) of [(WCA-IDipp)Ag(η^2-Tol)] (**5a**):

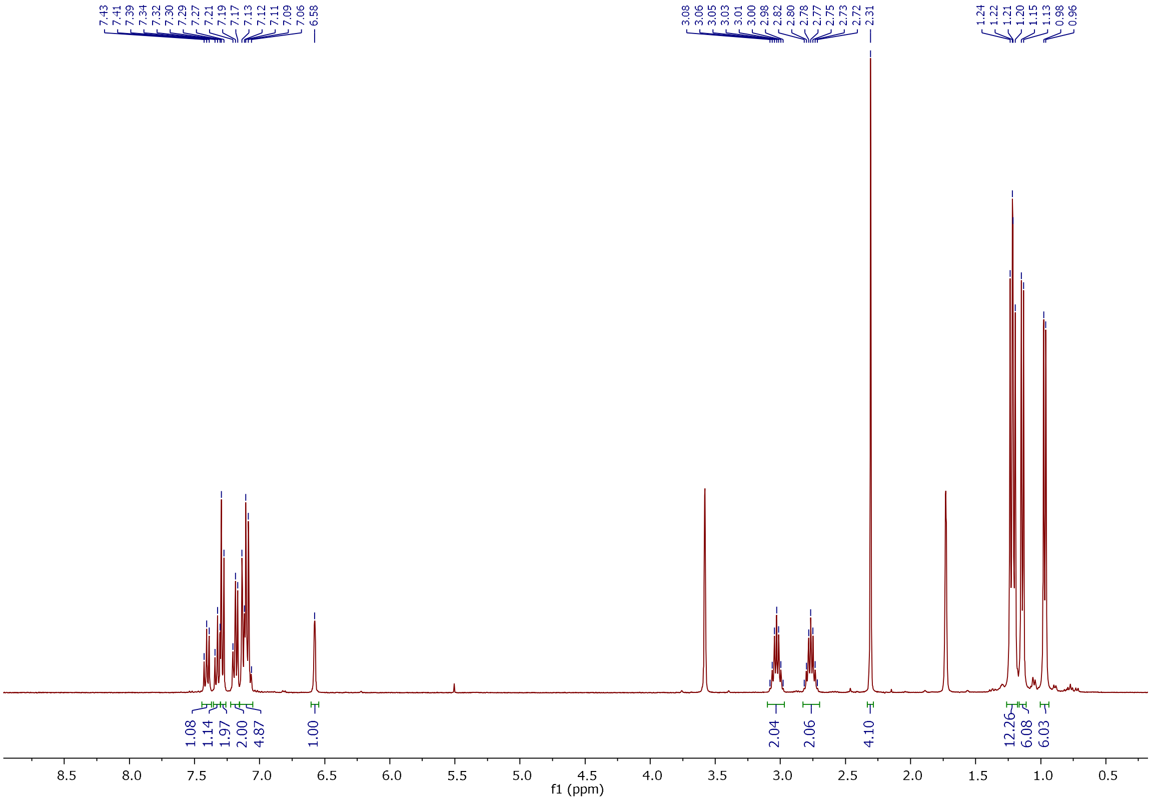

^{13}C{^{1}H} NMR (101 MHz, THF-*d*8) of [(WCA-IDipp)Ag(η^2-Tol)] (**5a**):

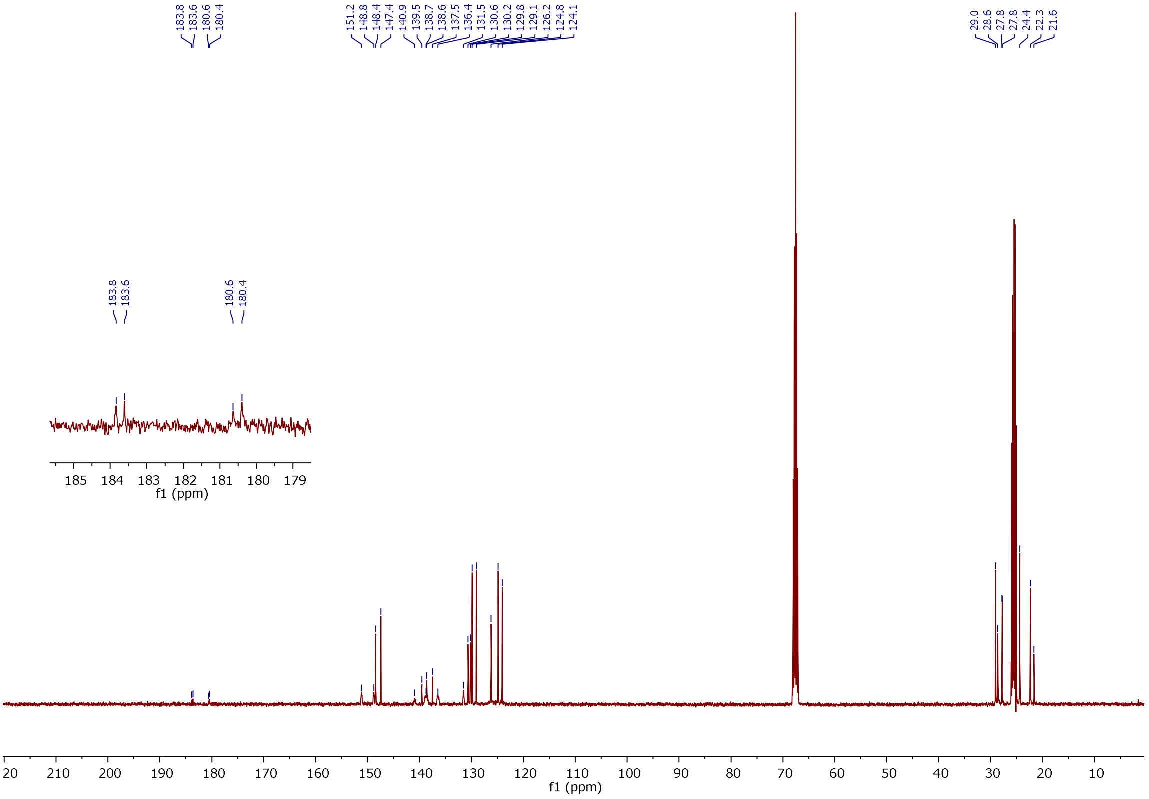

^{11}B{^{1}H} NMR (128 MHz, dichloromethane-*d*2) of [(WCA-IDipp)Ag(η^2-Tol)] (**5a**):

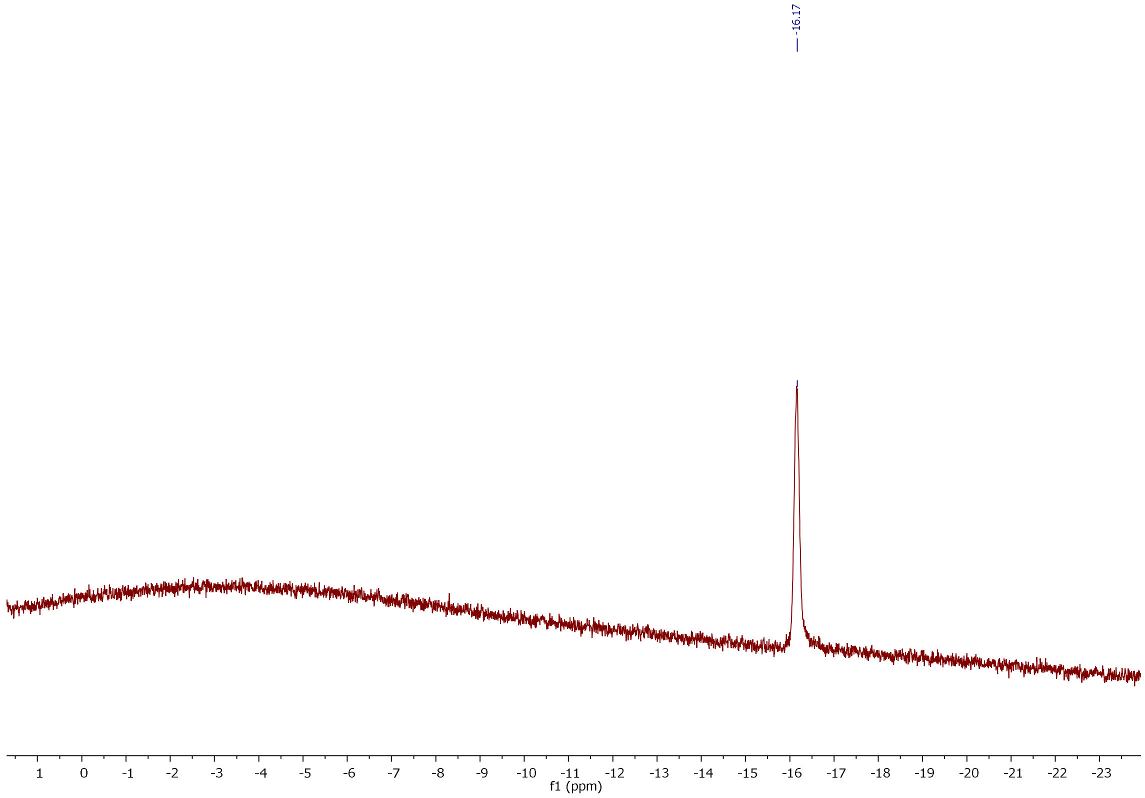

$^{19}F\{^1H\}$ NMR (376 MHz, THF-d_8) of [(WCA-IDipp)Ag(η^2-Tol)] (**5a**):

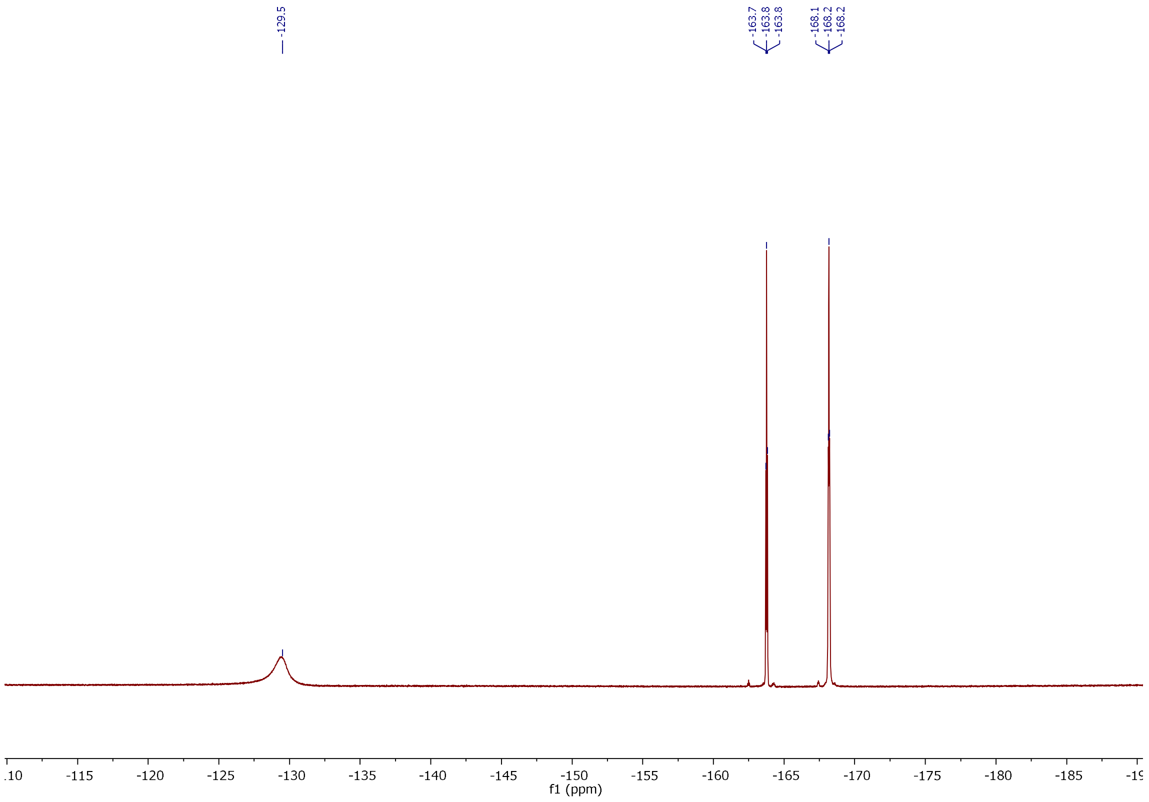

3.6 [(WCA-IDipp)Cu(η^2-Tol)] (5b)

^1H NMR (400 MHz, dichloromethane-d_2) of [(WCA-IDipp)Cu(η^2-Tol)] (**5b**):

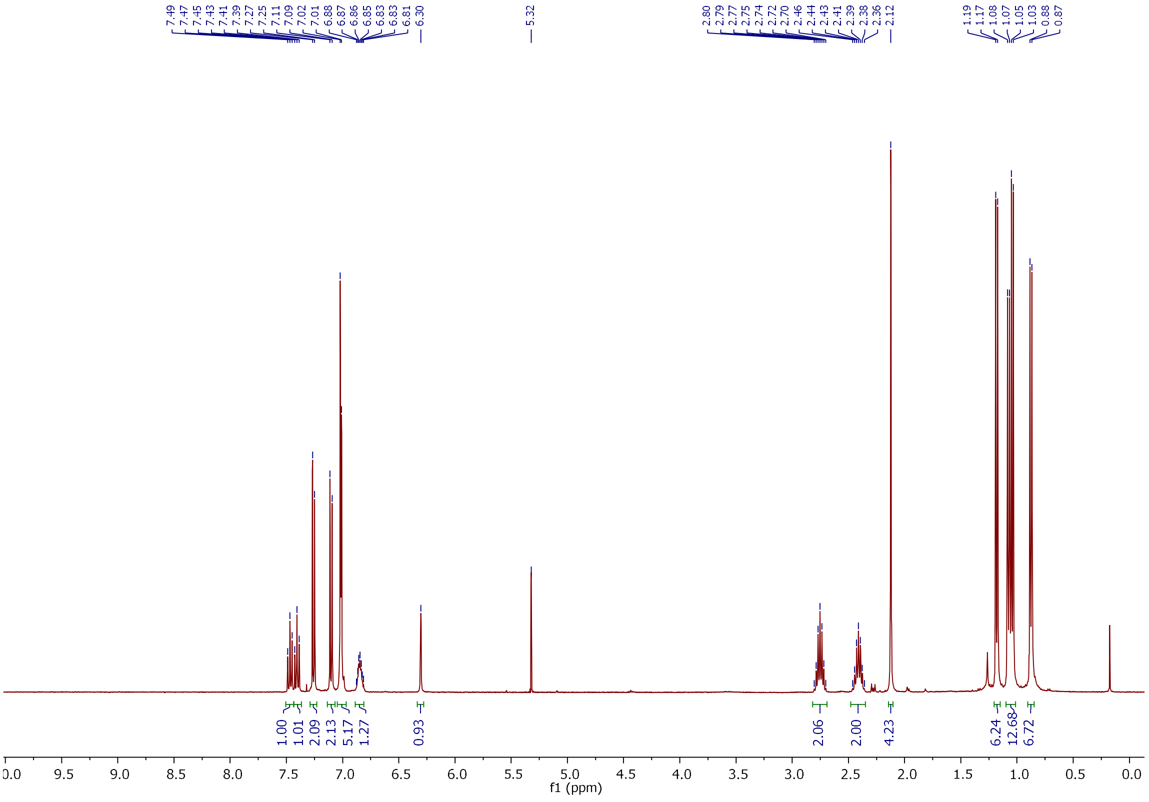

$^{13}C\{^1H\}$ NMR (101 MHz, dichloromethane-d_2) of [(WCA-IDipp)Cu(η^2-Tol)] (**5b**):

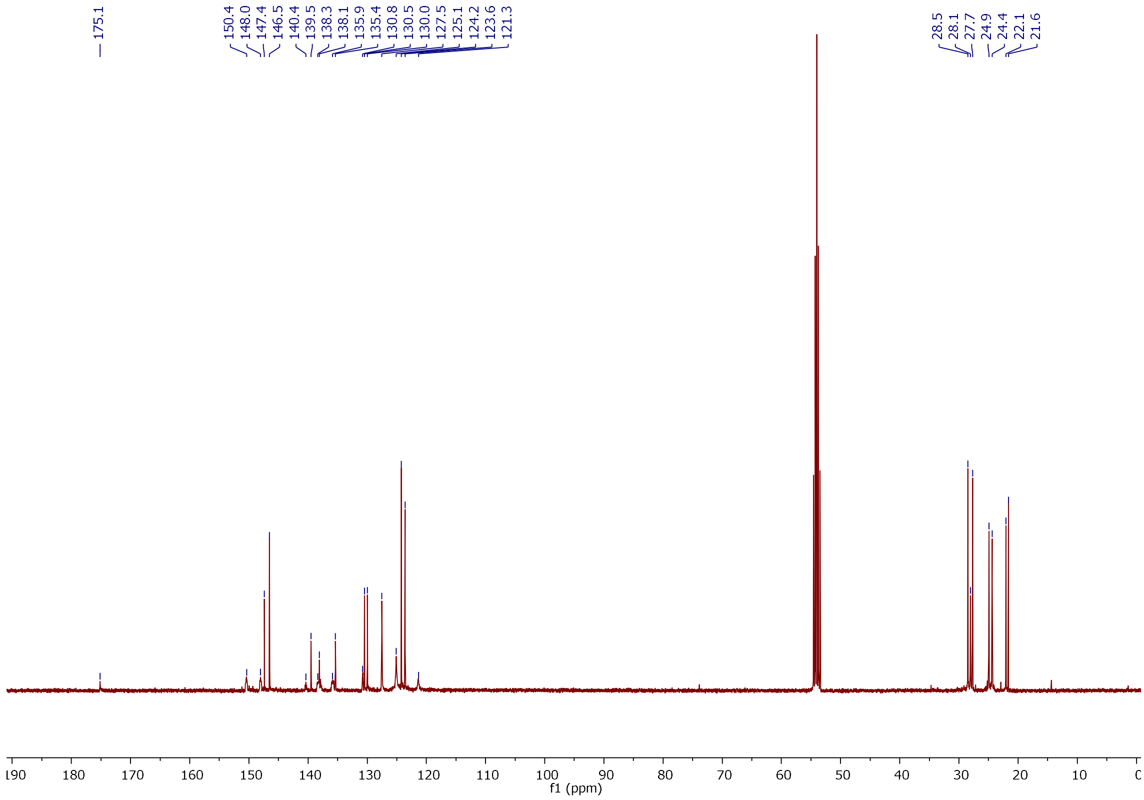

$^{11}B\{^1H\}$ NMR (128 MHz, dichloromethane-d_2) of [(WCA-IDipp)Cu(η^2-Tol)] (**5b**):

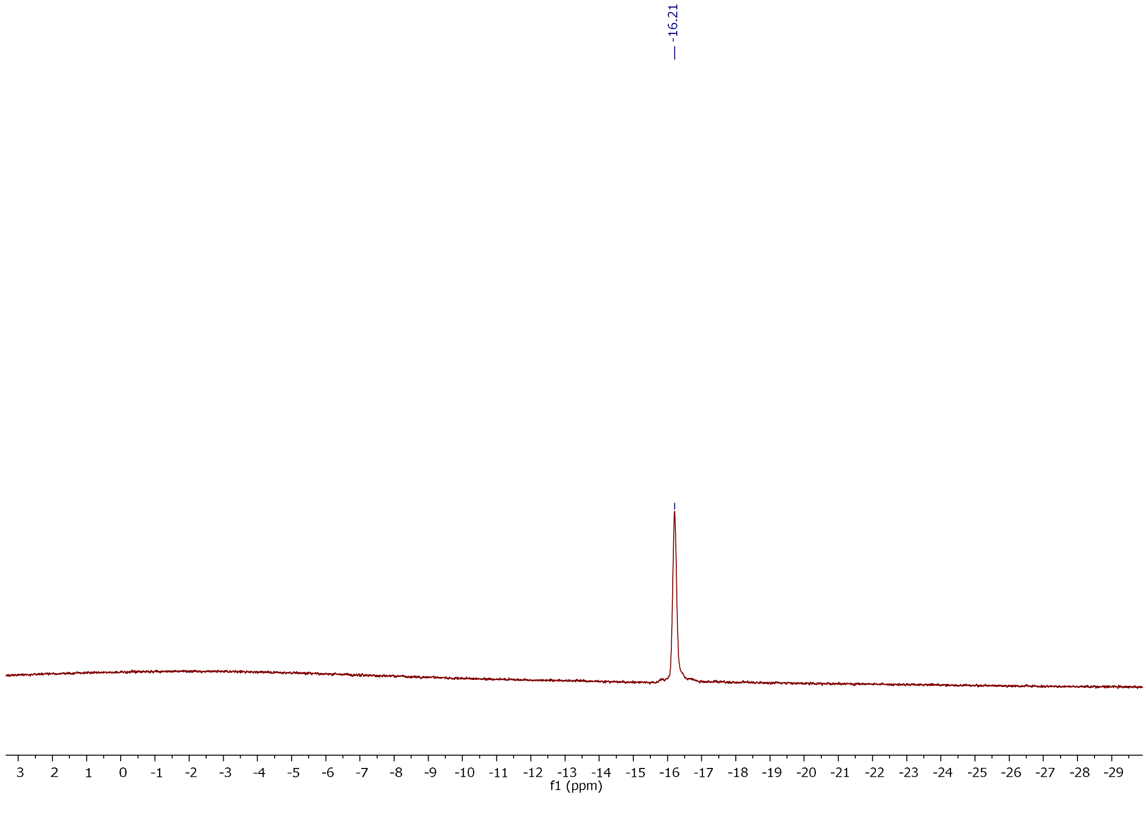

$^{19}F\{^{1}H\}$ NMR (376 MHz, dichloromethane-d_2) of [(WCA-IDipp)Cu(η^2-Tol)] (**5b**):

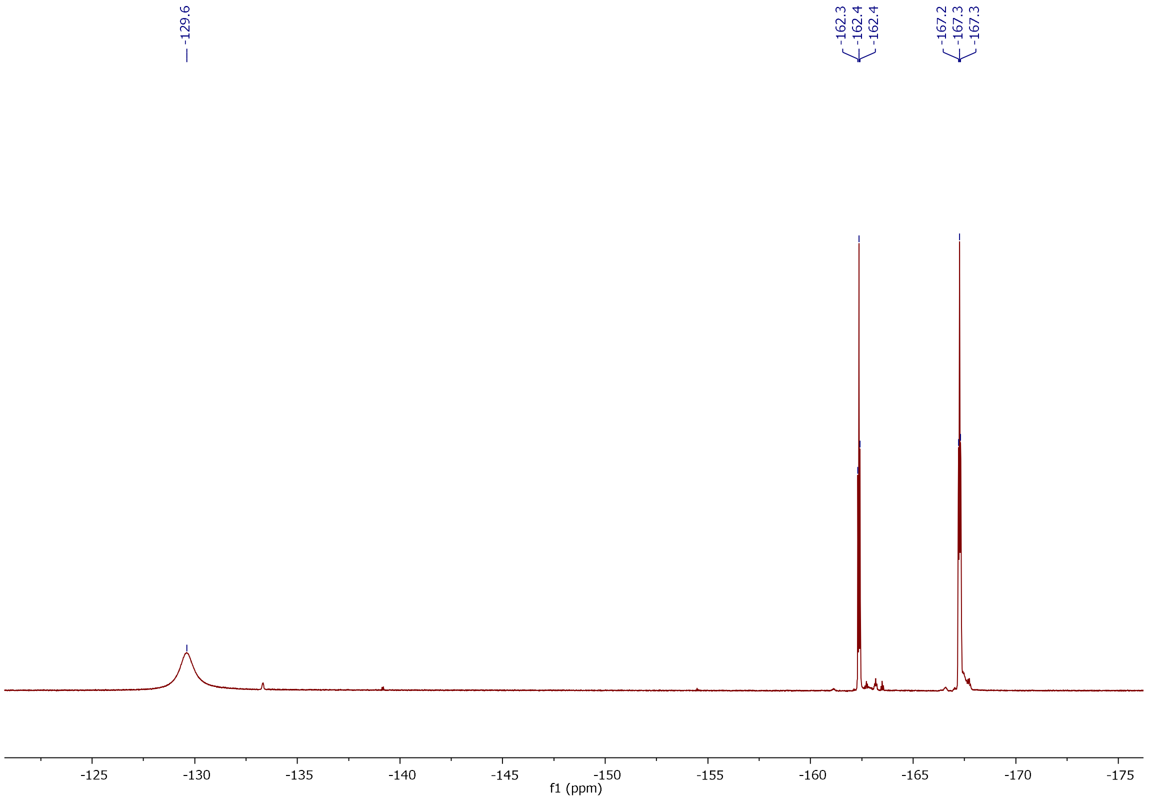

3.7 [(WCA-IDipp)Ag(μ-Cl$_2$)Ru(PPh$_3$)(η^6-*p*-cymene)] (6a)

^{1}H NMR (400 MHz, dichloromethane-d_2) of [(WCA-IDipp)Ag(μ-Cl$_2$)Ru(PPh$_3$)(η^6-*p*-cymene)] (**6a**):

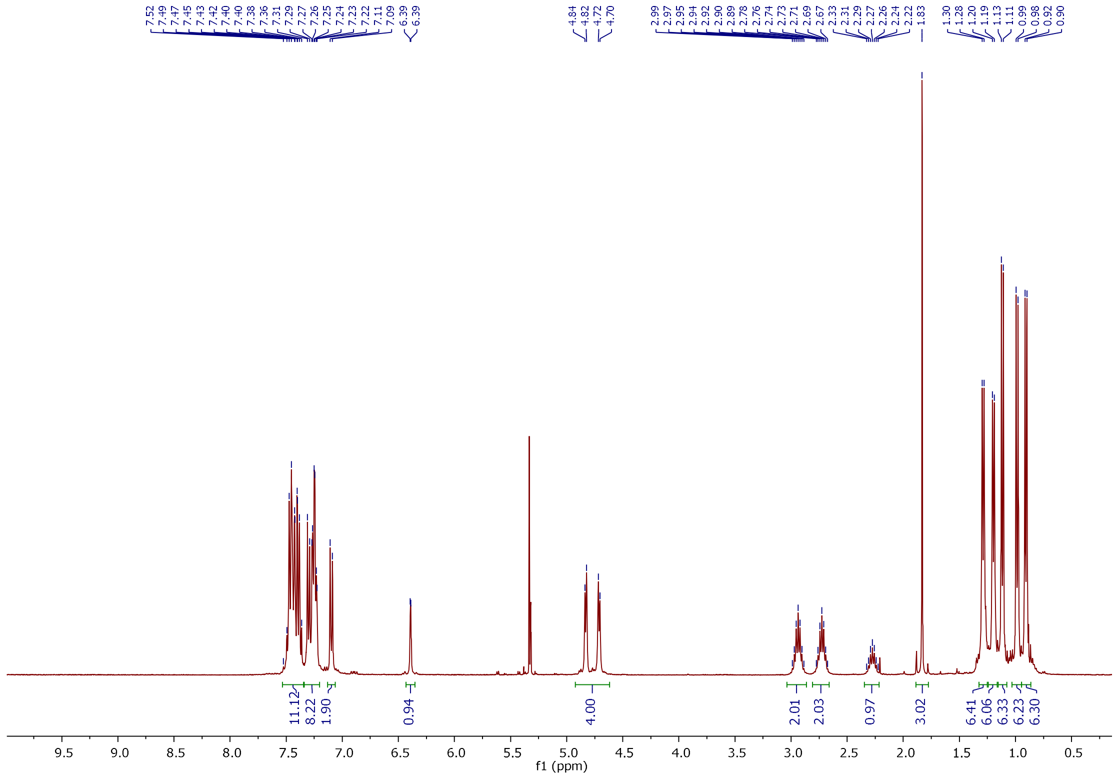

$^{13}C\{^1H\}$ NMR (101 MHz, dichloromethane-*d*$_2$) of [(WCA-IDipp)Ag(μ-Cl$_2$)Ru(PPh$_3$)(η^6-*p*-cymene)] (**6a**):

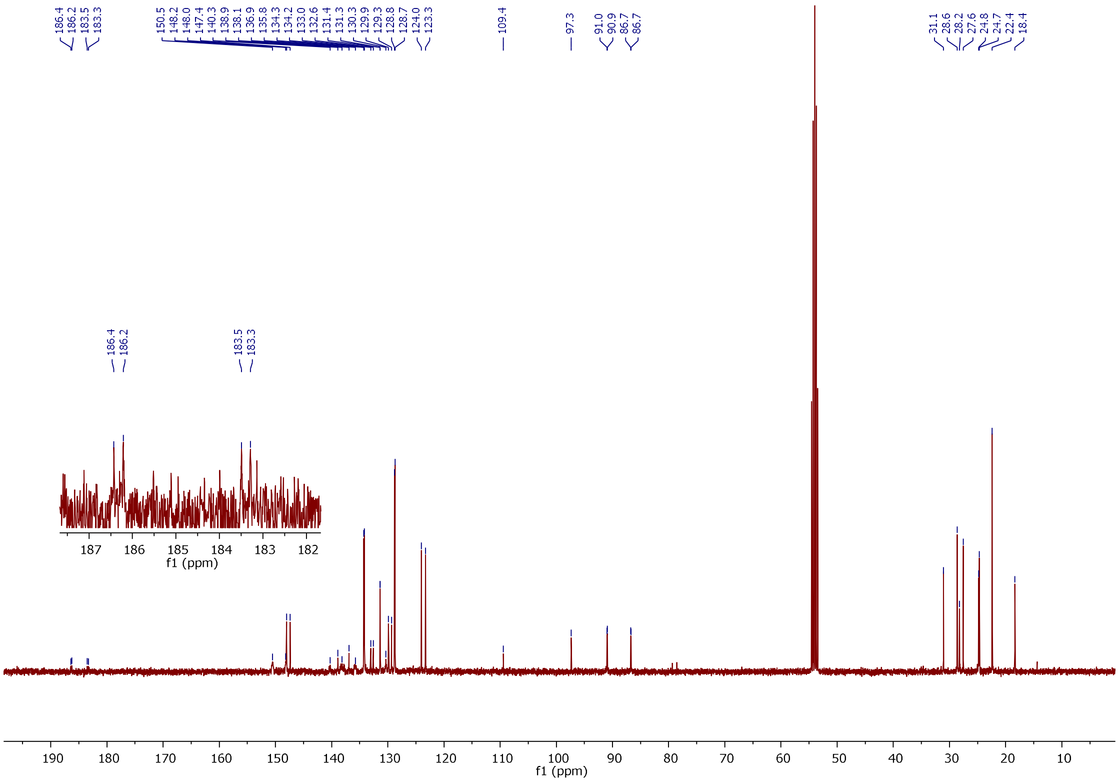

$^{11}B\{^1H\}$ NMR (128 MHz, dichloromethane-*d*$_2$) of [(WCA-IDipp)Ag(μCl$_2$)Ru(PPh$_3$)(η^6-*p*-cymene)] (**6a**):

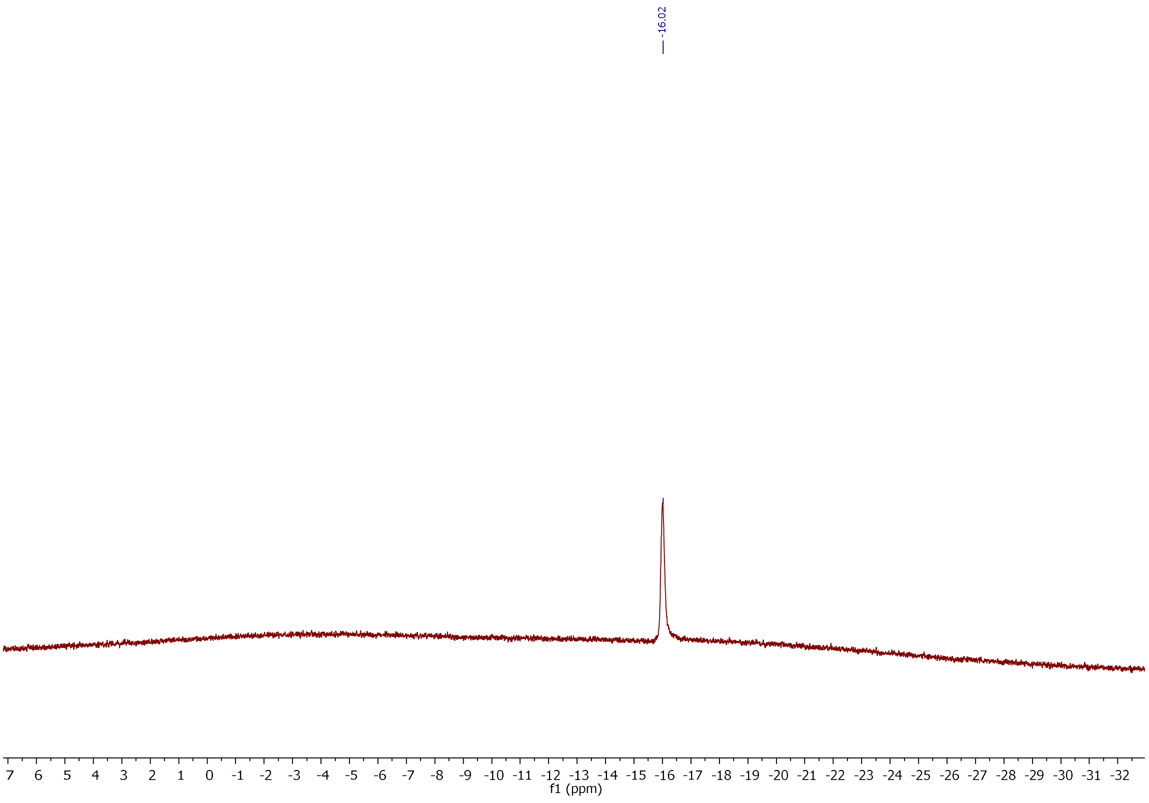

$^{19}F\{^{1}H\}$ NMR (376 MHz, dichloromethane-d_2) of [(WCA-IDipp)Ag(μCl_2)Ru(PPh_3)(η^6-*p*-cymene)] (**6a**):

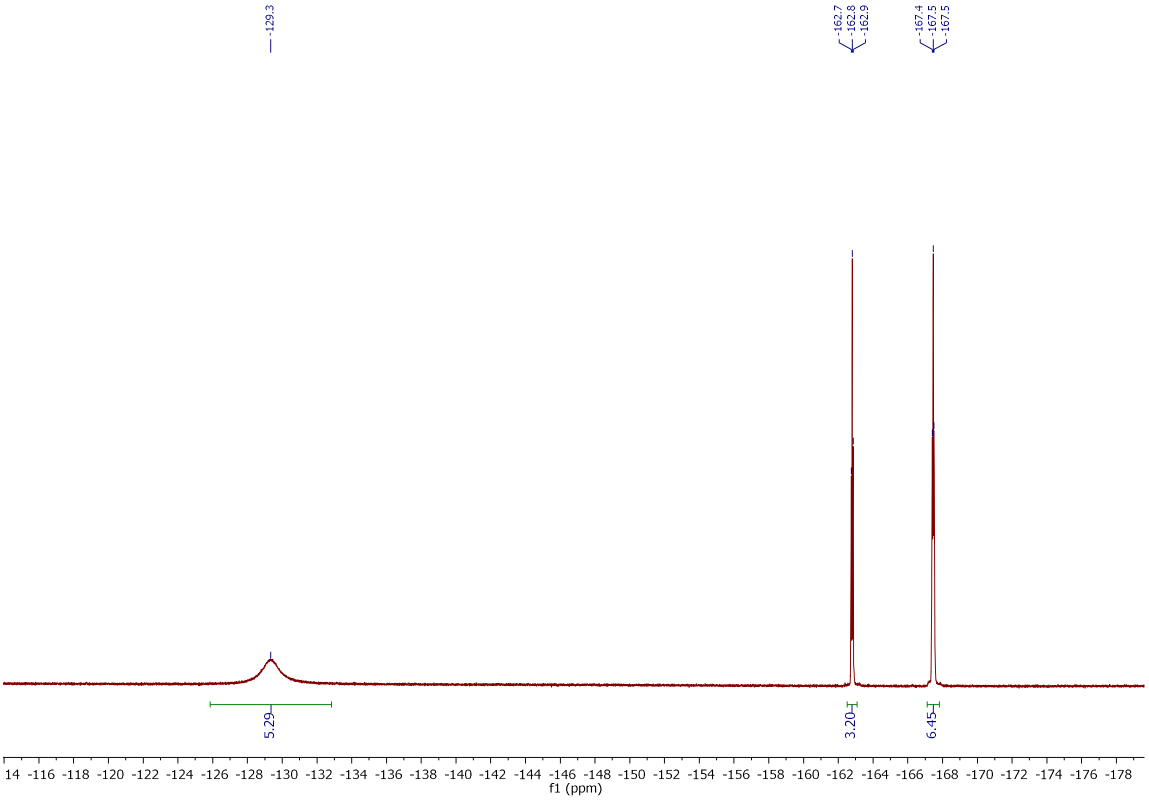

$^{31}P\{^{1}H\}$ NMR (162 MHz, dichloromethane-d_2) of [(WCA-IDipp)Ag(μCl_2)Ru(PPh_3)(η^6-*p*-cymene)] (**6a**):

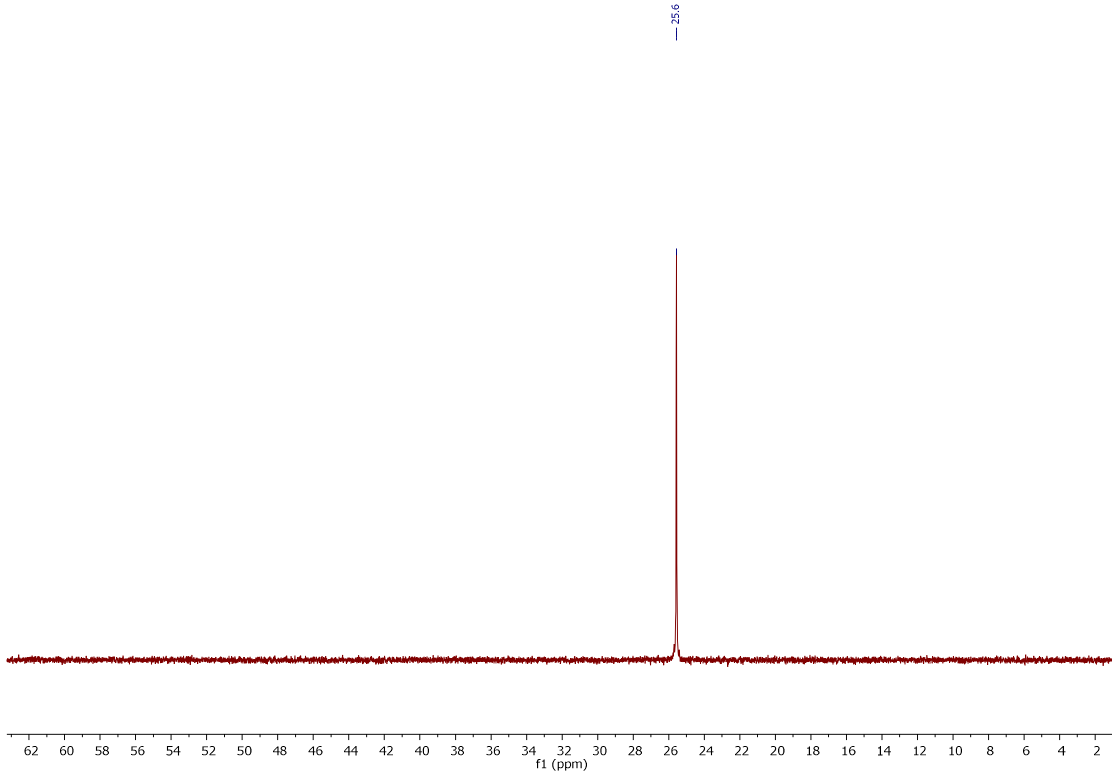

3.8 [(WCA-IDipp)Cu(μ-Cl_2)Ru(PPh_3)(η^6-*p*-cymene)] (6b)

^{1}H NMR (400 MHz, dichloromethane-*d*$_2$) of [(WCA-IDipp)Cu(μ-Cl_2)Ru(PPh_3)(η^6-*p*-cymene)] (**6b**):

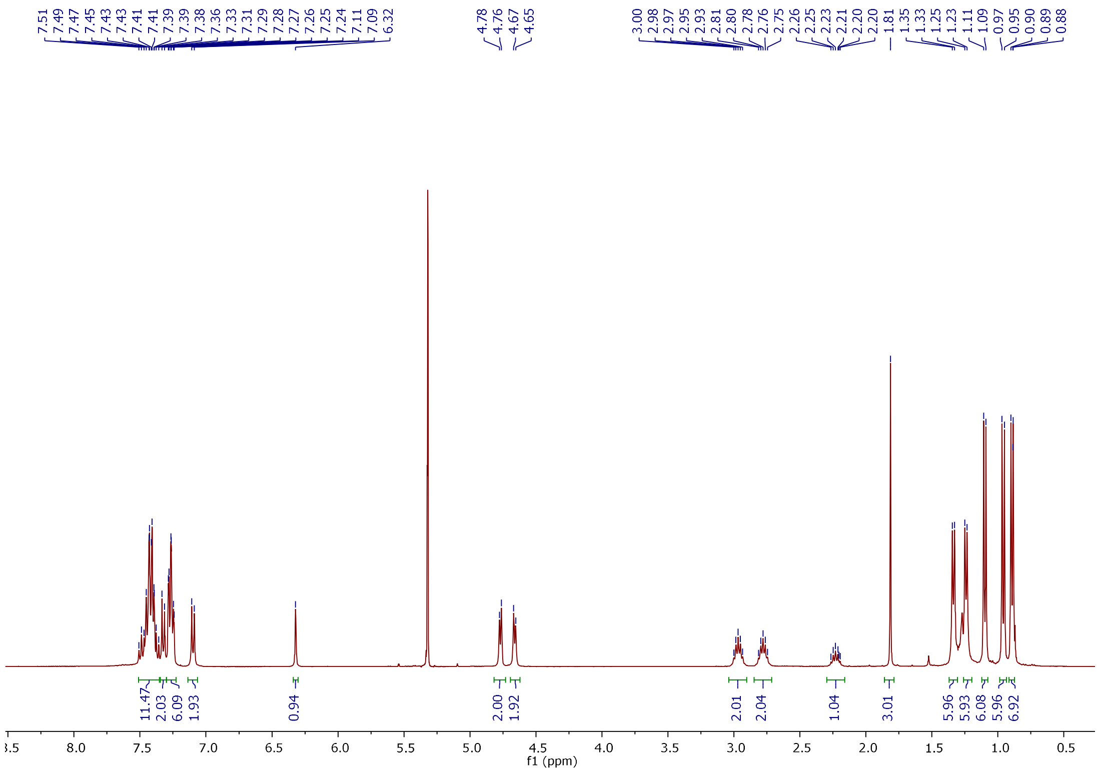

^{13}C{^{1}H} NMR (101 MHz, dichloromethane-*d*$_2$) of [(WCA-IDipp)Cu(μ-Cl_2)Ru(PPh_3)(η^6-*p*-cymene)] (**6b**):

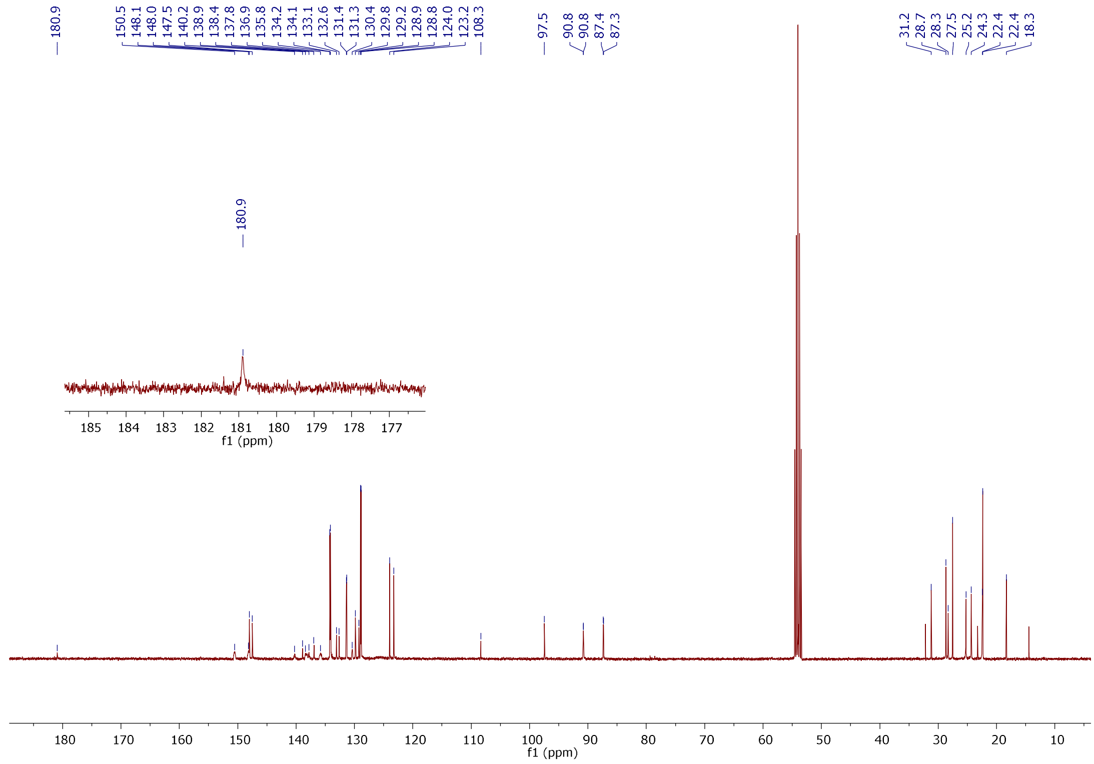

^{11}B{^{1}H} NMR (128 MHz, dichloromethane-d_2) of [(WCA-IDipp)Cu(μ-Cl$_2$)Ru(PPh$_3$)(η^6-*p*-cymene)] (**6b**):

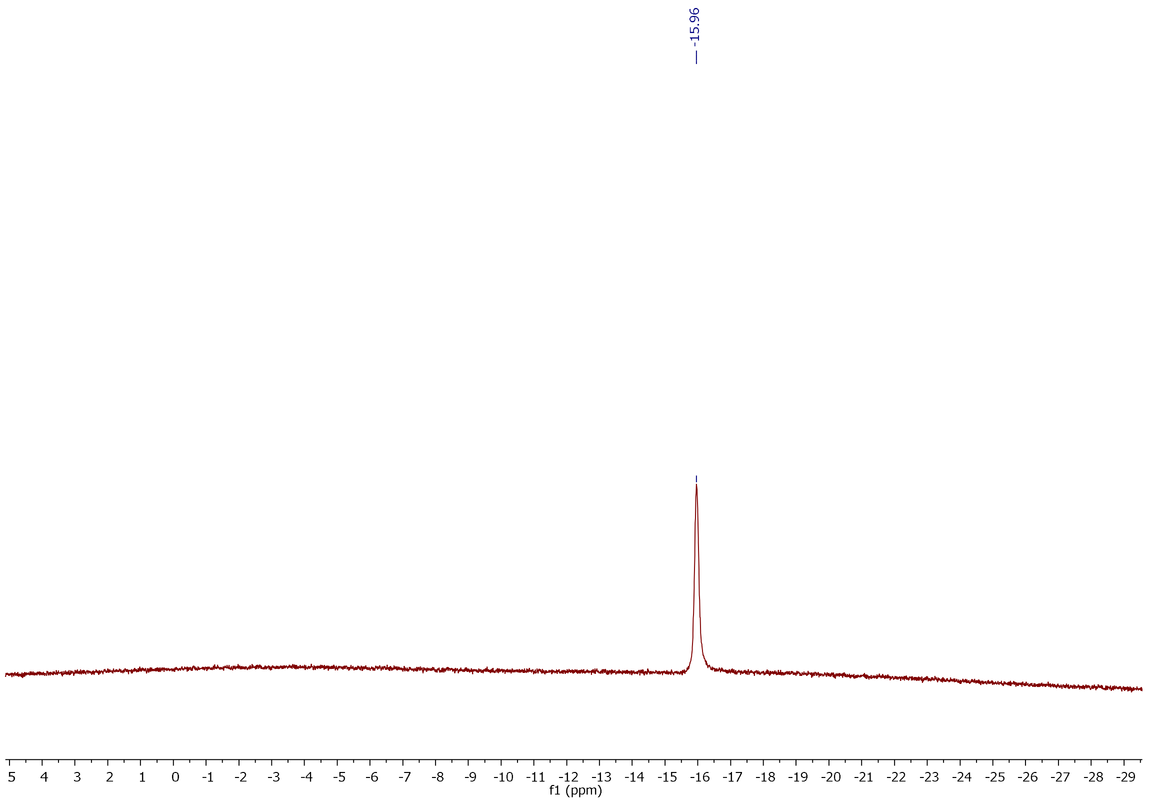

^{19}F{^{1}H} NMR (376 MHz, dichloromethane-d_2) of [(WCA-IDipp)Cu(μ-Cl$_2$)Ru(PPh$_3$)(η^6-*p*-cymene)] (**6b**):

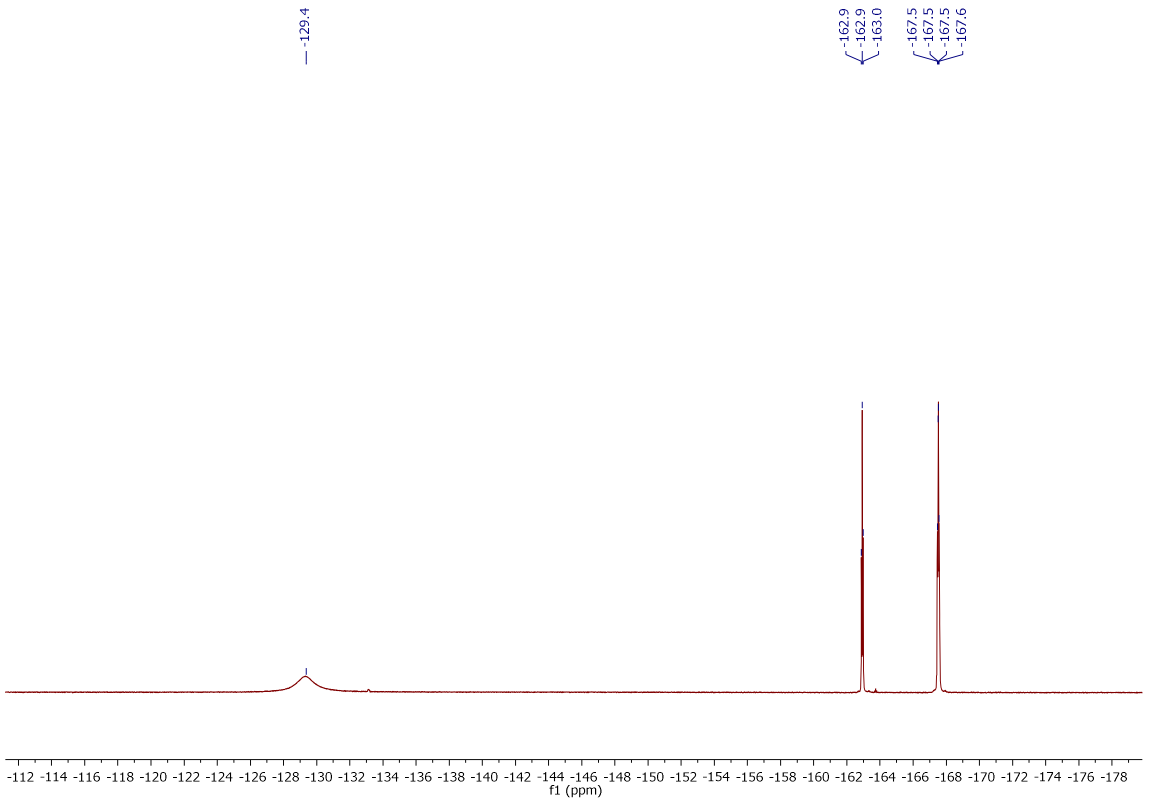

$^{31}P\{^1H\}$ NMR (162 MHz, dichloromethane-d_2) of [(WCA-IDipp)Cu(μ-Cl_2)Ru(PPh_3)(η^6-*p*-cymene)] (**6b**):

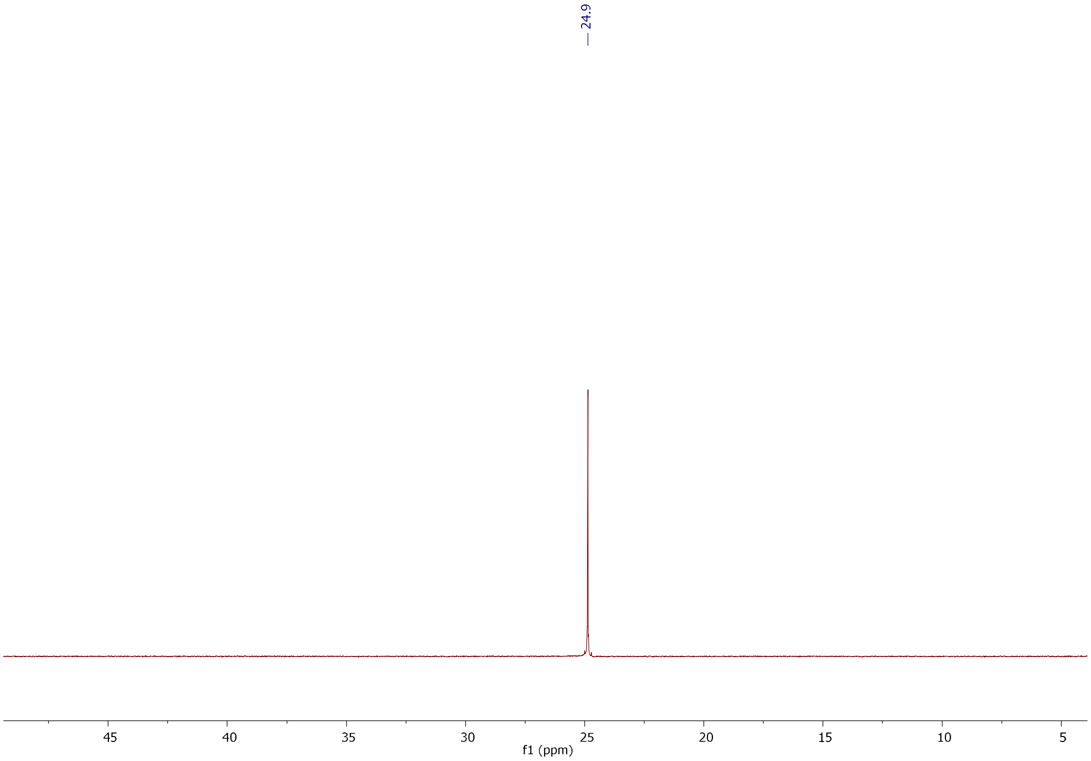

3.9 [(WCA-IDipp)Ag(μ-I_2)Ru(PPh_3)(η^6-*p*-cymene)] (7)

^1H NMR (400 MHz, dichloromethane-d_2) of [(WCA-IDipp)Ag(μ-I_2)Ru(PPh_3)(η^6-*p*-cymene)] (**7**):

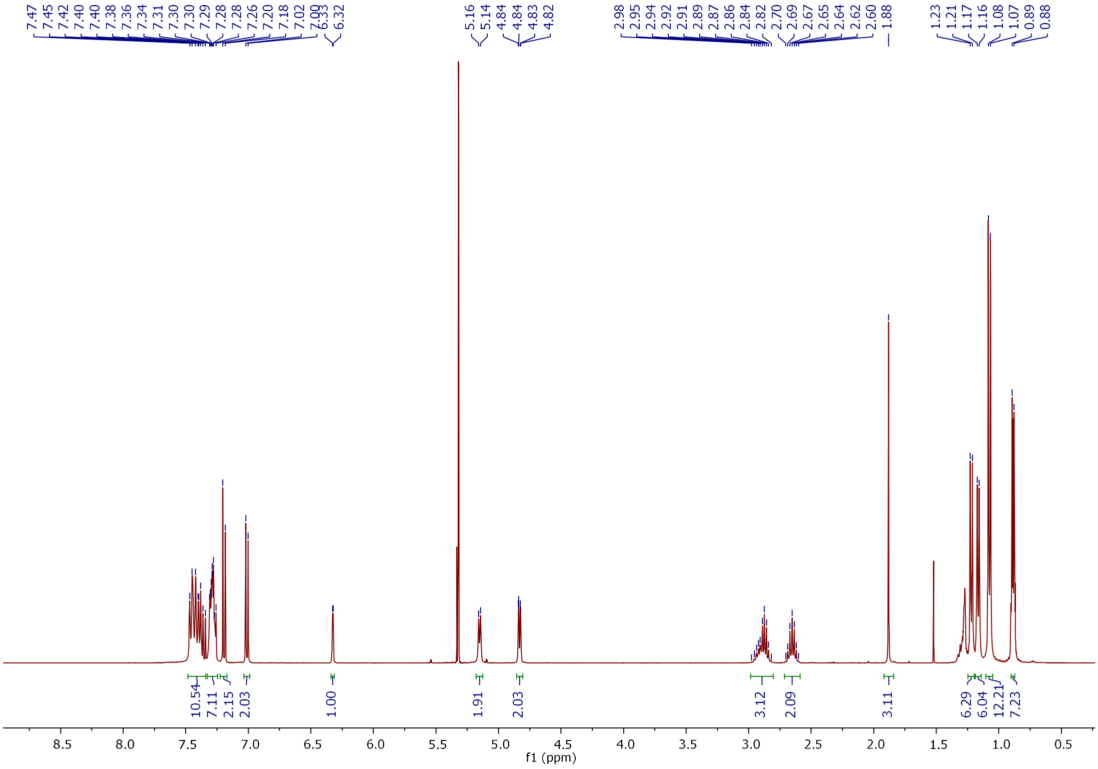

$^{13}C\{^{1}H\}$ NMR (101 MHz, dichloromethane-d_2) of [(WCA-IDipp)Ag(μ-I_2)Ru(PPh_3)(η^6-*p*-cymene)] (**7**):

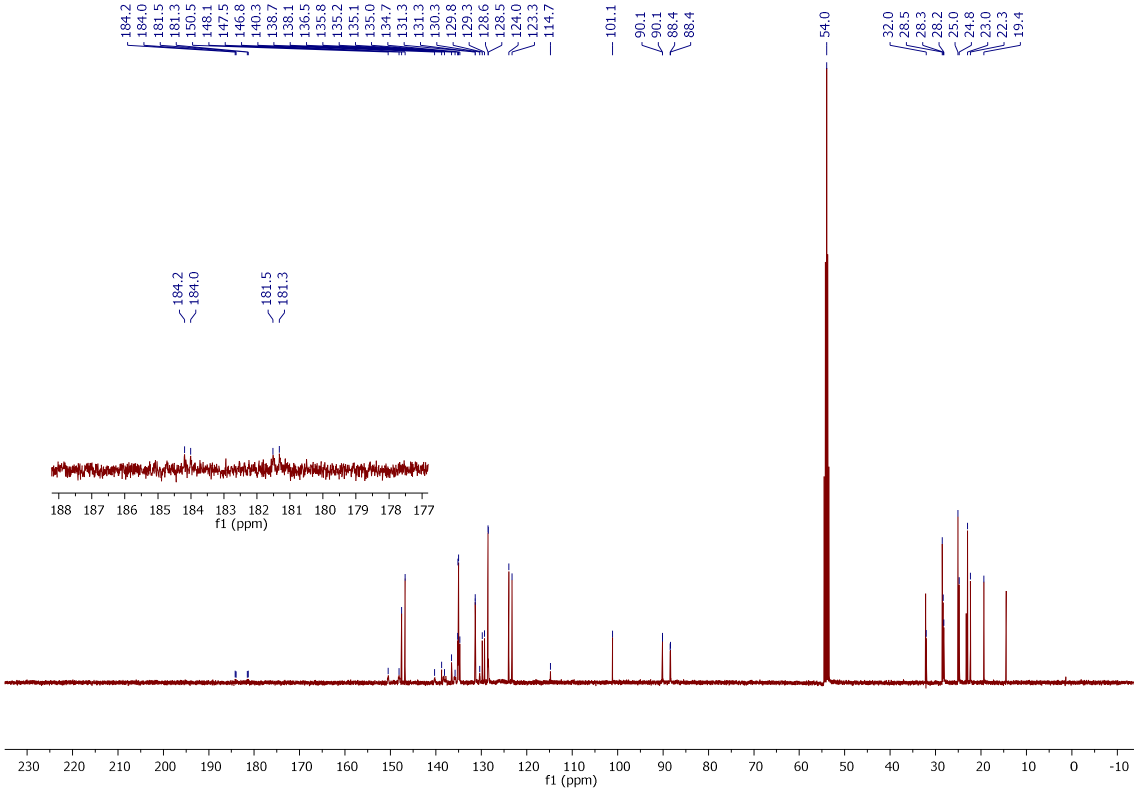

$^{11}B\{^{1}H\}$ NMR (128 MHz, dichloromethane-d_2) of [(WCA-IDipp)Ag(μ-I_2)Ru(PPh_3)(η^6-*p*-cymene)] (**7**):

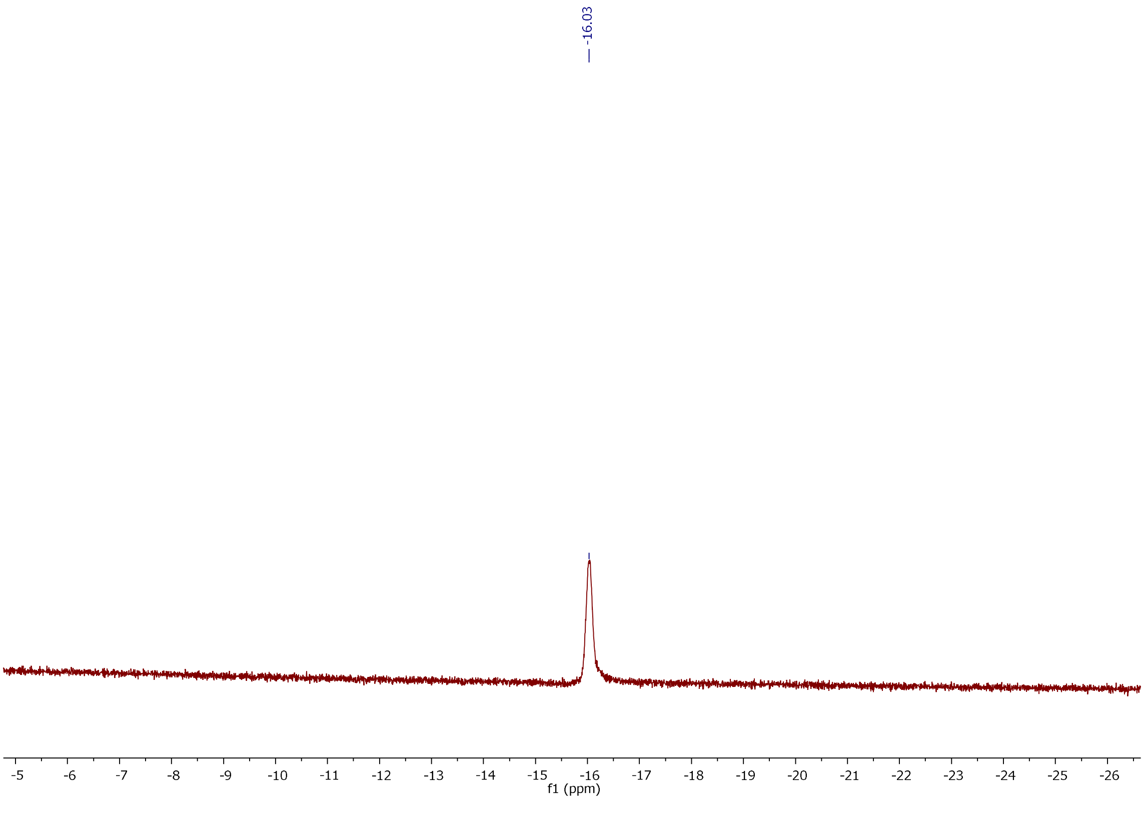

$^{19}F\{^1H\}$ NMR (376 MHz, dichloromethane-d_2) of [(WCA-IDipp)Ag(μ-I_2)Ru(PPh_3)(η^6-*p*-cymene)] (**7**):

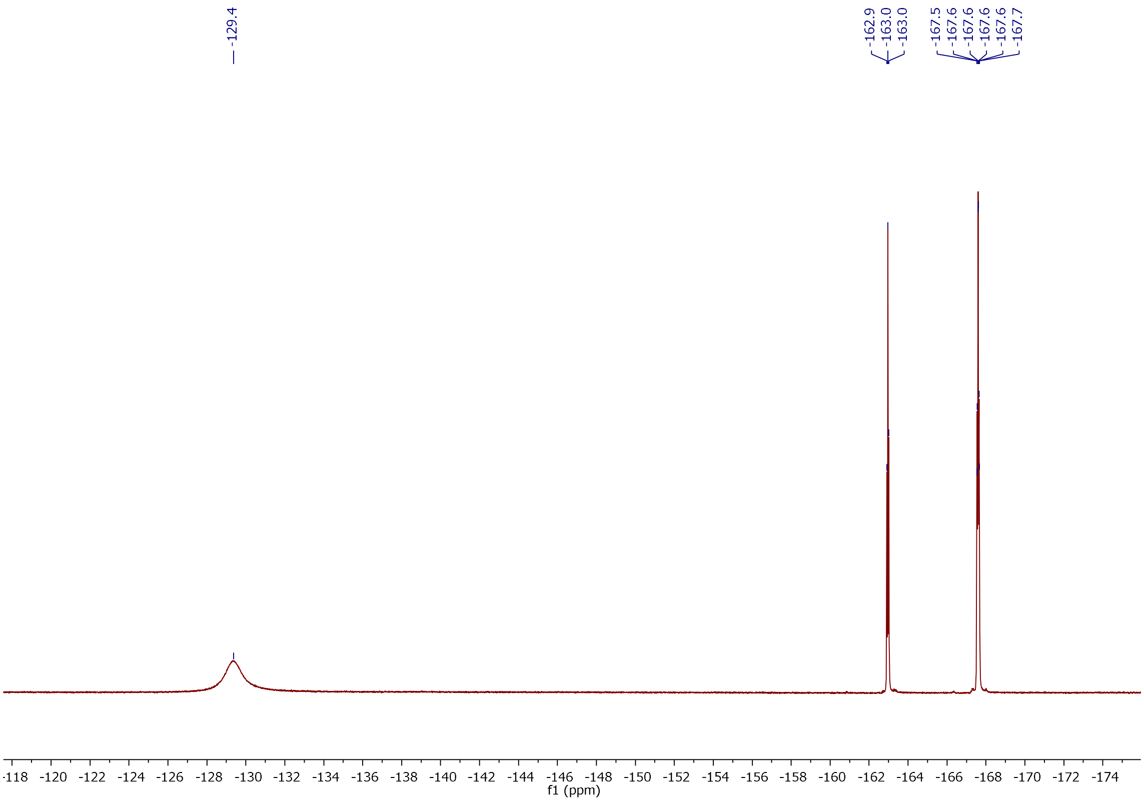

$^{31}P\{^1H\}$ NMR (162 MHz, dichloromethane-d_2) of [(WCA-IDipp)Ag(μ-I_2)Ru(PPh_3)(η^6-*p*-cymene)] (**7**):

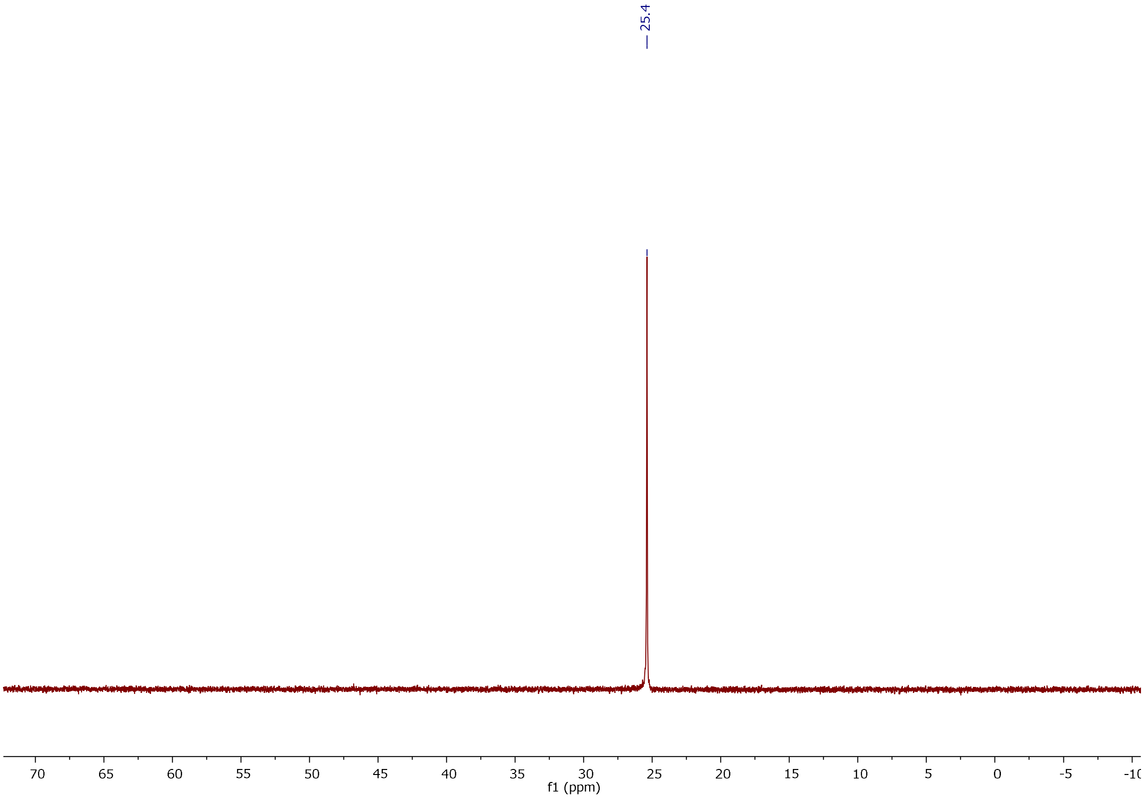

3.10 [(WCA-IDipp)Cu(Cl)][(Ru(η^6-*p*-cymene))$_2$(μ-Cl$_3$)]

^{1}H NMR (400 MHz, dichloromethane-*d*$_2$) of [(WCA-IDipp)Cu(Cl)][(Ru(η^6-*p*-cymene))$_2$(μ-Cl$_3$)]:

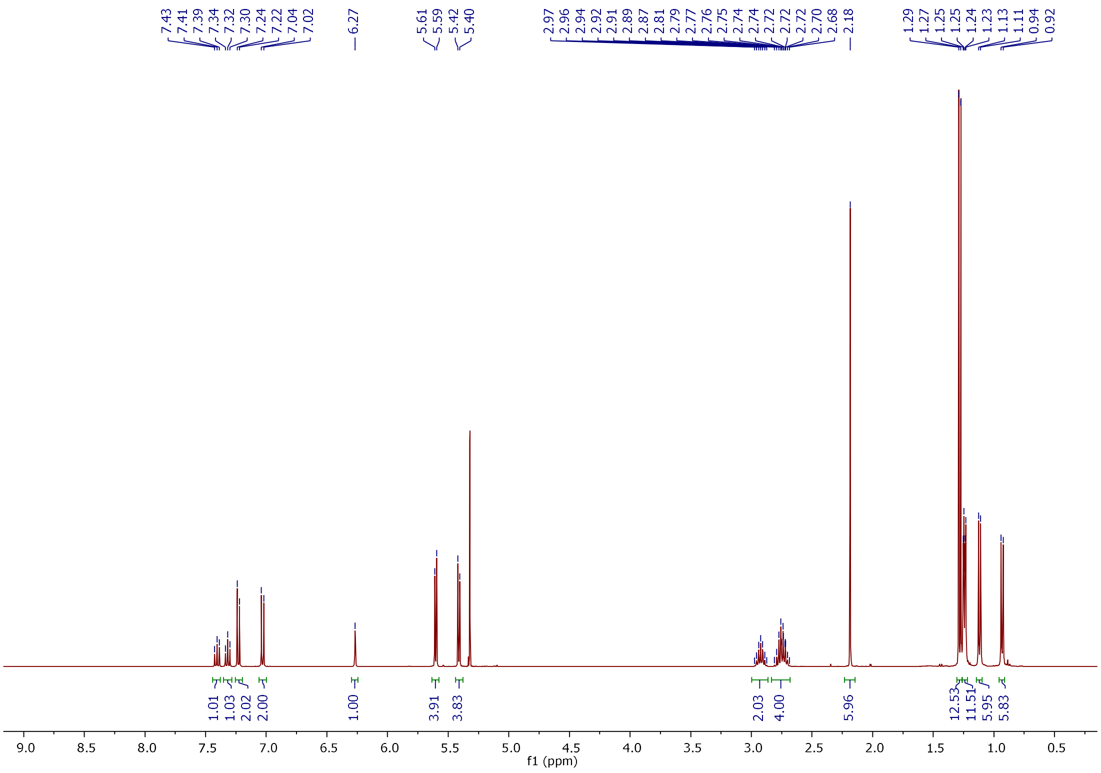

^{13}C{^{1}H} NMR (101 MHz, dichloromethane-*d*$_2$) of [(WCA-IDipp)Cu(Cl)][(Ru(η^6-*p*-cymene))$_2$(μ-Cl$_3$)]:

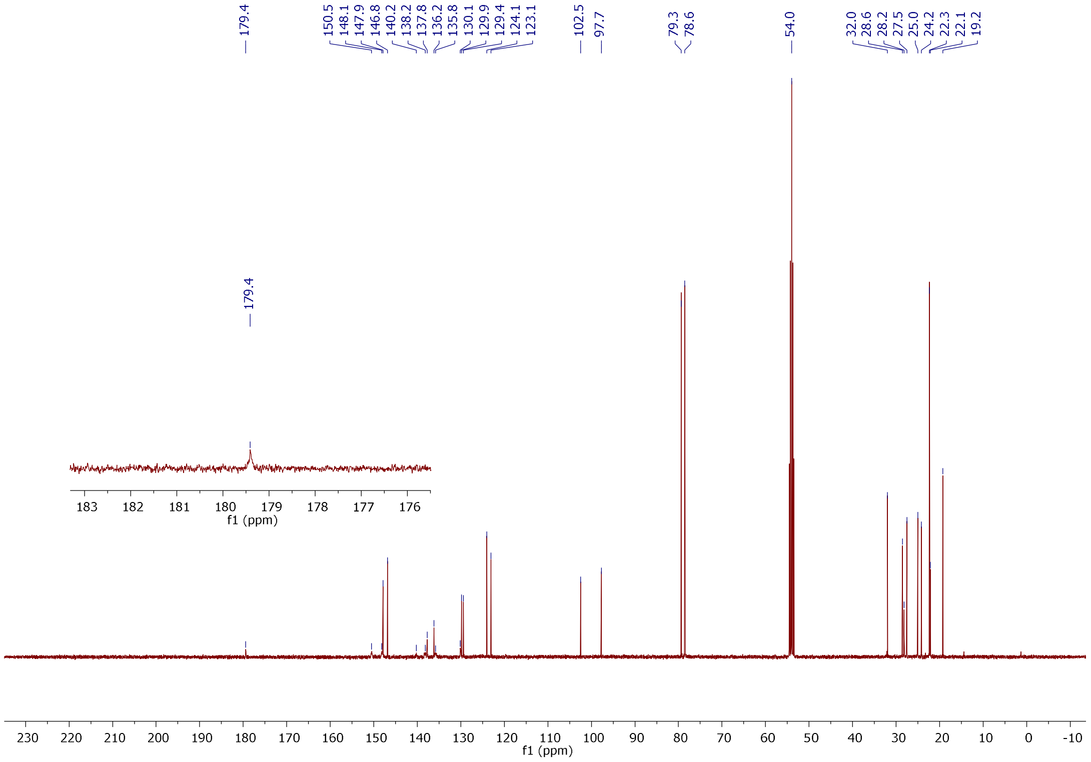

$^{11}B\{^{1}H\}$ NMR (128 MHz, dichloromethane-*d*$_2$) of [(WCA-IDipp)Cu(Cl)][(Ru(η^6-*p*-cymene))$_2$(μ-Cl$_3$)]:

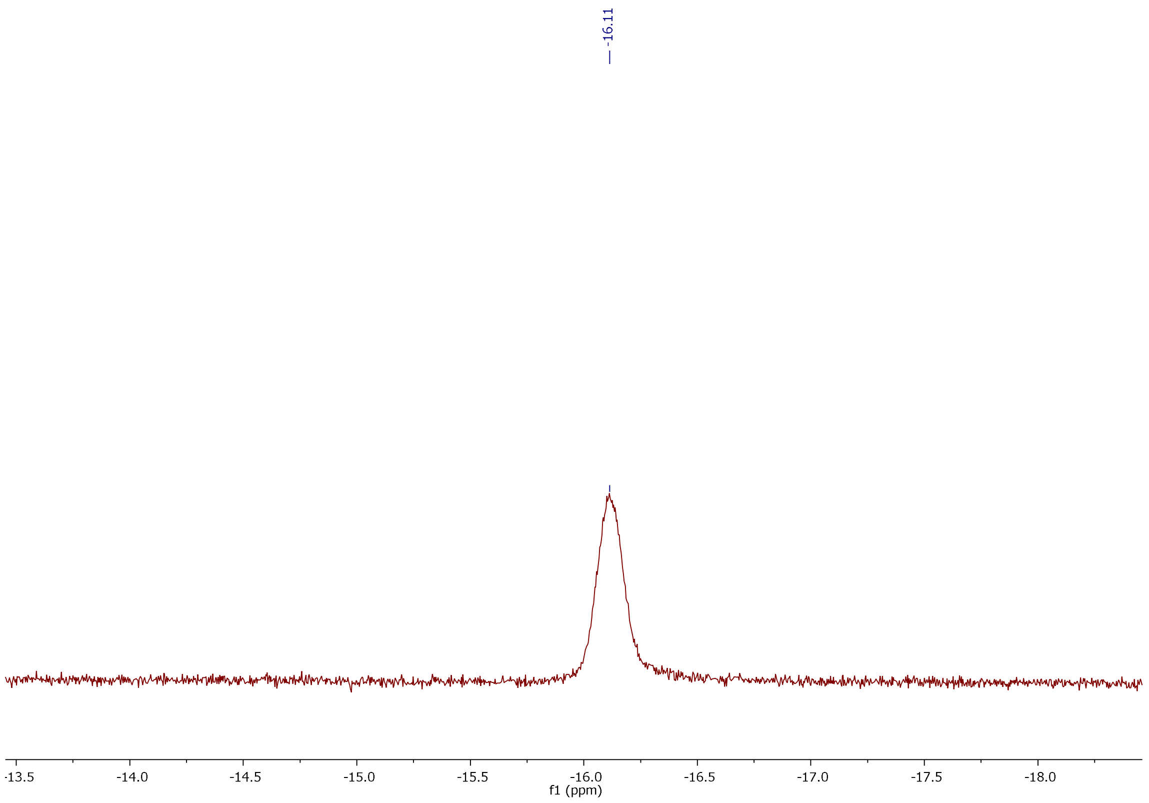

$^{19}F\{^{1}H\}$ NMR (376 MHz, dichloromethane-*d*$_2$) of [(WCA-IDipp)Cu(Cl)][(Ru(η^6-*p*-cymene))$_2$(μ-Cl$_3$)]:

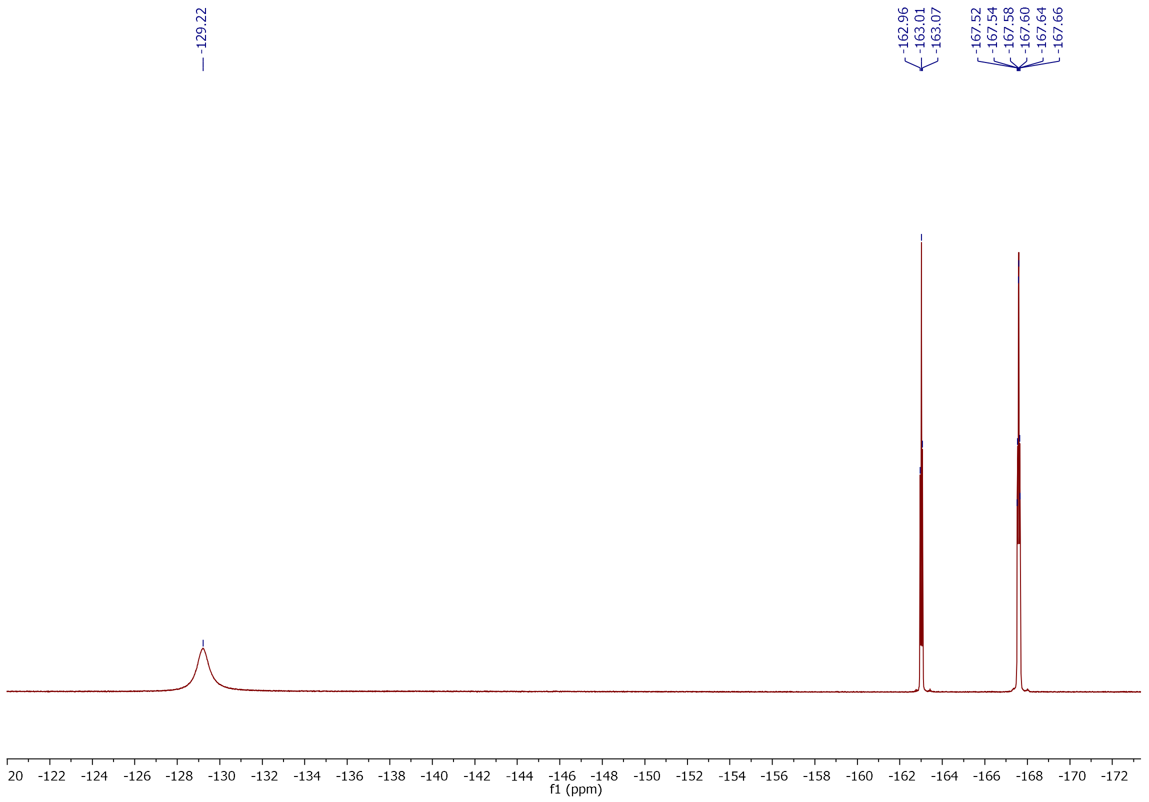

4 X-Ray Crystal Structure Determinations

For a summary of crystal data, see Tables S4.1 - 4.11. Crystals were mounted on either glass fibers, human hair, MiTiGen or Hampton mounts in perfluorinated inert oil. Intensity measurements were performed at 100 K using a Rigaku XtaLAB Synergy S Single Source and an Oxford Diffraction Nova A diffractometer with mirror-focussed Cu*K*α radiation or a Rigaku XtaLAB Synergy S Single Source with mirror-focused Mo*K*α and an Oxford Diffraction Xcalibur Eos diffractometer with monochromated Mo*K*α radiation. Additional measurements were performed using an Agilent Technologies SuperNova Dual Source device with both Cu*K*α or Mo*K*α radiation.

Data reduction was performed with the CrysAlisPRO software.[A] Absorption corrections were based on multi-scans, analytical methods or face-indexation using a gaussian grid. The structures solved using either direct methods in SHELXS[B] or intrinsic phasing in SHELXT[C] and were refined anisotropically on F^2 using the program SHELXL[D] in OLEX2.[E]

The hydrogen atoms were, unless otherwise noted, included either as constituents of idealized rigid methyl groups allowed to rotate but not tip, or using a riding model starting from calculated positions. The implementation of BYPASS[F] in OLEX2 had to be used for compound **7**.

Further details are given in the corresponding Tables under “Measurement and Refinement Details”.

CCDC 2096235-2096244 contain the supplementary crystallographic data for this paper. These data are provided free of charge by the Cambridge Crystallographic Data Centre.

References:

[A] Rigaku Oxford Diffraction, *CrysAlisPRO Software System*, Rigaku Corporation, Oxford, UK.

[B] G. M. Sheldrick, *Acta Cryst.* **2007**, *A64*, 112-122.

[C] G. M. Sheldrick, *Acta Cryst.* **2015**, *A71*, 3–8.

[D] G. M. Sheldrick, *Acta Cryst.* **2015**, *C71*, 3–8.

[E] O. V. Dolomanov, L. J. Bourhis, R. J. Gildea, J. A. K. Howard, H. Puschmann, *J. Appl. Cryst.* **2009**, *42*, 339–341.

[F] P. van der Sluis, A. L. Spek, *Acta Cryst.* **1990**, *A46*, 194-201.

4.1 [Li(THF)$_4$][(WCA-IDipp)$_2$Ag] (2·THF)

Compound	2·THF
Identification code	2096235
Empirical formula	$C_{110}H_{110}AgB_2F_{30}LiN_4O_5$
Formula weight	2274.44
Temperature	100(2) K
Wavelength	0.71073 Å
Instrument (scan mode)	Oxford Diffraction Xcalibur, Eos (ω scan)
Crystal system	Triclinic
Space group	*P*-1
Unit cell dimensions	a = 14.9781(5) Å α = 94.727(3)°<br>b = 16.0776(6) Å β = 93.650(3)°<br>c = 21.6387(7) Å γ = 91.048(3)°
Volume	5181.1(3) Å^3
Z	2
Density (calculated)	1.458 Mg/m^3
Absorption coefficient	0.305 mm^{-1}
F(000)	2336
Crystal habitus	prism (colourless)
Crystal size	0.3 x 0.2 x 0.15 mm^3
Theta range for data collection	2.190 to 29.308°
Index ranges	-20<=h<=20, -21<=k<=21, -29<=l<=29
Reflections collected	162994
Independent reflections	26040 [R(int) = 0.0807]
Completeness to theta = 25.242°	99.9 %
Absorption correction	Semi-empirical from equivalents
Max. and min. transmission	1.00000 and 0.98467
Refinement method	Full-matrix least-squares on F^2
Data / restraints / parameters	26040 / 30 / 1423
Goodness-of-fit on F^2	1.028
Final R indices [I>2sigma(I)]	R1 = 0.0556, wR2 = 0.1050
R indices (all data)	R1 = 0.1003, wR2 = 0.1229
Largest diff. peak and hole	0.839 and -0.634 e.Å^{-3}
Solution:	SHELXS-97 (Sheldrick, 1990)
Refinement:	SHELXL-2018/3 (G. M. Sheldrick, Acta Cryst. (2008), A64, 112-122)
Measurement and Refinement Details:	Two THF molecules of the Li(THF)4 fragment are partially disordered over two positions and were treated accordingly. One free THF molecule was restrained employing an ISOR command. Disorder was

	checked but gave no satisfactory refinement.

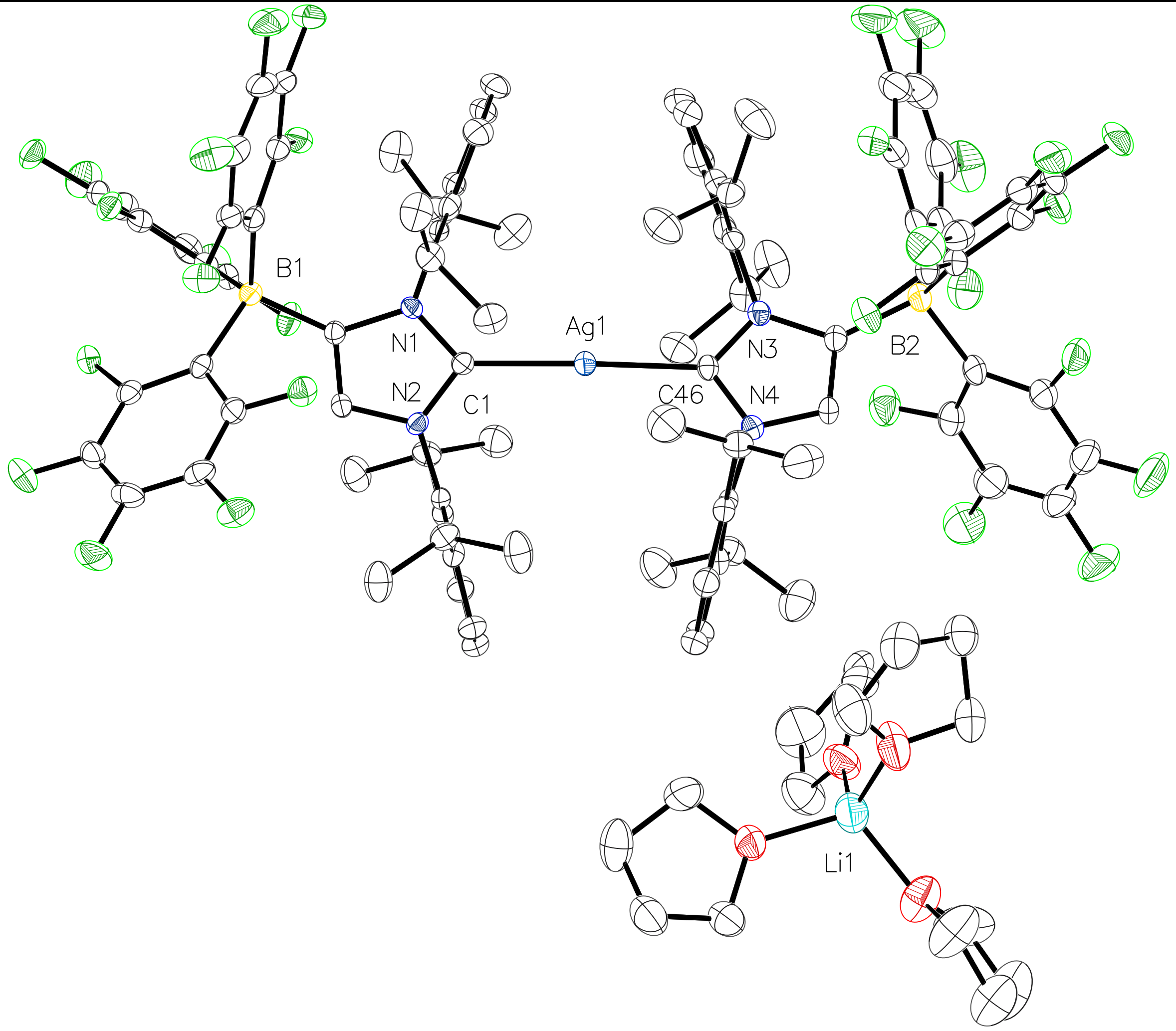

Figure S1. Molecular structure of **2**·THF with thermal displacement parameters drawn at 50% probability. All hydrogen atoms and one molecule THF are omitted for clarity. Selected bond lengths [Å] and angles [°]: Ag1−C1 2.141(3), Ag1−C46 2.151(3), B1−C2 1.660(4), B2−C47 1.656(4), N1−C1−N2 103.4(2), N3−C46−N4 103.5(2), C1−Ag1−C46 178.08(10), Ag1−C1−N1 130.56(18), Ag1−C1−N2 125.90(17), Ag1–C46–N3 129.27(19), Ag1−C46−N4 127.15(18).

4.2 [Li(THF)$_n$][(WCA-IDipp)CuCl] (3)

Compound	3
Identification code	2096236
Empirical formula	$C_{118}H_{126}B_2Cl_2Cu_2F_{30}Li_2N_4O_7$
Formula weight	2515.70
Temperature	100(2) K
Wavelength	1.54184 Å
Instrument (scan mode)	SuperNova, Dual, Cu at home/near, Atlas (ω scan)
Crystal system	Monoclinic
Space group	$P2_1/c$
Unit cell dimensions	a = 35.2348(6) Å α = 90°<br>b = 10.7019(2) Å β = 109.3856(18)°<br>c = 32.8617(6) Å γ = 90°
Volume	11689.0(4) Å^3
Z	4
Density (calculated)	1.430 Mg/m^3
Absorption coefficient	1.778 mm^{-1}
F(000)	5184
Crystal habitus	plate (orange)
Crystal size	0.237 x 0.066 x 0.064 mm^3
Theta range for data collection	2.659 to 67.075°
Index ranges	-42<=h<=42, -12<=k<=11, -39<=l<=37
Reflections collected	122215
Independent reflections	20859 [R(int) = 0.0713]
Completeness to theta = 67.075°	100.0 %
Absorption correction	Analytical
Max. and min. transmission	0.909 and 0.780
Refinement method	Full-matrix least-squares on F^2
Data / restraints / parameters	20859 / 38 / 1556
Goodness-of-fit on F^2	1.059
Final R indices [I>2sigma(I)]	R1 = 0.0546, wR2 = 0.1092
R indices (all data)	R1 = 0.0747, wR2 = 0.1177
Largest diff. peak and hole	0.908 and -0.575 e.Å^{-3}
Refinement:	SHELXL-2018/3 (G. M. Sheldrick, Acta Cryst., 2015, C71, 3-8)
Measurement and Refinement Details:	Several of the coordinating THF molecules are partially disordered over two positions and are treated accordingly. To yield a stable refinement several restraints were applied.

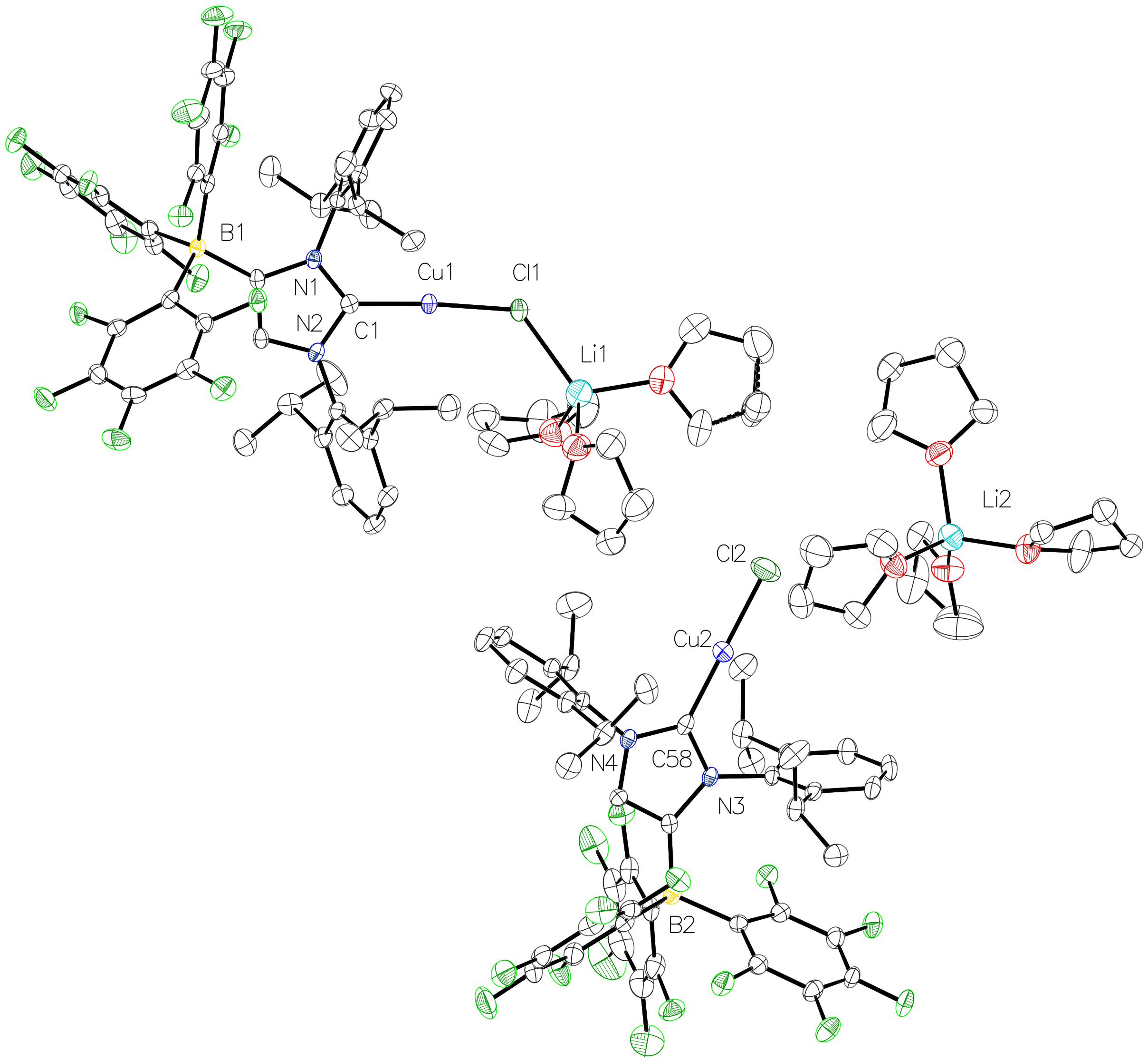

Figure S2. Molecular structure of **3** with thermal displacement parameters drawn at 50% probability level. All hydrogen atoms are omitted for clarity. Selected bond lengths [Å] and angles [°]: Cu1−Cl1 2.1353(6), Cu2−Cl1 2.1282(6), Cu1−C1 1.877(3), Cu2−C58 1.879(3), B1−C2 1.651(4), B2−C59 1.645(4), Cu1−Cl1−Li1 128.56(17), C1−Cu1−Cl1 175.59(9), C58−Cu2−Cl2 179.18(10), N1−C1−N2 104.5(2), N3−C58−N4 103.4(2).

4.3 [(WCA-IDipp)Ag(PPh$_3$)] (4a)

Compound	4a
**Identification code**	2096237
**Empirical formula**	$C_{63}H_{50}AgBF_{15}N_2P$
**Formula weight**	1269.70
**Temperature**	100(2) K
**Wavelength**	0.71073 Å
**Instrument (scan mode)**	XtaLAB Synergy, Single source at off-set/far, HyPix (ω scan)
**Crystal system**	Monoclinic
**Space group**	$P2_1/n$
**Unit cell dimensions**	a = 11.0474(2) Å α = 90°<br>b = 26.2425(4) Å β = 91.332(2)°<br>c = 19.2492(2) Å γ = 90°
**Volume**	5579.06(14) Å^3
**Z**	4
**Density (calculated)**	1.512 Mg/m^3
**Absorption coefficient**	0.484 mm^{-1}
**F(000)**	2576
**Crystal habitus**	block (colourless)
**Crystal size**	0.330 x 0.215 x 0.142 mm^3
**Theta range for data collection**	2.558 to 32.575°
**Index ranges**	-16<=h<=16, -38<=k<=36, -28<=l<=28
**Reflections collected**	287785
**Independent reflections**	18674 [R(int) = 0.0481]
**Completeness to theta = 25.242°**	99.9 %
**Absorption correction**	Gaussian
**Max. and min. transmission**	1.000 and 0.358
**Refinement method**	Full-matrix least-squares on F^2
**Data / restraints / parameters**	18674 / 0 / 756
**Goodness-of-fit on F^2**	1.093
**Final R indices [I>2sigma(I)]**	R1 = 0.0545, wR2 = 0.1541
**R indices (all data)**	R1 = 0.0605, wR2 = 0.1573
**Largest diff. peak and hole**	2.232 and -2.045 e.Å^{-3}
**Solution:**	SHELXT-2014/5 (G. M. Sheldrick, Acta Cryst., 2015, A71, 3-8)
**Refinement:**	SHELXL-2018/3 (G. M. Sheldrick, Acta Cryst. (2008), A64, 112-122)
**Measurement and Refinement Details:**	-

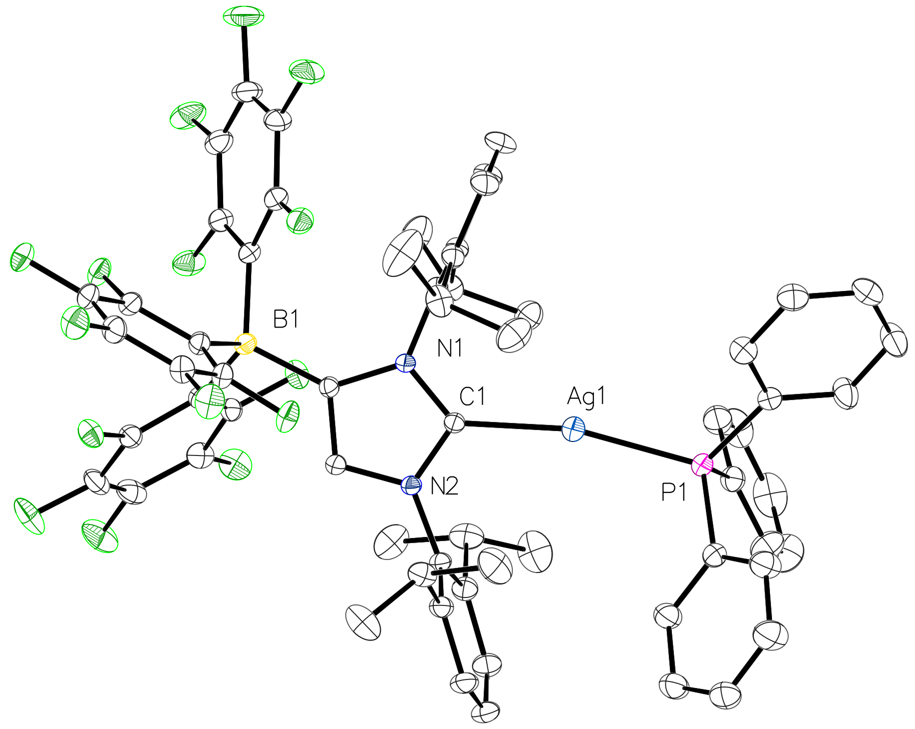

Figure S3. Molecular structure of **4a** with thermal displacement parameters drawn at 50% probability. All hydrogen atoms are omitted for clarity. Selected bond lengths [Å] and angles [°]: Ag1−C1 2.085(2), Ag1−P1 2.3434(6), B1−C2 1.654(3), N1−C1−N2 105.60(17), C1−Ag1−P1 166.81(6), N1−C1−Ag1 132.49(15), N2−C1−Ag1 121.51(14), N1−C1−Ag1−P1 −160.71(16), N2−C1−Ag1−P1 11.0(4).

4.4 [(WCA-IDipp)Cu(PPh_3)] (4b)

Compound	4b
**Identification code**	2096238
**Empirical formula**	$C_{63}H_{50}BCuF_{15}N_2P$
**Formula weight**	1225.37
**Temperature**	100(2) K
**Wavelength**	0.71073 Å
**Instrument (scan mode)**	XtaLAB Synergy, Single source at off-set/far, HyPix (ω scan)
**Crystal system**	Monoclinic
**Space group**	$P2_1/n$
**Unit cell dimensions**	a = 11.0151(2) Å α = 90°<br>b = 26.0435(5) Å β = 92.010(2)°<br>c = 19.0823(3) Å γ = 90°
**Volume**	5470.80(17) $Å^3$
**Z**	4
**Density (calculated)**	1.488 Mg/m^3
**Absorption coefficient**	0.524 mm^{-1}
**F(000)**	2504
**Crystal habitus**	irregular (colourless)
**Crystal size**	0.350 x 0.220 x 0.170 mm^3
**Theta range for data collection**	2.578 to 31.522°
**Index ranges**	-14<=h<=16, -37<=k<=37, -28<=l<=28
**Reflections collected**	223069
**Independent reflections**	17707 [R(int) = 0.0365]
**Completeness to theta = 25.242°**	99.9 %
**Absorption correction**	Gaussian
**Max. and min. transmission**	1.000 and 0.410
**Refinement method**	Full-matrix least-squares on F^2
**Data / restraints / parameters**	17707 / 0 / 756
**Goodness-of-fit on F^2**	1.043
**Final R indices [I>2sigma(I)]**	R1 = 0.0392, wR2 = 0.1058
**R indices (all data)**	R1 = 0.0492, wR2 = 0.1104
**Largest diff. peak and hole**	0.768 and -0.564 e.$Å^{-3}$
**Crystallisation Details:**	Saturated THF solution
**Solution:**	SHELXT-2014/5 (G. M. Sheldrick, Acta Cryst., 2015, A71, 3-8)
**Refinement:**	SHELXL-2018/3 (G. M. Sheldrick, Acta Cryst. (2008), A64, 112-122)
**Measurement and Refinement Details:**	-

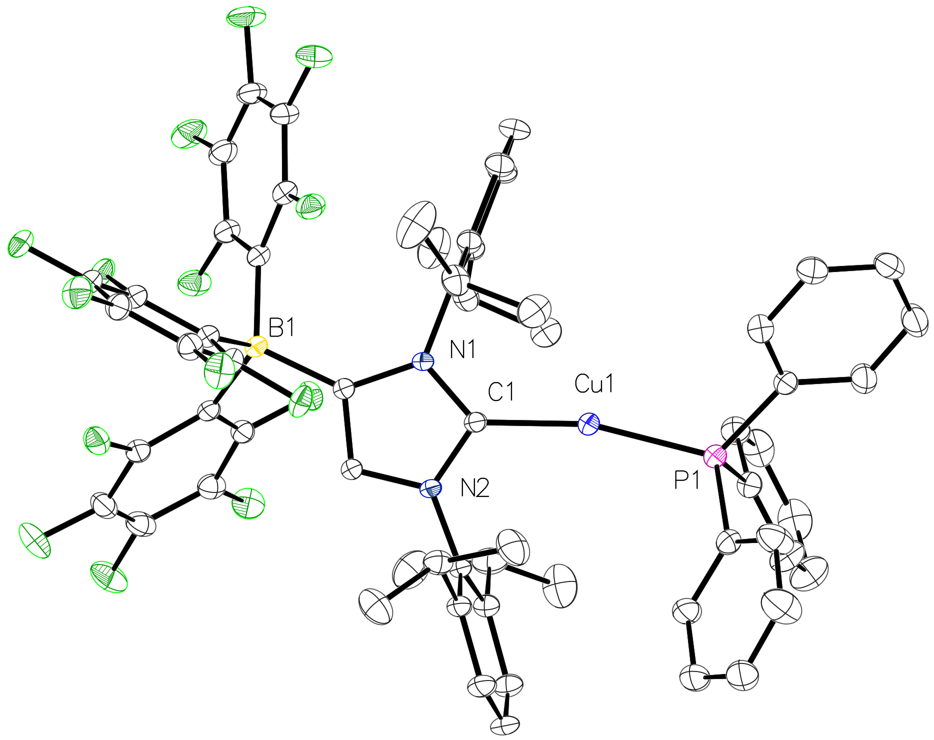

Figure S4. Molecular structure of **4b** with thermal displacement parameters drawn at 50% probability. All hydrogen atoms are omitted for clarity. Selected bond lengths [Å] and angles [°]: Cu1−C1 1.9063(12), Cu1−P1 2.1926(4), B1−C2 1.6519(18), N1−C1−N2 104.66(10), C1−Cu1−P1 166.23(4), N1−C1−Cu1 131.92(9), N2−C1−Cu1 123.19(9), N1−C1−Cu1−P1 −161.19(11), N2−C1−Cu1−P1 12.4(3).

4.5 [(WCA-IDipp)Ag(η^2-Tol)] (5a)

Compound	**5a**·0.5toluene
**Identification code**	2096239
**Empirical formula**	$C_{55.5}H_{47}AgBF_{15}N_2$
**Formula weight**	1145.63
**Temperature**	100(2) K
**Wavelength**	1.54184 Å
**Instrument (scan mode)**	SuperNova, Dual, Cu at home/near, Atlas (ω scan)
**Crystal system**	Monoclinic
**Space group**	$P2_1/c$
**Unit cell dimensions**	a = 10.59946(14) Å α= 90°<br>b = 18.4853(2) Å β= 101.6348(13)°<br>c = 25.6698(3) Å γ = 90°
**Volume**	4926.25(11) $Å^3$
**Z**	4
**Density (calculated)**	1.545 Mg/m^3
**Absorption coefficient**	4.147 mm^{-1}
**F(000)**	2324
**Crystal habitus**	plate (colourless)
**Crystal size**	0.258 x 0.114 x 0.057 mm^3
**Theta range for data collection**	2.967 to 74.267°
**Index ranges**	-13<=h<=13, -22<=k<=23, -31<=l<=31
**Reflections collected**	77174
**Independent reflections**	9966 [R(int) = 0.0306]
**Completeness to theta = 67.684°**	100.0%
**Absorption correction**	Semi-empirical from equivalents
**Max. and min. transmission**	1.00000 and 0.69979
**Refinement method**	Full-matrix least-squares on F^2
**Data / restraints / parameters**	9966 / 0 / 713
**Goodness-of-fit on F^2**	1.047
**Final R indices [I>2sigma(I)]**	R1 = 0.0241, wR2 = 0.0662
**R indices (all data)**	R1 = 0.0250, wR2 = 0.0668
**Largest diff. peak and hole**	0.412 and -0.663 e.$Å^{-3}$
**Solution:**	SHELXT 2014/5 (Sheldrick, 2014)
**Refinement:**	SHELXL-2018/3 (G. M. Sheldrick, Acta Cryst., 2015, C71, 3-8)
**Interface:**	OLEX2 v1.3 (O. V. Dolomanov et al., J. Appl. Cryst., 2009, 42, 339-341)
**Measurement and Refinement Details:**	One molecule of toluene is disordered over an inversion center and was refined accordingly.

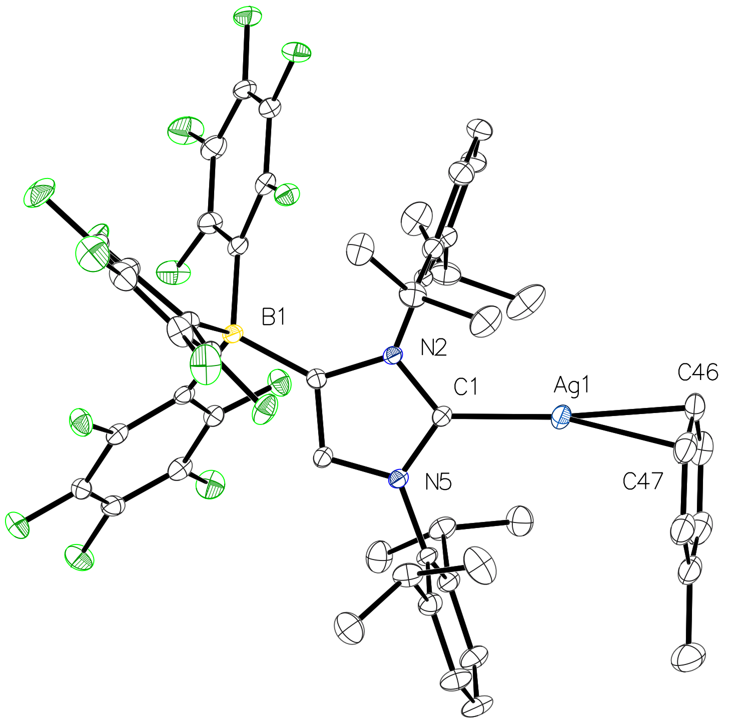

Figure S5. Molecular structure of **5a**·0.5toluene with thermal displacement parameters drawn at 50% probability. All hydrogen atoms and non-coordinating toluene are omitted for clarity. Selected bond lengths [Å] and angles [°]: Ag1−C1 2.0839(14), Ag1−C46 2.3382(17), Ag1−C47 2.4056(17), B1−C2 1.658(2), N1−C1−N2 105.04(12).

4.6 [(WCA-IDipp)Cu(η^2-Tol)] (5b)

Compound	**5b**·0.5toluene
**Identification code**	2096240
**Empirical formula**	$C_{55.5}H_{47}BCuF_{15}N_2$
**Formula weight**	1101.30
**Temperature**	100(2) K
**Wavelength**	1.54184 Å
**Instrument (scan mode)**	SuperNova, Dual, Cu at home/near, Atlas (ω scan)
**Crystal system**	Monoclinic
**Space group**	$P2_1/c$
**Unit cell dimensions**	a = 10.6868(2) Å α = 90°<br>b = 18.2795(4) Å β = 101.979(2)°<br>c = 25.5863(6) Å γ = 90°
**Volume**	4889.42(19) $Å^3$
**Z**	4
**Density (calculated)**	1.496 Mg/m^3
**Absorption coefficient**	1.501 mm^{-1}
**F(000)**	2252
**Crystal habitus**	needle (colourless)
**Crystal size**	0.666 x 0.207 x 0.154 mm^3
**Theta range for data collection**	2.994 to 67.068°
**Index ranges**	-12<=h<=12, -21<=k<=21, -22<=l<=30
**Reflections collected**	35210
**Independent reflections**	8737 [R(int) = 0.0305]
**Completeness to theta = 67.068°**	99.9 %
**Absorption correction**	Semi-empirical from equivalents
**Max. and min. transmission**	1.00000 and 0.60830
**Refinement method**	Full-matrix least-squares on F^2
**Data / restraints / parameters**	8737 / 0 / 721
**Goodness-of-fit on F^2**	1.028
**Final R indices [I>2sigma(I)]**	R1 = 0.0332, wR2 = 0.0849
**R indices (all data)**	R1 = 0.0375, wR2 = 0.0886
**Largest diff. peak and hole**	0.307 and -0.489 e.$Å^{-3}$
**Solution:**	SHELXT 2018/2 (G. M. Sheldrick, Acta Cryst., 2015, A71, 3-8)
**Refinement:**	SHELXL-2018/3 (G. M. Sheldrick, Acta Cryst., 2015, C71, 3-8)
**Interface:**	OLEX2 v1.3 (O. V. Dolomanov et al., J. Appl. Cryst., 2009, 42, 339-341)
**Measurement and Refinement Details:**	One toluene molecule is disordered over an inversion center and was refined accordingly. The hydrogens on the coordinating toluene atoms H46 and H47 (bound to C46 and C47 respectively) were refined freely.

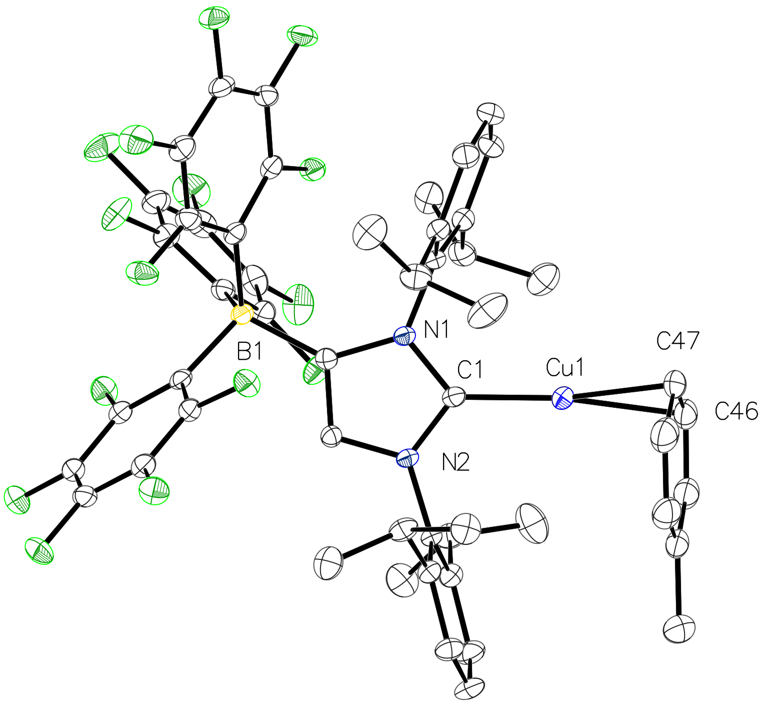

Figure S6. Molecular structure of **5b**·0.5toluene with thermal displacement parameters drawn at 50% probability. All hydrogen atoms and non-coordinating toluene are omitted for clarity. Selected bond lengths [Å] and angles [°]: Cu1−C1 1.9016(17), Cu1-C46 2.2013(19), Cu1−C47 2.1220(19), B1−C2 1.662(2), N1−C1−N2 103.94(13).

4.7 [(WCA-IDipp)Ag(μ-Cl_2)Ru(PPh_3)(η^6-*p*-cymene)] (6a·CH_2Cl_2)

Compound	6a·CH_2Cl_2
Identification code	2096241
Empirical formula	$C_{73}H_{64}AgBCl_2F_{15}N_2PRu\cdot0.46(CH_2Cl_2)$
Formula weight	1615.37
Temperature	100(2) K
Wavelength	0.71073 Å
Instrument (scan mode)	XtaLAB Synergy, Single source at off-set/far, HyPix (ω scan)
Crystal system	Triclinic
Space group	*P*-1
Unit cell dimensions	a = 11.0817(2) Å α = 102.476(2)°<br>b = 14.2704(2) Å β = 92.320(2)°<br>c = 22.4630(2) Å γ = 96.026(2)°
Volume	3441.95(9) $Å^3$
Z	2
Density (calculated)	1.559 Mg/m^3
Absorption coefficient	0.726 mm^{-1}
F(000)	1631
Crystal habitus	irregular (red)
Crystal size	0.255 x 0.201 x 0.179 mm^3
Theta range for data collection	2.126 to 38.343°
Index ranges	-19<=h<=19, -24<=k<=24, -38<=l<=38
Reflections collected	276022
Independent reflections	36232 [R(int) = 0.0284]
Completeness to theta = 25.242°	99.9 %
Absorption correction	Gaussian
Max. and min. transmission	1.000 and 0.408
Refinement method	Full-matrix least-squares on F^2
Data / restraints / parameters	36232 / 43 / 1033
Goodness-of-fit on F^2	1.176
Final R indices [I>2sigma(I)]	R1 = 0.0428, wR2 = 0.0880
R indices (all data)	R1 = 0.0536, wR2 = 0.0909
Largest diff. peak and hole	1.972 and -1.712 e.$Å^{-3}$
Crystallisation Details	CH_2Cl_2/*n*-hexane
Solution:	SHELXT 2018/2 (G. M. Sheldrick, Acta Cryst., 2015, A71, 3-8)
Refinement:	SHELXL-2018/3 (G. M. Sheldrick, Acta Cryst., 2015, C71, 3-8)
Measurement and Refinement Details:	Two phenyl rings of PPh_3 are disordered over two positions and were treated accordingly. The dichloromethane shows two not fully occupied positions (9% and

	38%) and was therefore refined employing free variables for the occupation and restraints to yield a stable refinement. The dichloromethane most likely evaporates from the crystals upon removal from the mother liquor, as evident by rapid dulling of the surface.

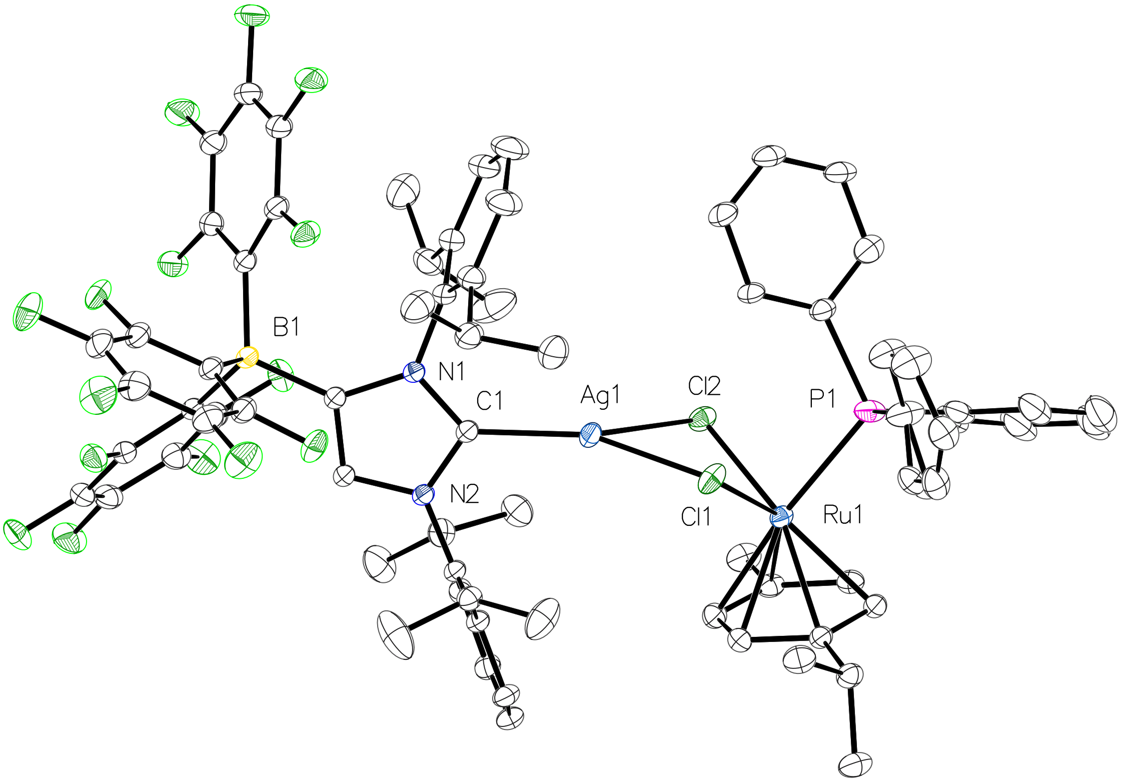

Figure S7. Molecular structure of **6a** with thermal displacement parameters drawn at 50% probability. All hydrogen atoms, DCM molecules and disordered phenyl rings are omitted for clarity. Selected bond lengths [Å] and angles [°]: Ag1−C1 2.0907(14), Ag1−Cl1 2.5287(4), Ag1−Cl2 2.6727(4), Ru1−P1 2.3897(4), Ru1−Cl1 2.4135(4), Ru1−Cl2 2.4156(3), B1−C2 1.646(2), N1−C1−N2 104.73(11), C1−Ag1−Cl1 147.85(4), C1−Ag1−Cl2 131.98(4).

4.8 [(WCA-IDipp)Cu(μ-Cl_2)Ru(PPh_3)(η^6-*p*-cymene)] (6b·CH_2Cl_2)

Compound	6b·CH_2Cl_2
Identification code	2096242
Empirical formula	$C_{74}H_{66}BCl_4CuF_{15}N_2PRu$
Formula weight	1616.47
Temperature	100(2) K
Wavelength	1.54184 Å
Instrument (scan mode)	SuperNova, Dual, Cu at home/near, Atlas (ω scan)
Crystal system	Orthorhombic
Space group	*Pbca*
Unit cell dimensions	a = 26.0326(3) Å α = 90°<br>b = 19.2403(3) Å β = 90°<br>c = 28.1513(3) Å γ = 90°
Volume	14100.2(3) $Å^3$
Z	8
Density (calculated)	1.523 Mg/m^3
Absorption coefficient	4.446 mm^{-1}
F(000)	6560
Crystal habitus	block (orange)
Crystal size	0.34 x 0.269 x 0.202 mm^3
Theta range for data collection	3.140 to 67.069°
Index ranges	-30<=h<=31, -22<=k<=18, -33<=l<=30
Reflections collected	35517
Independent reflections	12559 [R(int) = 0.0241]
Completeness to theta = 67.069°	99.8 %
Absorption correction	Gaussian
Max. and min. transmission	1.000 and 0.392
Refinement method	Full-matrix least-squares on F^2
Data / restraints / parameters	12559 / 12 / 922
Goodness-of-fit on F^2	1.038
Final R indices [I>2sigma(I)]	R1 = 0.0461, wR2 = 0.1262
R indices (all data)	R1 = 0.0538, wR2 = 0.1351
Largest diff. peak and hole	1.878 and -1.028 e.$Å^{-3}$
Solution:	ShelXT (Sheldrick, 2015)
Refinement:	SHELXL-2018/3 (G. M. Sheldrick, Acta Cryst. (2008), A64, 112-122)
Measurement and Refinement Details:	One molecule of DCM is disordered over two positions and was refined accordingly. The isopropyl group of cymene may be split but a second position could not be refined satisfactorily. Therefore the group was restrained employing the ISOR command.

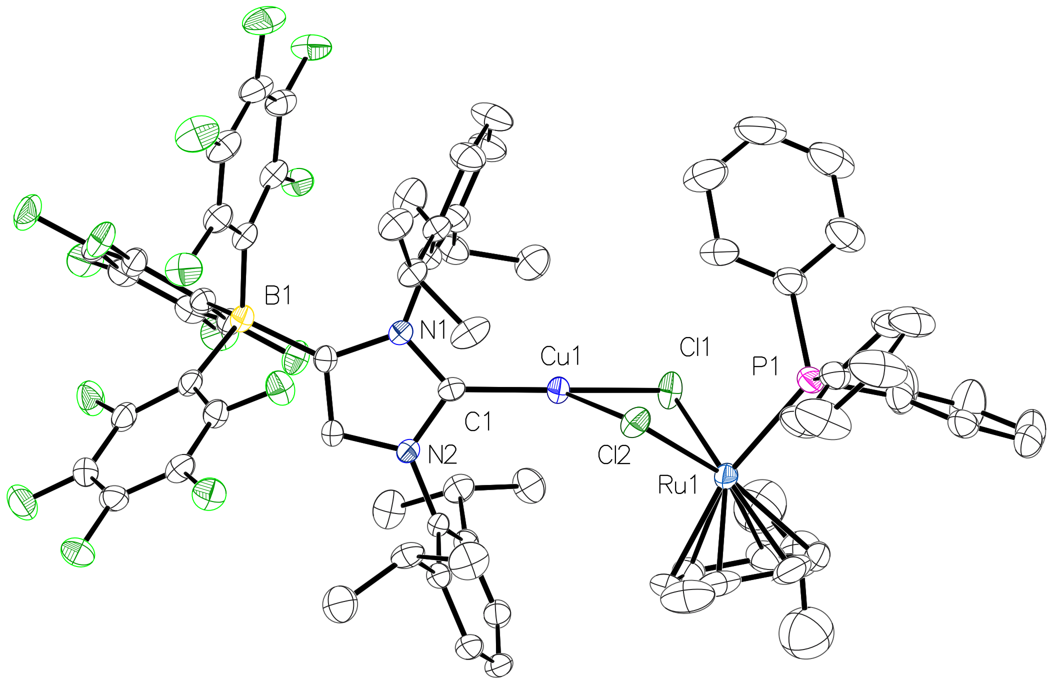

Figure S8. Molecular structure of **6b** with thermal displacement parameters drawn at 50% probability. All hydrogen atoms and one disordered DCM molecule are omitted for clarity. Selected bond lengths [Å] and angles [°]: Cu1−C1 1.898(3), Cu1−Cl1 2.2606(9), Cu1−Cl2 2.5025(9), Ru1−P1 2.3624(9), Ru1−Cl1 2.4147(9), Ru1−Cl2 2.4088(9), B1−C2 1.654(4), N1−C1−N2 104.0(3), C1−Cu1−Cl1 151.61(10), C1−Cu1−Cl2 123.34(10).

4.9 [(WCA-IDipp)Ag(μ-I_2)Ru(PPh_3)(η^6-*p*-cymene)] (7·solvent)

Compound	**7**·[solvent]
**Identification code**	2096243
**Empirical formula**	$C_{74}H_{66}AgBCl_2F_{15}I_2N_2PRu$ + [solvent]
**Formula weight**	1843.70
**Temperature**	100(2) K
**Wavelength**	1.54184 Å
**Instrument (scan mode)**	SuperNova, Dual, Cu at home/near, Atlas (ω scan)
**Crystal system**	Monoclinic
**Space group**	$P2_1/n$
**Unit cell dimensions**	a = 12.9659(2) Å α = 90°<br>b = 18.4426(3) Å β = 96.5847(16)°<br>c = 31.7867(6) Å γ = 90°
**Volume**	7550.9(2) $Å^3$
**Z**	4
**Density (calculated)**	1.622 Mg/m^3
**Absorption coefficient**	11.655 mm^{-1}
**F(000)**	3640
**Crystal habitus**	needle (red)
**Crystal size**	0.68 x 0.16 x 0.15 mm^3
**Theta range for data collection**	2.775 to 67.077°
**Index ranges**	-14<=h<=15, -22<=k<=13, -37<=l<=36
**Reflections collected**	28582
**Independent reflections**	13455 [R(int) = 0.0429]
**Completeness to theta = 67.077°**	100.0 %
**Absorption correction**	Analytical
**Max. and min. transmission**	0.305 and 0.074
**Refinement method**	Full-matrix least-squares on F^2
**Data / restraints / parameters**	13455 / 12 / 903
**Goodness-of-fit on F^2**	1.030
**Final R indices [I>2sigma(I)]**	R1 = 0.0438, wR2 = 0.1137
**R indices (all data)**	R1 = 0.0510, wR2 = 0.1199
**Largest diff. peak and hole**	1.334 and -1.656 e.$Å^{-3}$
**Solution:**	ShelXT (Sheldrick, 2015)
**Refinement:**	SHELXL-2018/3 (G. M. Sheldrick, Acta Cryst. (2008), A64, 112-122)
**Measurement and Refinement Details:**	One isopropylgroup of the IDipp ligand is most likely split. A second position could not be refined satisfactorily and therefore ISOR restraints were applied. A solvent accessible canal was found that most likely contains a combination of dichloromethane and pentane. Since the solvent

	molecules are disordered along the axis of the canal, a satisfying refinement could not be achieved. Therefore the electron density attributed to the canal was mathematically removed using the "Solvent Mask" as implemented in the OLEX2 program suite.

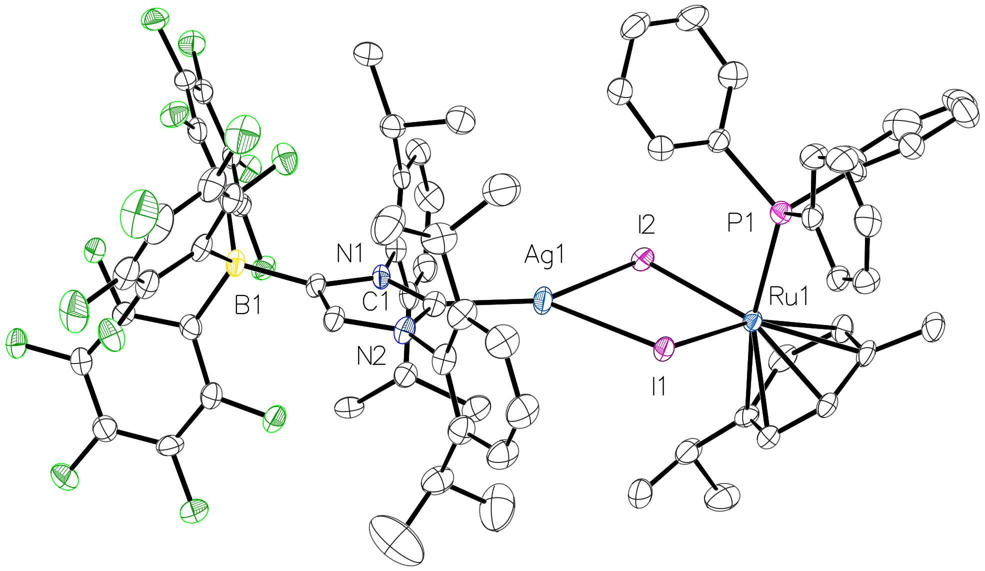

Figure S9. Molecular structure of **7** with thermal displacement parameters drawn at 50% probability. All hydrogen atoms and solvent molecules are omitted for clarity. Selected bond lengths [Å] and angles [°]: Ag1−C1 2.139(5), Ag1−I1 2.8619(5), Ag1−I2 2.8310(5), Ru1−P1 2.3677(12), Ru1−I1 2.7263(4), Ru1−I2 2.7236(4), B1−C2 1.645(7), N1−C1−N2 104.8(4), C1−Ag1−I1 132.64(13), C1−Ag1−I2 144.92(13).

4.10 [(WCA-IDipp)Cu(Cl)][(Ru(η^6-*p*-cymene))$_2$(μ-Cl$_3$)]

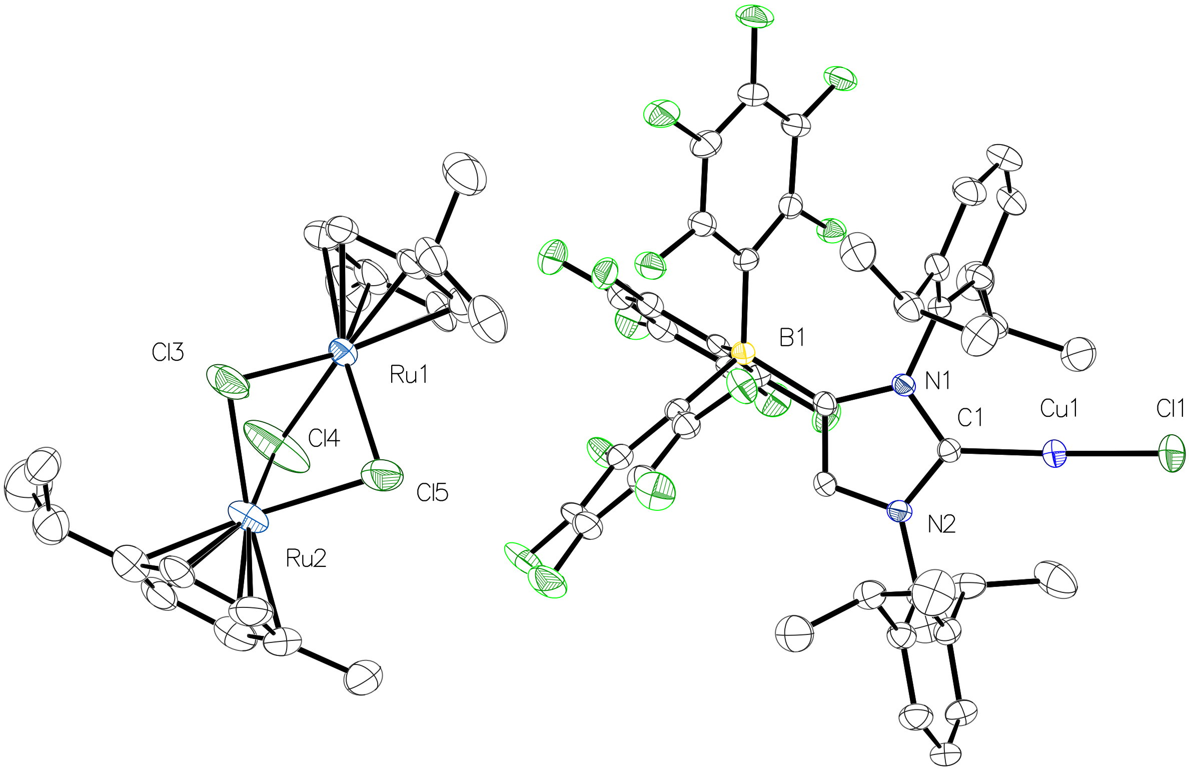

Figure S10. Preliminary composition of [(WCA-IDipp)Cu(Cl)][(Ru(*p*-cymene))$_2$(μ-Cl$_3$)]. The asymmetric unit contains two sets of NHC and Ru molecules. **Because of weak data, major disorder and possible twinning no data suitable for publication could be obtained**. However the preliminary X-ray diffraction analysis supports the suggested composition of the compound.

4.11 [Li(THF)$_4$][((WCA-IDipp)Cu)$_2$(μ-Cl)]·1.5C_6H_5Cl

Under exclusion of light, a Schlenk flask is charged with CuCl (39.7 mg, 0.4 mmol, 2 equiv.) dissolved in THF (6 mL) and [(WCA-IDipp)Li(toluene)] (**1**, 200 mg, 0.2 mmol, 1 equiv.) dissolved in THF (6 mL) is added dropwise. The solution is stirred for 2.5 h at room temperature and then filtered through a pad of Celite®. The solvent is removed under high vacuum and the product isolated as a colorless solid (154 mg, 0.075 mmol, 38%).

**Compound**	**[Li(THF)$_4$][((WCA-IDipp)Cu)$_2$(μ-Cl)] ·1.5C_6H_5Cl**
**Identification code**	2096244
**Empirical formula**	$C_{115}H_{109.5}B_2Cl_{2.5}Cu_2F_{30}LiN_4O_4$
**Formula weight**	2425.82
**Temperature**	100(2) K
**Wavelength**	1.54184 Å
**Instrument (scan mode)**	Oxford Diffraction Xcalibur, Atlas, Nova (ω scan)
**Crystal system**	Monoclinic
**Space group**	$P2_1/n$
**Unit cell dimensions**	a = 12.0560(2) Å α = 90°<br>b = 40.2178(5) Å β = 97.7819(10)°<br>c = 23.1371(3) Å γ = 90°
**Volume**	11115.0(3) Å^3
**Z**	4
**Density (calculated)**	1.450 Mg/m^3
**Absorption coefficient**	1.939 mm^{-1}
**F(000)**	4972
**Crystal habitus**	prism (colourless)
**Crystal size**	0.20 x 0.10 x 0.03 mm^3
**Theta range for data collection**	3.820 to 76.388°
**Index ranges**	-14<=h<=15, -50<=k<=50, -29<=l<=29
**Reflections collected**	267713
**Independent reflections**	23186 [R(int) = 0.0655]
**Completeness to theta = 67.684°**	100.0 %
**Absorption correction**	Semi-empirical from equivalents
**Max. and min. transmission**	1.00000 and 0.82293
**Refinement method**	Full-matrix least-squares on F^2
**Data / restraints / parameters**	23186 / 133 / 1493
**Goodness-of-fit on F^2**	1.038
**Final R indices [I>2sigma(I)]**	R1 = 0.0431, wR2 = 0.1126
**R indices (all data)**	R1 = 0.0528, wR2 = 0.1191

**Largest diff. peak and hole**	1.046 and -0.817 e.Å^{-3}
**Solution:**	SHELXS-97 (Sheldrick, 1990)
**Refinement:**	SHELXL-2018/3 (G. M. Sheldrick, Acta Cryst. (2008), A64, 112-122)
**Measurement and Refinement Details:**	One molecule of chlorobenzene is disordered over an inversion center and was refined employing the FragmentDB as implemented in the OLEX2 program suite.

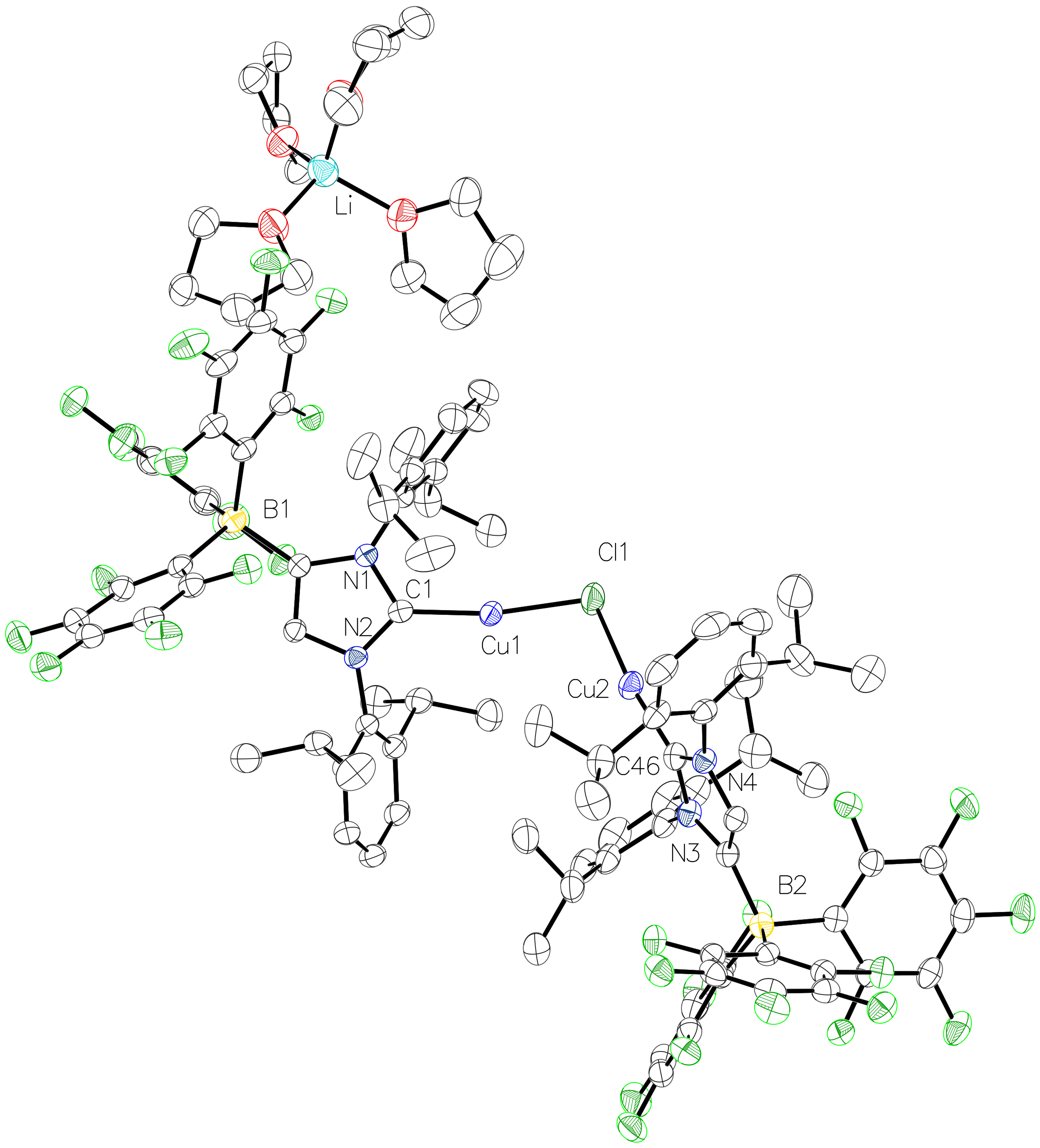

Figure S11. Molecular structure of [Li(THF)$_4$][((WCA-IDipp)Cu)$_2$(μ-Cl)]·1.5C_6H_5Cl with thermal displacement parameters drawn at 50% probability. All hydrogen atoms and 1.5 molecules chlorobenzene are omitted for clarity. Selected bond lengths [Å] and angles [°]: Cu1−Cl1 2.1353(6), Cu2−Cl1 2.1282(6), Cu1−C1 1.8847(19), Cu2−C46 1.879(2), B1−C2 1.645(3), B2−C47 1.651(3), Cu1−Cl1−Cu2 105.25(3), C1−Cu1−Cl1 170.00(6), C46−Cu2−Cl1 172.35(6), N1−C1−N2 104.72(16), N3−C46−N4 104.41(17), Cu−C1−N1 122.44(14), Cu−C1−N2 132.84 (14), Cu2–C46–N3 128.10(14), Cu2−C46−N4 127.34(15).
